# HSP70 Interactome‐Mediated Proteolysis Targeting Chimera (HSP70‐PROTAC) for Ferroptosis‐Driven Cancer Treatment

**DOI:** 10.1002/advs.202513655

**Published:** 2025-12-12

**Authors:** Jinyun Dong, Yulong Li, Hui Liang, Zumei Wu, Shiqun Wang, Yichao Wang, Jieyu Xu, Yanning Lan, Maohua Cai, Guangzhao Pan, Haiyan Yang, Kai Miao, Zhe‐Sheng Chen, Fangfang Tao, Xuelei Ma, Jiang‐Jiang Qin

**Affiliations:** ^1^ Center for Innovative Drug Research Hangzhou Institute of Medicine (HIM) Chinese Academy of Sciences Hangzhou Zhejiang 310022 China; ^2^ Zhejiang Cancer Hospital Hangzhou Institute of Medicine (HIM) Chinese Academy of Sciences Hangzhou Zhejiang 310022 China; ^3^ Department of Pharmaceutical Sciences Institute for Biotechnology College of Pharmacy and Health Sciences St. John's University Queens NY 11439 USA; ^4^ MOE Frontier Science Centre for Precision Oncology University of Macau Macau SAR 999078 China; ^5^ Zhejiang Key Laboratory of Blood‐Stasis‐Toxin Syndrome Zhejiang Chinese Medical University China Collaborative Graduate School of Traditional Chinese Medicine Hangzhou Zhejiang China; ^6^ Department of Biotherapy Cancer Center and State Key Laboratory of Biotherapy West China Hospital Sichuan University Chengdu Sichuan China

**Keywords:** cancer, ferroptosis, glutathione peroxidase 4, heat shock protein 70, targeted protein degradation

## Abstract

Targeted protein degradation (TPD) represents a transformative therapeutic paradigm that harnesses the cellular degradation machinery to pharmacologically eliminate disease‐causing proteins with aberrant expression. This work here reports the first design of an HSP70 interactome‐mediated proteolysis targeting chimera (HSP70‐PROTAC) for the degradation of the intracellular therapeutically relevant proteins via dual processes of ubiquitin‐proteasomal degradation (UPS) and chaperone‐mediated autophagy (CMA). By hijacking the highly expressed heat shock cognate protein (Hsc70) isoform complex in tumor tissues to glutathione peroxidase 4 (GPX4) protein, this work successfully develops an HSP70‐PROTAC molecule GDAz‐3 that potently and rapidly eliminates GPX4 in HT1080 cells, thereby triggering ferroptosis with high selectivity. Correspondingly, GDAz‐3 exhibits a remarkable tumor‐inhibitory effect in the HT1080 xenograft tumor mouse model without obvious toxicity. In addition, this work demonstrates the versatility of HSP70‐based PROTACs by effectively degrading additional endogenous bromodomain‐containing protein 4 (BRD4) in cancer cells. More importantly, the degradation of GPX4 mediated by GDAz‐3 occurs with comparable efficiency in CRBN/VHL‐knockdown cells and 786‐O cells intrinsically lacking VHL expression, which facilitates expanding the application scope and overcoming drug resistance of traditional PROTAC. These findings suggest that HSP70‐PROTAC is a novel and feasible strategy for the future development of TPD technology.

## Graphical abstract

This study reports a novel targeted protein degradation strategy termed “HSP70‐PROTAC” that recruits Hsc70 complex to a target protein for inducing degradation. Among them, GDAz‐3 exhibits effective GPX4 degradation activity via UPS/CMA processes, triggering ferroptosis‐driven anticancer activity in vitro and in vivo. Furthermore, GDAz‐3 shows comparable GPX4 degradation efficiency in CRBN/VHL‐knockdown cells and 786‐O cells intrinsically lacking VHL expression.

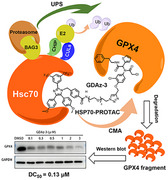

## 1 Introduction

Ferroptosis, a non‐apoptotic form of cell death, is characterized by iron‐dependent membrane lipid peroxidation. Emerging studies have shown that ferroptosis is closely related to the pathophysiological processes of numerous diseases such as tumors and neurodegenerative diseases.^[^ ^1^, ^2^ ^]^ Given its distinctive mechanism of cell death, the regulation of cell ferroptosis has recently attracted considerable attention as a potential treatment strategy for multiple diseases, especially for eradicating aggressive malignancies that are resistant to traditional therapies.^[^ ^3^ ^]^ As research on the mechanism of ferroptosis deepens, multiple pathways have been identified to be involved in ferroptosis initiation including iron homeostasis, glutathione (GSH) metabolism, and lipid peroxidation. Among these pathways, the system Xc^−^ (Sxc, cystine/glutamate antiporter)/GSH/GPX4 (glutathione peroxidase 4) axis has been considered as the primary pathway to protect cells from oxidative stress and subsequent ferroptosis.^[^ ^4^, ^5^ ^]^ GPX4, a unique selenoprotein antioxidant enzyme in mammals, is particularly notable for its pivotal role in catalyzing the transformation of toxic phospholipid hydroperoxides (PL‐OOH) into their corresponding nontoxic phospholipid alcohols (PL‐OH), thereby protecting cells from oxidative damage. Conversely, depletion or inactivation of GPX4 activity leads to an elevation in the level of intracellular lipid peroxide (LPO), resulting in cellular membrane damage and ferroptosis. GPX4 is highly expressed in many tumor types and is closely associated with tumor progression and drug resistance.^[^ ^6^ ^]^ Consistently, in our recently published study, we also confirmed that GPX4 is a critical mechanism for ferroptosis evasion in gastric adenocarcinoma (GAC) cells by integrating multi‐omics approaches.^[^ ^7^ ^]^ Growing evidence has validated that targeting GPX4‐induced ferroptosis is a promising strategy for cancer treatment and overcoming drug resistance in cancer cells.^[^ ^6^ ^]^

In recent years, targeting GPX4 has been of high interest, and several compounds have been reported to possess the potential to inactivate GPX4 by forming a covalent bond with its catalytic selenocysteine residue (U46) including RSL3, ML162, and ML210.^[^ ^8^ ^]^ These compounds have demonstrated significant antitumor potency at the cellular level through direct inhibition of GPX4 activity and induction of ferroptosis. However, the application of GPX4 inhibitors in vivo (in living organisms) still poses several challenges, particularly the risk of excessive systemic toxicity due to the nonselective inhibition of GPX4 (limited selectivity for ferroptosis) and unsatisfactory in vivo antitumor effects.^[^ ^9^ ^]^ For example, although RSL3 is a potent and irreversible inhibitor of GPX4, it is not typically suitable for in vivo use due to its low ferroptosis selectivity (SI = 3.1), poor solubility, and unfavorable pharmacokinetic properties (ADME) accompanied by metabolic instability;^[^ ^10^, ^11^ ^]^ similarly, ML162 also suffers from poor metabolic stability and low selectivity.^[^ ^12^ ^]^ These deficiencies in selectivity and pharmacokinetic properties may be partly ascribed to the relatively shallow binding site of GPX4, which restricts the potential interactions between GPX4 and its inhibitors. Consequently, there is an unmet need to develop novel therapeutic strategies for more effective targeting of GPX4.

Targeted protein degradation (TPD) mediated by small molecules has recently emerged as a highly sought‐after therapeutic modality in drug discovery because of its potential to specifically eliminate intractable disease‐causing proteins within cells.^[^ ^13^ ^]^ In contrast to the traditional inhibition strategy, TPD is independent of the occupancy‐driven target inhibition mechanism and offers a potential approach for targeting the “undruggable” proteins that were traditionally considered inaccessible to small‐molecule intervention.^[^ ^14^ ^]^ In addition, TPD could achieve selective degradation using warhead ligands with nonselective binding characteristics as demonstrated by proteolysis‐targeting chimera (PROTAC).^[^ ^15^ ^]^ To date, numerous strategies to degrade target proteins have emerged, but each TPD strategy has its unique advantages, disadvantages, and applicable scope, which we have comprehensively summarized in our previous work.^[^ ^13^ ^]^ For example, a major bottleneck with TPD is the limited availability of E3 ligase recruiters, despite the existence of more than 600 E3 ligases in human cells. In addition, it should be noted that not every protein of interest (POI)‐E3 ligase combination results in ubiquitination competent complex formation.^[^ ^16^, ^17^ ^]^

**Figure 1:**
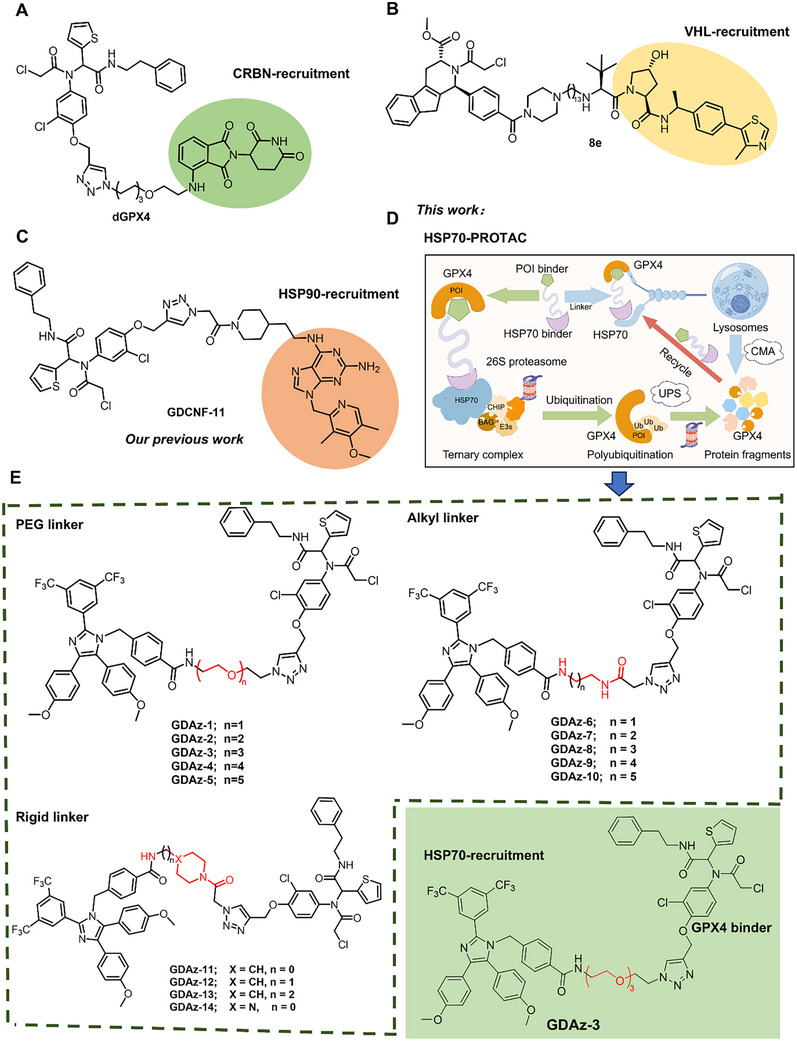
The reported active GPX4‐targeting degraders and schematic diagram of HSP70‐PROTAC to degrade POI via UPS and CMA. A) Structure of representative CRBN‐based GPX4‐targeting PROTAC dGPX4. B) Structure of representative VHL‐based GPX4‐targeting PROTAC 8e. C) Structure of representative HSP90‐based GPX4‐targeting HIM‐PROTAC GDCNF‐11. D) Schematic diagram of heat shock protein 70 interactome‐mediated proteolysis targeting chimera (HSP70‐PROTAC) to degrade POI via USP and CMA dual processes. E) The structures of the synthesized GPX4‐targeting HSP70‐PROTACs including GDAz‐3.

As aforementioned, GPX4 represents a challenging target because it has a shallow active site that is not amenable to interaction with small‐molecule inhibitors. As a result, TPD, particularly PROTAC technology, may offer an effective strategy for selectively degrading GPX4 to induce ferroptosis, and several GPX4‐targeting PROTACs have recently been reported.^[^ ^18^, ^19^, ^20^, ^21^, ^22^, ^23^, ^24^ ^]^ Currently, the majority of these PROTACs utilize either of the two E3 ligases, cereblon (CRBN) or von Hippel‐Lindau (VHL), for targeted protein degradation. Some of them have shown remarkable efficiency in degrading GPX4, including the CRBN‐based degraders dGPX4, DC‐2, A7, and PD‐4, as well as the VHL‐based degraders 8e and ZX703 (the representative structures depicted in **Figure** **1**A,B).^[^ ^18^, ^19^, ^20^, ^21^, ^22^, ^23^ ^]^ However, traditional PROTACs still face several challenges, including 1) CRBN and VHL display distinct and restricted substrate specificities for executing TPD;^[^ ^25^ ^]^ 2) the single E3 ubiquitin ligase VHL or CRBN involved in protein degradation often causes low tissue specificity, adaptive resistance, and off‐target effects in cells due to their widespread distribution in normal tissues and tumors;^[^ ^26^ ^]^ 3) VHL is a tumor suppressor protein, which is frequently mutated in several tumor cells.^[^ ^26^ ^]^ Heat shock proteins (HSPs) are essential molecular chaperones that play critical roles in stabilizing protein structures, facilitating the repair or degradation of damaged proteins, and maintaining proteostasis and cellular functions.^[^ ^27^ ^]^ They have hundreds of “client proteins”, including kinases, transcription factors, steroid hormone receptors, and E3 ubiquitin ligases. Heat shock proteins are highly expressed in almost all cancers, which may endow HSP‐binding compounds with unique tumor‐selective pharmacokinetics. In our previous study, we developed HIM‐PROTAC‐based protein degraders that chemically knockdown endogenous GPX4 by recruiting multiple E3 ubiquitin ligases through 90‐kDa heat shock protein (HSP90).^[^ ^28^ ^]^ The active molecule GDCNF‐11 can effectively degrade GPX4, selectively trigger ferroptosis, and potentially circumvent resistance (Figure 1C). However, it still confronts certain challenges, such as a relatively slow degradation rate. This may be ascribed to its degradation mechanism where the degradation system requires sufficient time to recruit E3 ligases and proteasomes after active molecules bind to HSP90. Therefore, the development of a novel TPD strategy with superior degradation efficiency is important for improving therapeutic outcomes.

Heat shock protein 70 (HSP70) is another extensively studied group of heat shock protein that is highly upregulated in various cancer cells.^[^ ^29^ ^]^ However, when compared with HSP90, HSP70 has been proposed as a master regulator of protein degradation, as it plays an essential role in broad substrate degradation not only through the ubiquitin‐proteasome system (UPS) but also through chaperone‐mediated autophagy (CMA).^[^ ^30^ ^]^ HSP70 directs its targets to the 26S proteasome, depending on its interactions with co‐chaperone proteins such as the Bcl‐2‐associated athanogene (BAG) proteins (in particular, BAG1 and BAG3) and ubiquitylation enzymes. The BAG proteins establish a physical connection between HSP70s (specifically Hsc70/Hsp72(Hsp70)) and the 26S proteasome, which is beneficial for facilitating the delivery of HSP70‐bound target proteins to the proteasome for degradation.^[^ ^31^ ^]^ Inspired by these observations, we herein first propose a novel TPD strategy (termed “HSP70‐PROTAC,” Figure 1D) that bridges the target protein to the heat shock protein 70 complex, thereby triggering both UPS and CMA degradation processes. As a proof‐of‐concept and a continuous effort for the discovery of novel GPX4 degraders, we describe the design and synthesis of a series of heterobifunctional molecules, and characterize GDAz‐3 as a potent GPX4 degrader and ferroptosis‐inducing agent (Figure 1E). More importantly, the active molecule demonstrated a higher degradation rate and a wider range of applications than our previously reported HIM‐PROTAC GDCNF‐11.

## 2 Results and Discussion

### 2.1 Design, Synthesis, and Characterization of GDAz‐3 as a Potent GPX4‐Targeting HSP70‐PROTAC

Similar to PROTAC, the novel HSP70‐PROTAC strategy proposed in this study also involves a heterobifunctional molecule composed of three distinct structural moieties: a POI binder, an HSP70 binder, and a suitable linker to connect them. To verify our hypothesis, we assessed whether novel functional GPX4 degraders could be derived from previously reported reversible HSP70 binders. We first analyzed their binding modes based on the previously published cocrystal structure or our molecular docking results. The cocrystal structure of ML162 in complex with GPX4 (PDB ID: 6HKQ; Figure S1A, Supporting Information) revealed that ML162 occupies the flat surface of the GPX4 protein coupled with the formation of three hydrogen bond interactions with residues Gly47, Trp136, and Gln81, as well as a covalent bond interaction to Sec46. Three aryl groups in ML162 were exposed to the solvent region, suggesting that these sites may be suitable for the linker connection. Herein, the solvent‐exposed methoxy in the aryl ring was identified as the optimal site for linker attachment. The HSP70 family consists of multiple members with inducible Hsp70 (also named Hsp72/HSPA1A) and constitutive Hsc70 (a heat shock cognate 70 protein, also named HSPA8) being the two major cytosolic isoforms and well‐studied proteins of this family. Therefore, our primary focus was on these two proteins in this study. Given that Hsp70 and Hsc70 share a high degree of sequence homology and structural similarity (over 85%), we selected Hsp70 (PDB ID: 4IO8) for docking simulations. To the best of our knowledge, there are relatively few reports available on HSP70 inhibitors compared with those on HSP90 inhibitors. Among them, apoptozole (Az) is an imidazole‐based small molecule that inhibits HSP70 by blocking ATPase activity with high affinity for the Hsc70 and Hsp70 (KD values of 0.21 and 0.14 µM, respectively).^[^ ^32^ ^]^ The predicted binding model of apoptozole in complex with Hsp72 revealed that the terminal amide group was oriented toward the solvent‐exposed area and did not form crucial interactions with the residues of Hsp70, which may potentially present a suitable site for conjugation with a GPX4‐targeting ligand, facilitated by a linker (Figure S1B, Supporting Information). Linker rigidity and spatial orientation are crucial for maintaining a proper geometry for efficient ubiquitination, degradation selectivity, and physicochemical properties.^[^ ^33^ ^]^ By changing the type of linker, we synthesized 14 novel GPX4 degraders mediated by the HSP70 molecular chaperone complex (Figure 1E; Scheme S1, Supporting Information).

**Figure 2:**
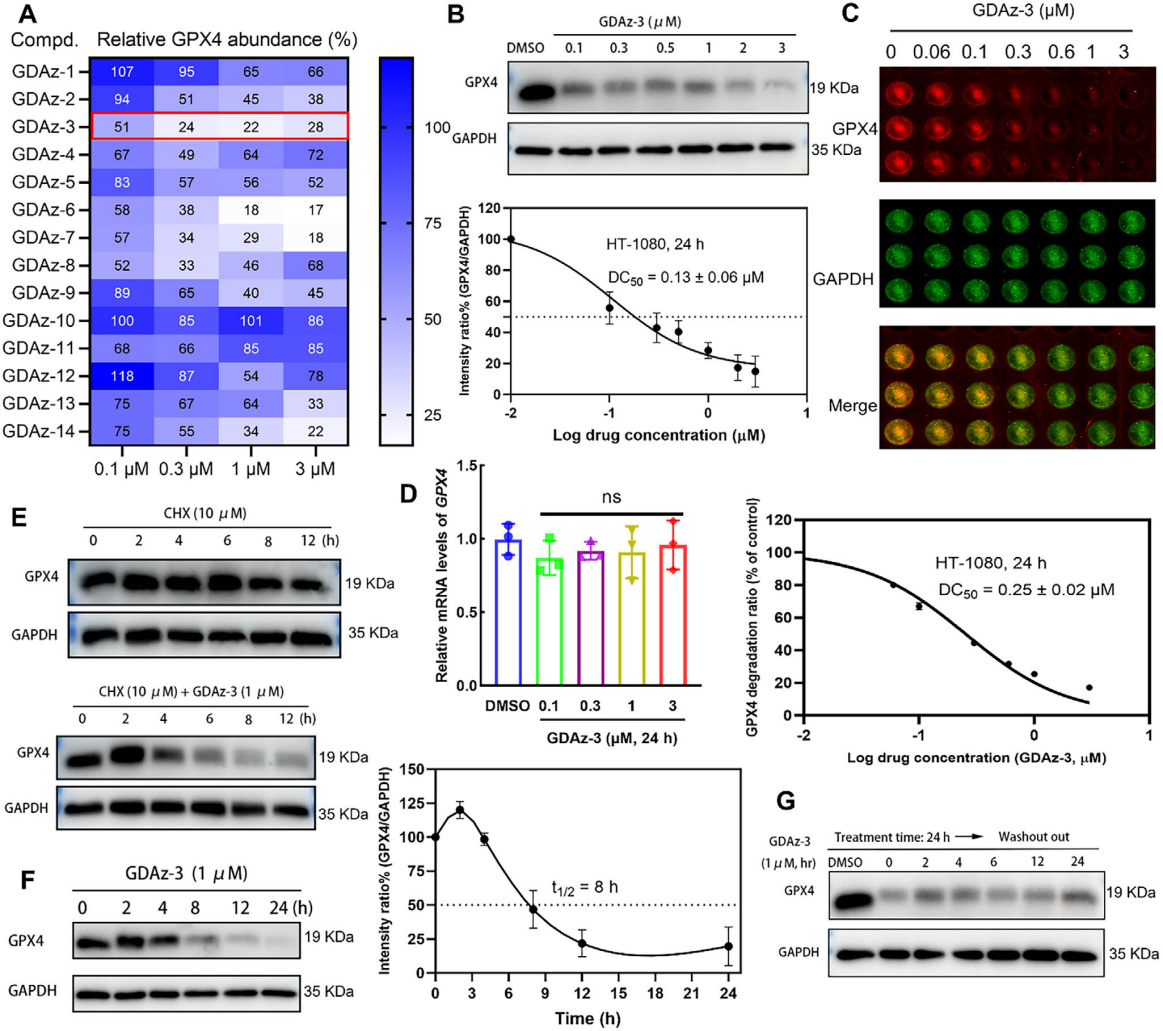
Characterization of GDAz‐3 as a potent GPX4 degrader. A) The preliminary screening of GPX4 degradation effect of the synthesized HSP70‐PROTACs after treatment with different concentrations (0.1, 0.3, 1, and 3 µM) for 24 h in HT1080 cells (calculated using Image J software and the respective DMSO treatment group:100%). B) The representative protein band and the DC_50_ curve of compound GDAz‐3 in degrading GPX4 at various concentrations in HT1080 cells after treatment for 24 h as determined by western blot (n = 3). C) The fluorescence imaging and the DC_50_ curve of compound GDAz‐3 in degrading GPX4 at various concentrations in HT1080 cells after treatment for 24 h as determined by In‐Cell Western. (D) The mRNA levels of *GPX4* were detected by quantitative real‐time PCR assay after treatment with GDAz‐3 for 24 h at the designed concentrations (n = 3). E) The protein bands of compounds CHX or in combination with GDAz‐3 in degrading GPX4 for the designated time. F) The GPX4 protein level was determined at the indicated time‐points after treatment with GDAz‐3 (n = 3). G) Sustained cellular degradative activity induced by GDAz‐3 (1 µM) upon washout in HT1080 cells. Data represent mean ± SD and analyzed by one‐way ANOVA with Dunnett's multiple comparison test (C, compared to vehicle group; ns: not significant).

To evaluate the GPX4 degradation efficiency of the synthesized HSP70‐PROTACs GDAz‐1–14, we first selected ferroptosis‐sensitive HT1080 fibrosarcoma cells (highly‐expressing GPX4) treated with different concentrations (0.1, 0.3, 1, and 3 µM) of these chimeras to determine the changes in the endogenous levels of GPX4 by western blot assay after 24 h treatment. The preliminary screening result revealed that the level of GPX4 was reduced by these HSP70‐PROTACs to varying degrees (**Figures** **2**A, and S2, Supporting Information). This finding underscores the crucial role of the linker in HSP70‐PROTAC‐mediated protein degradation. Chimeras with flexible chains (PEG chains, GDAz‐1–5; and alkyl chains, GDAz‐6–10) generally showed superior GPX4 degradation activity (except GDAz‐10) relative to those with rigid chains (heterocyclic chains, GDAz‐11–14). In the case of alkyl chain derivatives (GDAz‐6–10), elongating the length of the linker generally led to a decreased GPX4 degrading activity. Among the tested compounds, GDAz‐2–7 showed a relatively stronger ability to degrade GPX4 in a dose‐dependent manner, with GDAz‐3 being characterized as the most potent in this series, reducing the GPX4 protein level by ≈50% at a concentration of 0.1 µM. Thus, GDAz‐3 was selected for further evaluation.

Based on a semi‐quantitative study using western blotting, GDAz‐3 exhibited the highest GPX4 degradation efficiency, with a DC_50_ value of 0.13 µM in HT1080 cells after 24 h of treatment (Figure 2B). To further confirm this degradation efficacy, an in‐cell western (ICW) assay, a quantitative immunofluorescence cell‐based technique, was performed to quantify the relative expression level of GPX4 after treatment with GDAz‐3 for 24 h. In line with the western blot result, ICW analysis also revealed the potent GPX4 degradation of GDAz‐3 with a comparable DC_50_ value of 0.25 µM (Figure 2C). Given the promising GPX4 degradation activity of GDAz‐3, we extended our evaluation to additional ferroptosis‐sensitive cell lines, including triple‐negative breast cancer cells (MDA‐MB‐231) and melanoma cells (mouse B16‐F10). The results showed that GDAz‐3 could effectively induce the degradation of GPX4 to varying extents in different cell lines after treatment for 24 h, especially for the B16‐F10 cell line, manifested as the markedly decreased GPX4 level at 3 µM (Figure S3, Supporting Information). Based on the observation that GDAz‐3 was particularly effective in HT1080 cells, we selected this cell line for further degradation mechanism and ferroptosis pharmacodynamics studies.

To confirm whether GPX4 was downregulated by HSP70‐PROTACs at the transcriptional level, we performed a quantitative real‐time PCR assay on GDAz‐3‐treated HT1080 cells at different concentrations (0.1, 0.3, 1, and 3 µM). The results revealed that GDAz‐3 did not significantly affect the level of *GPX4* mRNA at any of the tested concentrations. This suggests that GDAz‐3 most likely directly degrades the GPX4 protein rather than interfering with its transcriptional expression (Figure 2D). Furthermore, the half‐life of GPX4 was determined to be more than 12 h when new protein synthesis was blocked by cycloheximide (CHX, 10 µM), whereas GPX4 protein was obviously reduced by co‐treatment with CHX and GDAz‐3 at 4 h (Figure 2E). These results support that GDAz‐3 decreases the level of GPX4 protein through a direct degradation process rather than by intervention in its expression. Next, we evaluated the kinetics of GDAz‐3 in the induction of GPX4 degradation at 1 µM in HT1080 cells. The result showed that GDAz‐3 obviously reduced GPX4 protein level after 8 h and achieved near‐complete GPX4 depletion at the 24‐h timepoint (Figure 2F), although the protein level of GPX4 in HT1080 cells was observed to be upregulated after treatment with GDAz‐3 for 2 h. As expected, this finding also suggests that the HSP70 interactome‐based PROTAC has a considerably faster GPX4 degradation rate compared to our previously reported HSP90 interactome‐based GPX4 degraders, with active compound GDCNF‐11 initiating GPX4 protein degradation after 12 h.^[^ ^28^ ^]^ We speculated that the increased degradation rate of GPX4 by GDAz‐3 may partly be ascribed to the special structure of the HSP70 chaperone degradation complex. Subsequently, we sought to monitor the sustained cellular degradative activity of GDAz‐3 upon washout. HT1080 cells were treated with 1 µM GDAz‐3 for 24 h and then rinsed with phosphate‐buffered saline (PBS) to remove the excess extracellular compound. A sustained GPX4 degradation effect was still observed, with GPX4 protein maintained at a low level without obvious recovery even 24 h after removal of the degrader (Figure 2G). These findings revealed that the degrader GDAz‐3 had a long‐lasting or post‐dosing effect on the degradation of GPX4, which may partially benefit from the covalent binding of GDAz‐3 to GPX4. On the other hand, this degradation persistence also implies that GDAz‐3 is likely less susceptible to cellular metabolism, as confirmed by the subsequent pharmacokinetic study (t_1/2_ = 10.2 h). To further assess selectivity, we performed proteomics profiling of HT1080 cells with treatment of GDAz‐3 and observed clear degradation of GPX4 (Figure S4, Supporting Information). Overall, these results suggest favorable GPX4 degradation characteristics of GDAz‐3.

### 2.2 Degradation Mechanism of HSP70‐PROTAC

**Figure 3:**
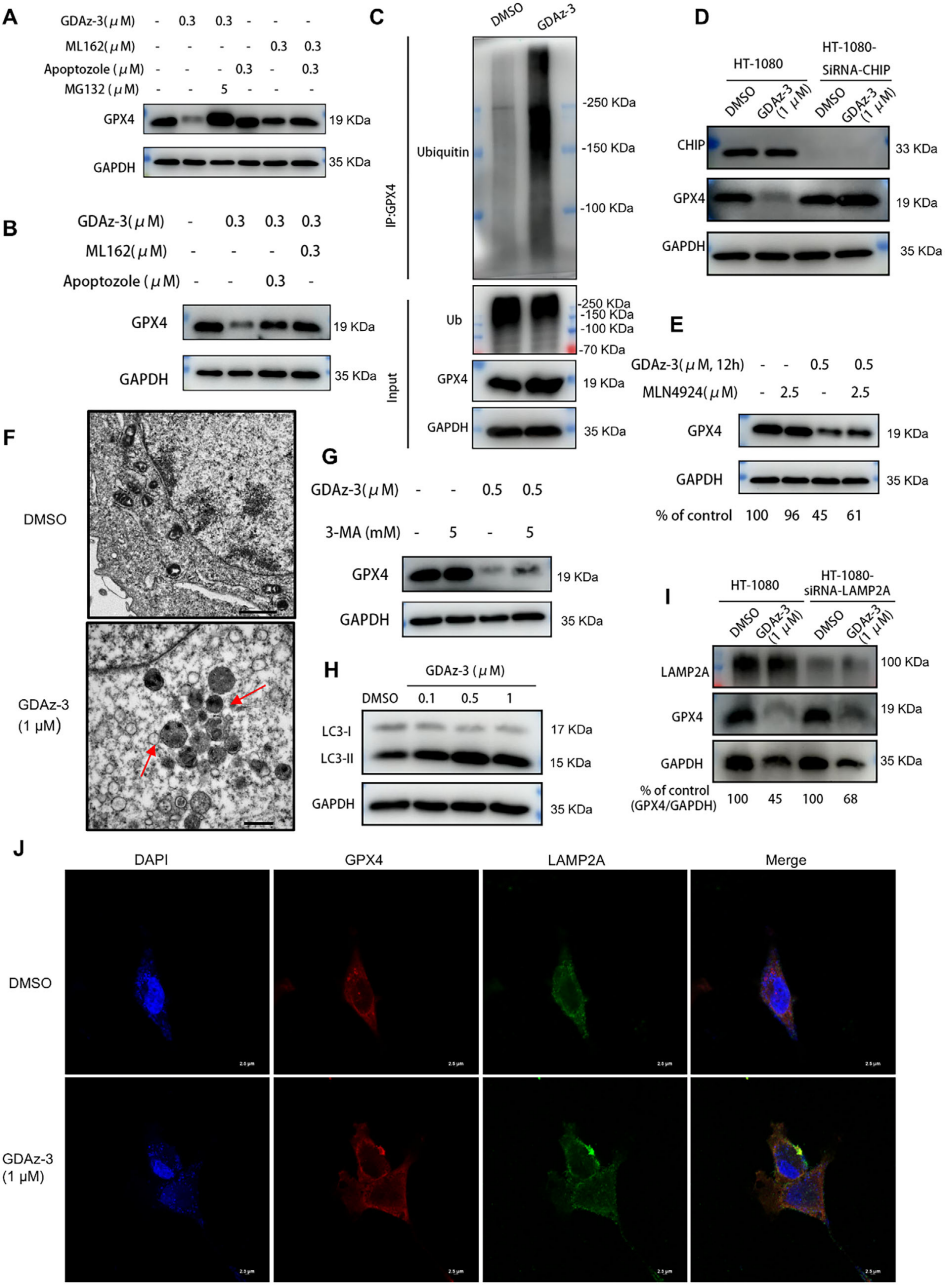
Both UPS and CMA processes were involved in HSP70‐PROTAC‐mediated GPX4 degradation. A,B) Western blot analysis of GPX4 in HT1080 cells after treatment with compounds as indicated for 24 h. C) The ubiquitination assay of GPX4 after treatment with GDAz‐3 (1 µM) for 12 h. D) Western blot analysis of GPX4 in CHIP‐knockdown HT1080 cells after treatment with GDAz‐3 for 24 h. E) Pre‐treatment with MLN4924 (2.5 µM) for 2 h before the addition of GDAz‐3 for 12 h to prevent Cullin‐dependent ubiquitination and GPX4 degradation. F) Transmission electron microscopy images of autophagosomes in HT1080 cells treated with DMSO and GDAz‐3 (1 µM) for 24 h (scale bar = 1 µm). G,H) Western blot analysis of the level of GPX4 in HT1080 cells after treatment with compounds as indicated for 24 h. I) Western blot analysis of GPX4 in LAMP2A‐knockdown HT1080 cells after treatment with GDAz‐3 for 24 h. J) Representative confocal fluorescence images of HT1080 cells treated with GDAz‐3 (1 µM) for 24 h (scale bar = 2.5 µm).

The ubiquitin‐proteasome system (UPS) is one of the primary intracellular pathways for cells to eliminate damaged or unnecessary proteins.^[^ ^34^ ^]^ We attempted to determine whether GDAz‐3‐induced GPX4 degradation is dependent on the UPS. As shown in **Figure** **3**A, GDAz‐3 effectively triggered substantial GPX4 degradation in HT1080 cells at 0.3 µM, whereas this degradation effect was not observed for their respective ligands/inhibitors (ML162 and apoptozole) or a combination of them, suggesting a unique action mechanism of GDAz‐3. Next, we treated HT1080 cells with the proteasome inhibitor MG132 (5 µM) for 2 h before adding GDAz‐3 for 24 h. As expected, the GPX4 degradation effect was completely abrogated, demonstrating that GDAz‐3‐induced GPX4 deletion was attributed to proteasomal degradation. Furthermore, pretreating HT1080 cells with 0.3 µM ML162 (GPX4‐binding competitive ligand) or Apoptozole (HSP70‐binding competitive ligand) successfully rescued GPX4 depletion caused by GDAz‐3 (Figure 3B). This phenomenon results from the competition for the same binding site with ML162 and Apoptozole, indicating that GDAz‐3 can simultaneously bind to HSP70 and GPX4 and mediate their interactions. Ubiquitination is an enzymatic process where ubiquitin proteins attach to substrate proteins, and the primary outcome of ubiquitination is the degradation of the protein through the proteasome. As mentioned above, GPX4 was accumulated after treatment with the proteasome inhibitor MG132, suggesting the occurrence of ubiquitination modification during GDAz‐3‐mediated GPX4 degradation. Therefore, we detected the ubiquitination of GPX4 after GDAz‐3 treatment. As anticipated, GDAz‐3 could obviously increase the ubiquitination of GPX4 (Figure 3C). Similar to HSP90, HSP70 can also recruit multiple E3 ubiquitin ligases for substrate degradation. CHIP (C‐terminus of Hsc70‐interacting protein, STUB1), a member of U‐box‐containing E3 ubiquitin ligase family, is one of the most extensively studied E3 ubiquitin ligases, which can elicit protein ubiquitination degradation upon interacting with HSP70.^[^ ^35^, ^36^ ^]^ To determine whether CHIP was involved in GDAz‐3‐mediated GPX4 ubiquitination, we knocked down the expression of CHIP by small interference RNA (siRNA) and found that GDAz‐3 did not induce any detectable protein degradation of GPX4 after treatment for 24 h (Figure 3D). Proteins of the Cullin‐RING family (e.g., CUL1, CUL2, CUL3, CUL4A, and CUL5) are other well‐studied E3 ubiquitin ligases for polyubiquitination of the target proteins.^[^ ^37^ ^]^ To confirm whether the Cullin‐RING family of E3 ubiquitin ligases is involved in the HSP70‐PROTAC‐mediated degradation, MLN4924, a pan‐cullin inhibitor that prevents cullin activation through inhibition of NAE (neddylation activating enzyme), was selected to validate our hypothesis. The result disclosed that GPX4 degradation caused by GDAz‐3 was partially rescued when HT1080 cells were pretreated with 2.5 µM MLN4924 (Figure 3E). These observations highlight that multiple E3 ubiquitin ligases participate in GPX4 degradation mediated by HSP70‐PROTAC, mainly through CHIP and partly through Cullin‐RING E3 ligases, as the NEDD8 activating enzyme inhibitor MLN4924 only partially rescued GPX4 degradation. Given that to date no CHIP‐based PROTAC is available, this result also implies that the CHIP E3 ubiquitin ligase holds great potential for the development of new protein degradation tools in the future, but lack of effective and selective binding ligands for CHIP precluded further validation in this study.

As mentioned above, HSP70‐PROTAC demonstrates to have more superiority in the degradation of GPX4 when compared to our previously reported HIM‐PROTAC. Beyond the recruitment of multiple E3 ubiquitin ligases, we hypothesize whether there exist other pathways mediating the degradation of HSP70‐PROTAC. Hsc70, a well‐characterized member of the HSP70 family, has been implicated in chaperone‐mediated autophagy (CMA).^[^ ^38^ ^]^ Misfolded and monomeric substrates bound to Hsc70 are preferentially degraded by CMA or UPS. In this study, given that GPX4 harbors a KFERQ‐like motif and GDAz‐3‐mediated binding of GPX4 to Hsc70, we investigated whether, in addition to the UPS pathway, the CMA pathway also contributes to the degradation of GPX4. Transmission electron microscopy (TEM) study revealed that the presence of numerous autophagosome‐like structures in HT1080 cells after treatment with GDAz‐3, but they were seldom observed in the control group, indicating a potential role of GDAz‐3 in triggering ferroptosis via the activation of autophagy (Figure 3F). 3‐Methyladenine (3‐MA), an inhibitor of early‐stage autophagy by blocking the initiating complex in the classical autophagy pathway, was employed to further validate the involvement of autophagy in GDAz‐3‐mediated GPX4 degradation. The result showed that the GDAz‐3‐induced GPX4 degradation effect was partially rescued when used in combination with 3‐methyladenine (3‐MA) (Figure 3G). Furthermore, the expression level of the autophagy marker protein LC3 was also detected. Simultaneously, LC3‐II was increased after treatment with GDAz‐3, especially at concentrations of 0.5 and 1 µM (Figure 3H). Lysosome‐associated membrane protein type 2A (LAMP2A) is a well‐established critical regulator of CMA, acting on the lysosomal membrane as a receptor for substrate proteins of CMA.^[^ ^39^ ^]^ To further confirm that the CMA pathway is involved in GDAz‐3‐mediated GPX4 degradation, we knocked down the level of LAMP2A in HT080 cells and found that the GPX4 degradation effect triggered by GDAz‐3 was slightly weakened compared with that in the corresponding wild‐type cells (Figure 3I). Additionally, the increased co‐localization of GPX4 and LAMP2A following GDAz‐3 treatment, as depicted in Figure 3J, also supported the translocation of GPX4 to the lysosomal surface subsequent to its interaction with heat shock cognate protein 70 (Hsc70) facilitated by GDAz‐3, where the substrate is transported to the lysosomal lumen for degradation through the action of LAMP2A. In general, all these findings disclosed that, in addition to the ubiquitin‐proteasome system degradation mechanism, the autophagy process is also involved in GDAz‐3‐mediated GPX4 degradation. However, it should be noted that in terms of the extent to which inhibitors rescue the degradation of GPX4, the UPS is the main pathway for GDAz‐3 to trigger GPX4 degradation.

**Figure 4:**
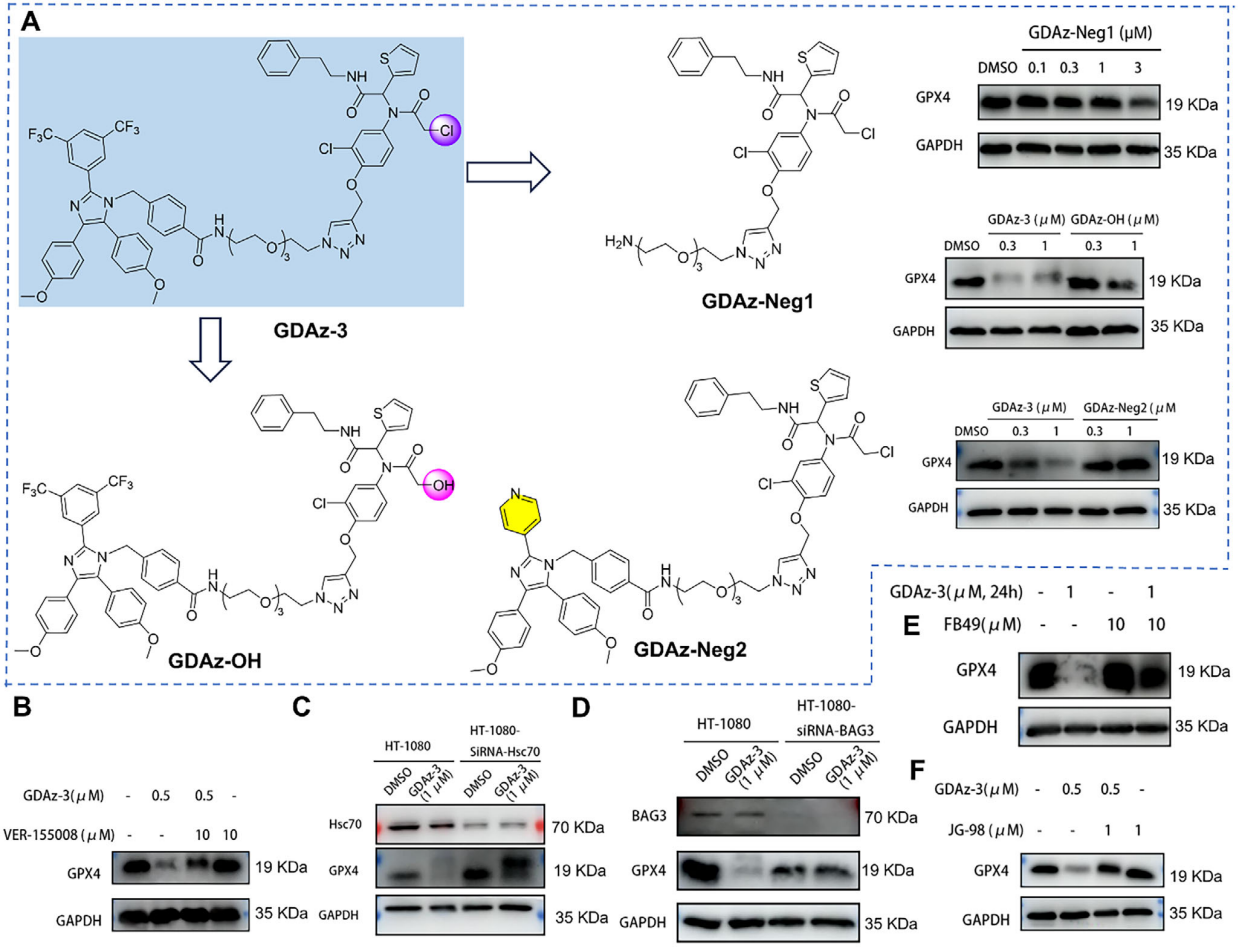
Hsc70‐CHIP‐BAG3 chaperone complex participates in GDAz‐3‐mediated GPX4 degradation. A) Chemical modification of GDAz‐3 and western blot analysis of GPX4 in HT1080 cells after treatment with three negative compounds GDAz‐Neg1, GDAz‐Neg2, and GDAz‐OH (unable to bind with HSP70 or GPX4) for 24 h. B) Western blot analysis of GPX4 in HT1080 cells after treatment with compounds as indicated for 24 h. C) Western blot analysis of GPX4 in Hsc70‐knockdown HT1080 cells after treatment with GDAz‐3 for 24 h. D) Western blot analysis of GPX4 in BAG3‐knockdown HT1080 cells after treatment with GDAz‐3 for 24 h. E,F) Western blot analysis of GPX4 in HT1080 cells after treatment with compounds as indicated for 24 h.

Subsequently, we intended to confirm whether the GPX4 degradation effect triggered by GDAz‐3 was the result of a direct interaction with the HSP70 chaperone complex. We carried out some chemical modifications by removal of the HSP70‐binding moiety in GDAz‐3 (GDAz‐Neg1; Scheme S2, Supporting Information), and replacing the ditrifluoromethylbenzene ring with a pyridine ring in the apoptozole (GDAz‐Neg2, inactive to bind with HSP70, Scheme S3, Supporting Information) to prevent this compound from binding to HSP70, or replacing the chlorine atom on GDAz‐3 with a hydroxyl group (GDAz‐OH, unable to covalently bind to GPX4, Scheme S4, Supporting Information) to prevent this compound from binding to GPX4. As expected, none of the compounds effectively triggered GPX4 degradation at the tested concentrations (**Figure** **4**A). Consistently, co‐treatment with GDAz‐3 and VER‐155008, another potent HSP70 inhibitor, failed to induce GPX4 degradation when HSP70 activity was blocked (Figure 4B). These results indicate that HSP70 is involved in GDAz‐3‐mediated GPX4 degradation, and GDAz‐3 mediates a direct interaction between GPX4 and HSP70.

It is well known that the HSP70 family comprises multiple members. Among them, Hsc70 and Hsp70 are the most abundantly expressed members of this family, and both are involved in proteasomal degradation by interacting with UPS substrates and E3 ubiquitin ligases, thereby facilitating ubiquitination.^[^ ^40^ ^]^ In this study, apoptozole was used to recruit HSP70, but it did not exhibit selectivity for Hsp70 or Hsc70 isoform. To further determine which HSP70 isoform is involved in mediating the GPX4 degradation by HSP70‐PROTAC, we performed knockdown experiments with a siRNA targeting the Hsp70 or Hsc70 isoform. Unexpectedly, the expression level of GPX4 was increased when *Hsp70* or *Hsc70* was knocked down by siRNA. To our surprise, an obvious GPX4 degradation by GDAz‐3 (1 µM) was markedly abrogated when the Hsc70 isoform was knocked down (Figure 4C), while this degradation effect was not affected by the Hsp70 knockdown (Figure S5, Supporting Information). In contrast to our previous report where both HSP90α and hsp90β were found to participate in HIM‐PROTAC‐mediated GPX4 degradation, the current result suggested that Hsc70, rather than all members of the HSP70 family, is involved in GPX4 degradation mediated by the HSP70‐PROTAC. Overall, all these results highlight the great potential of GDAz‐3 binding to the Hsc70 isoform, which may provide a reasonable explanation for the aforementioned finding that GDAz‐3 triggers GPX4 degradation through the UPS and CMA dual pathways.

As co‐chaperones of HSP70, BAG‐family proteins, specifically BAG1 and BAG3, provide a physical connection between the heat shock proteins Hsc70 and the proteasome to facilitate ubiquitin‐proteasome mediated protein degradation.^[^ ^41^ ^]^ To determine which of these proteins participated in the HSP70‐PROTAC‐mediated GPX4 degradation, BAG1 and BAG3 were knocked down by specific siRNAs. The results revealed that the GDAz‐3‐induced GPX4 degradation effect was completely abolished upon BAG3 knockdown (Figure 4D), whereas this effect was not observed in the BAG1‐knockdown group (Figure S6, Supporting Information). Meanwhile, the critical role of BAG3 in mediating GDAz‐3‐induced GPX4 degradation was further corroborated by BAG3 inhibitors. FB49, a BAG3 inhibitor that binds human BAG3 with high affinity,^[^ ^42^ ^]^ can entirely abolish the GPX4 degradation effect induced by GDAz‐3 (Figure 4E). Furthermore, this phenomenon can also be observed after co‐treatment with JG‐98, an allosteric inhibitor of the BAG3‐HSP70 interactome (Figure 4F). Collectively, these results support that the Hsc70‐CHIP‐BAG3 chaperone complex participates in HSP70‐PROTAC‐mediated GPX4 degradation via the UPS process.

**Figure 5:**
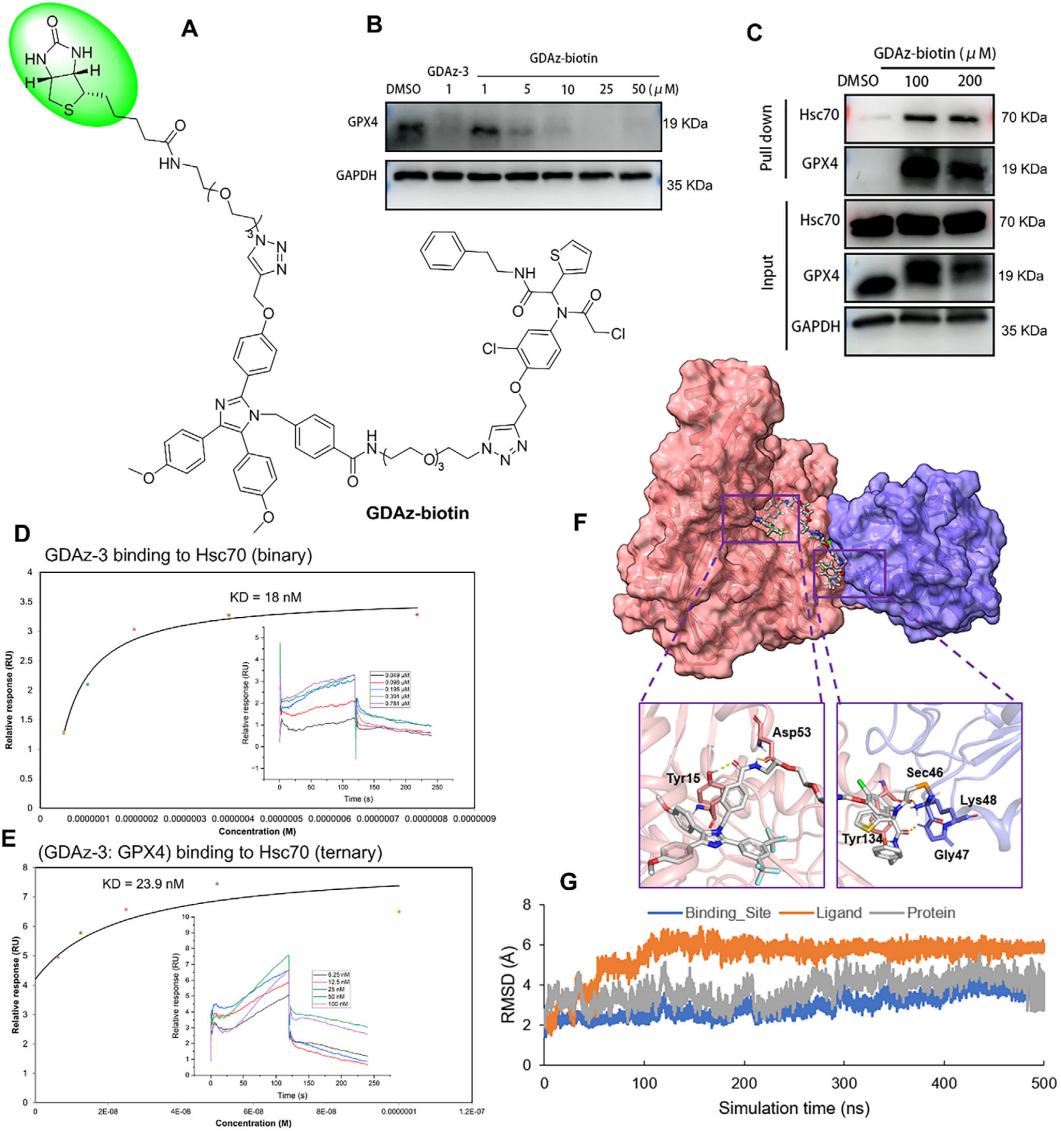
Verification of the ternary complex of Hsc70/GDAz‐3/GPX4. A) Chemical structure of GDAz‐biotin. B) Western blot analysis of GPX4 in HT1080 cells after treatment with GDAz‐biotin for 24 h. C) The interactions between Hsc70 and GPX4 mediated by GDAz‐3 were determined by pull‐down assay. D) SPR analysis of the interaction between immobilized Hsc70 and GDAz‐3. E) Binding affinity of GDAz‐3 to the immobilized Hsc70 protein in the presence of GPX4 protein. F) Computational docking simulation of the ternary complex of Hsc70/GDAz‐3/GPX4 (yellow dashed lines: hydrogen bonds). G) RMSD curve of the ternary structure of Hsc70/GDAz‐3/GPX4 in 500 ns MD simulations.

Indeed, the previous chemical modifications have preliminarily revealed that GDAz‐3 mediated the direct interaction between HSP70 and GPX4. However, whether a ternary complex (GPX4/HSP70‐PROTAC/HSP70) was formed during the process of GPX4 degradation induced by HSP70‐PROTAC needs to be further explored, since the formation of the ternary complex is essential for both the CMA and UPS pathways. Initially, as shown in the above‐mentioned Figure 3B, pretreatment of HT1080 cells with 0.3 µM ML162, a competitive ligand that binds to GPX4, or apoptozole, a competitive ligand that binds to HSP70, successfully rescued GPX4 depletion caused by GDAz‐3. This suggests that HSP70‐PROTAC simultaneously engages in competitive binding with GPX4 and the Hsc70 isoform. To further prove the formation of GPX4/HSP70‐PROTAC/HSP70 ternary complex, we synthesized a biotin‐labeled GDAz‐3 derivative by connecting a biotin molecule to the solvent‐exposed trifluoromethyl‐containing benzene ring of apoptozole (Figure S1B, Supporting Information) via a long linker (**Figure** **5**A, GDAz‐biotin, for the convenience of synthesis, we removed the two trifluoromethyl groups on apoptozole, Scheme S5, Supporting Information). Although the GPX4 degradation activity of the probe GDAz‐biotin was slightly lower than that of GDAz‐3, it still showed considerable GPX4 degradation activity when the concentration was over 5 µM (Figure 5B). Consistent with our prior expectation, the in vitro pull‐down experiment demonstrated that GDAz‐3 could capture Hsc70 and GPX4 simultaneously at all the selected concentrations (Figure 5C). Next, we further evaluated the ability of GDAz‐3 to induce ternary complex formation, as measured by surface plasmon resonance (SPR) assay. We first measured the binding affinities of the compound GDAz‐3‐protein (Hsc70 or GPX4) and the Hsc70‐GPX4 binary complex. GDAz‐3 was found to strongly bind to Hsc70 with a KD value of 18 nM (Figure 5D), but to GPX4 with a KD value of 1.17 µM (Figure S7A, Supporting Information). We speculated that the moderate binding affinity between GDAz‐3 and GPX4 may be attributed to the flat and shallow active pocket of GPX4, which is unfavorable for the interaction with the relatively large small‐molecule GDAz‐3. From another perspective, this moderate affinity can still maintain the potent GPX4 degradation efficiency by GDAz‐3, which may also suggest an event‐driven mechanism of action consistent with PROTAC. GPX4 also exhibited a strong interaction with Hsc70, characterized by an affinity of 100 nM (Figure S7B, Supporting Information), while this interaction was further enhanced to fourfold in the presence of GDAz‐3 (KD = 23.9 nM, Figure 5E), suggesting GDAz‐3‐mediated strong ternary‐complex formation, albeit still displaying a negative cooperativity (α = 0.75). Taken together, these results confirmed the formation of a ternary complex between Hsc70, GPX4, and GDAz‐3, which facilitated the recruitment of E3 ubiquitin ligases from Hsc70 to GPX4, leading to the subsequent ubiquitin transfer and proteasomal degradation or activation of autophagy to degrade GPX4.

To gain a better understanding and visualization of the ternary complex geometry for efficient GPX4 degradation, a computational docking simulation of the ternary complex of Hsc70/GDAz‐3/GPX4 was performed. As shown in Figure 5F, the predicted binding mode showed that at one end containing the ML162 fragment, the chloroacetamide moiety formed a covalent bond with selenocysteine residue Sec46, two hydrogen bonds with Gly47 and Lys48 of GPX4, and another hydrogen bond with Tyr134 of Hsc70. Compared with the crystal structure of GPX4‐ML162 (PDB ID: 6HKQ), there was a slight conformational change in Hsc70 after the protein‐protein interaction. At the opposite end, the apoptotic moiety formed two hydrogen bonds with the residues Tyr15 and Asp53 of Hsc70. Meanwhile, π‐π stacking interactions can also be observed between Tyr15 and the phenyl ring of apoptozole. To further confirm the stability of the ternary complex, a molecular dynamics (MD) simulation was performed and the root mean square deviations (RMSD) of ligand (shown in orange color), binding site (shown in blue color), and protein (shown in grey color) against simulation time were displayed in Figure 5G and Figure S8, Supporting Information. After a 500 ns MD simulation, the overall conformational change of compound GDAz‐3 in the pockets of both proteins fell within an acceptable range, indicating the formation of a stable ternary complex. Overall, these critical molecular interactions may contribute to stabilizing the binding of GDAz‐3 with Hsc70 and GPX4, facilitating the orientation of GDAz‐3 to adjust and stabilize the ternary complex.

### 2.3 Degradation of GPX4 by GDAz‐3 to Trigger Ferroptosis

**Figure 6:**
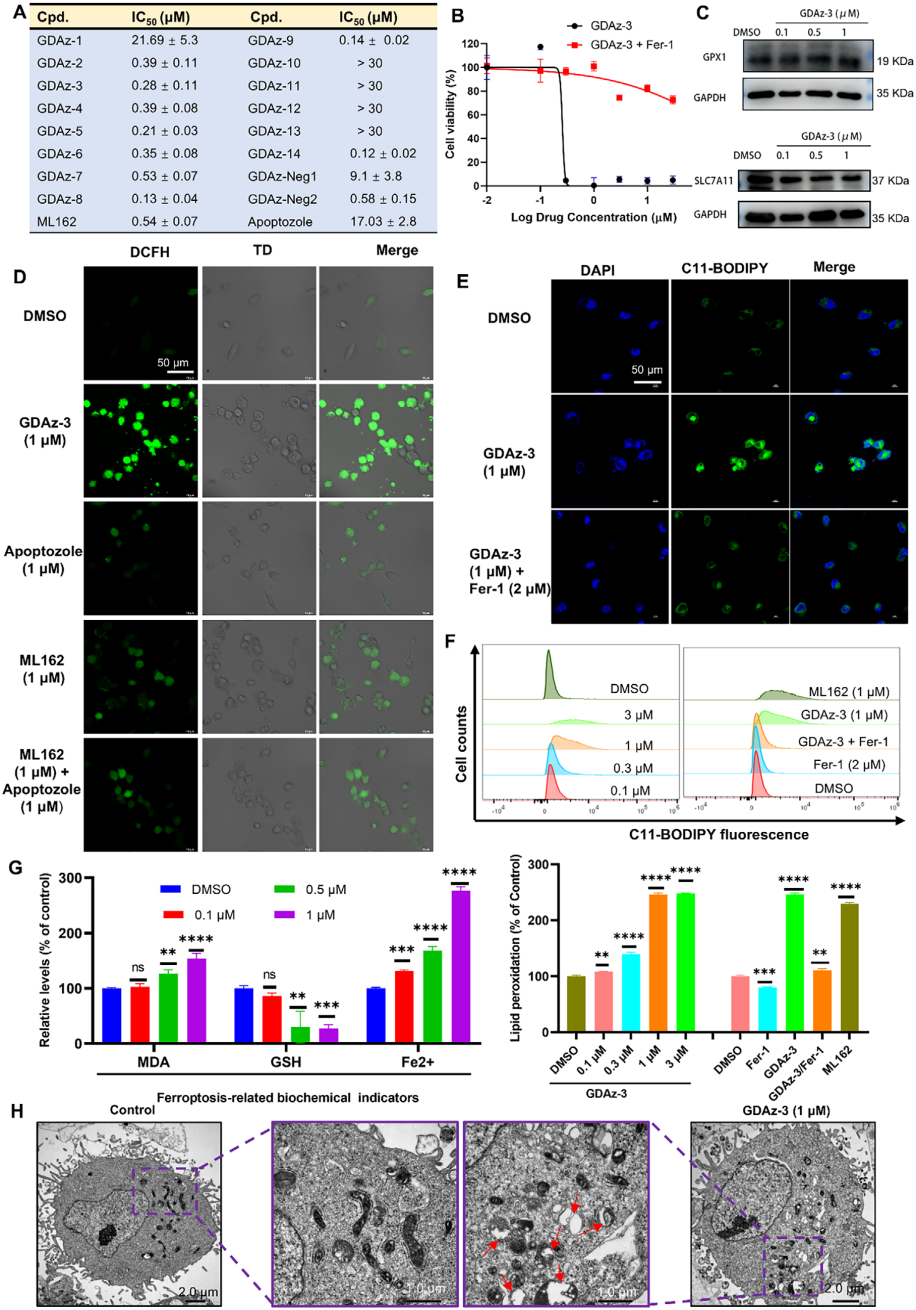
Degradation of GPX4 by GDAz‐3 triggers ferroptosis. A) Cytotoxicity of all the synthesized HSP70‐PROTACs including positive control drugs against HT1080 cells (data are presented as mean ± SD). B) Ferroptosis selectivity of GDAz‐3. HT1080 cells were incubated with the tested compounds or in combination with 2 µM Fer‐1 for 48 h. C) Western blot analysis of GPX1 and SLC7A11 in HT1080 cells after treatment with GDAz‐3 for 24 h. D) Confocal laser scanning microscopy imaging of intracellular total ROS upon treatment with the specified agents at 1 µM for 24 h. E) Confocal laser scanning microscopy imaging of intracellular LPO upon treatments with the specified agents at the designated concentration for 24 h. F) Flow cytometry analysis for intracellular LPO of HT1080 cells treated with the specified agents at the designed concentrations at the designated concentration for 12 h (n =3). G) Determination of the content of intracellular MDA, GSH, and Fe^2+^ after treatment with various concentrations of GDAz‐3 for 24 h (n =3). H) Transmission electron microscopy images of HT1080 cells treated with DMSO, and GDAz‐3 (1 µM) for 24 h; red arrow: decreased or absent mitochondrial crests, or broken outer membrane. Data represent mean ± SD. *p < 0.05, **p <0.01, ***p < 0.001, ****p < 0.0001 compared to control group by one‐way ANOVA with Dunnett's multiple comparisons; ns: not significant.

To determine whether GPX4 degradation following treatment with HSP70‐PROTACs triggers cancer cell ferroptosis, we initially assessed the viability of HT1080 cells using the CCK‐8 assay after treatment with various concentrations of the tested compounds for 48 h. GPX4 inhibitor ML162, HSP70 inhibitor apoptozole, and two GPX4 non‐degradable HSP70‐PROTAC molecules, GDAz‐Neg1 and GDAz‐Neg2, were selected as controls. As depicted in **Figure** **6**A, although there are slight differences in their GPX4 degradation activities, anti‐proliferation activities of GDAz‐2–9 were comparable with IC_50_ values ranging from 0.13 to 0.53 µM, which were superior to that of the GPX4 inhibitor ML162. Given that the linker is crucial for complex formation and induction of GPX4 degradation, we hypothesized that the relatively strong anti‐proliferation activity exhibited by some compounds (e.g., GDAz‐4 and GDAz‐5, whose degradation activity was moderate or decreased at higher concentrations) may partly stem from their inhibitory activity against GPX4 or the dual inhibition of GPX4 and HSP70. Notably, we found that GDAz‐14, a compound with significant GPX4 degradation activity, exhibited the most potent anti‐proliferation activity in this series, with an IC_50_ value of 0.12 µM. This remarkable activity might partly be ascribed to the high cell permeability conferred by the piperazine ring. On the contrary, compounds GDAz‐10–13 showed negligible activity against the growth of the tumor cell line with IC_50_ values over 30 µM. Notably, apoptozole, an inhibitor of HSP70, showed moderate growth‐inhibitory activity against HT1080 cells (IC_50_ = 17.03 µM). In contrast, although GDAz‐Neg2 is an inactive GPX4 degrader, it still showed antiproliferative activity comparable to that of the GPX4 inhibitor ML162. We speculated that the potent anti‐proliferative activity of GDAz‐Neg2 may be attributed to the GPX4 inhibition. In general, most of them showed good agreement between cell growth inhibition and GPX4 degradation. Similarly, the promising activity of GDAz‐3 in the GPX4 protein degradation assay was translated well into cellular activity, as evidenced by its growth‐inhibitory potency against HT1080 cells, with an IC_50_ value of 0.28 µM. However, after co‐incubation with 2 µM of ferroptosis inhibitor Fer‐1 (a peroxide scavenger widely used for inhibiting ferroptosis), the cytotoxicity of GDAz‐3 was significantly decreased with an IC_50_ value of over 30 µM, suggesting an extremely high ferroptosis selectivity of GDAz‐3 (Figure 6B). Based on this observation, it can be concluded that the HSP70 interactome‐based PROTAC GDAz‐3 showed high superiority over the GPX4 inhibitor ML162 (IC_50_ = 0.76 µM, ferroptosis selectivity index about threefold) and our previously reported the most potent HSP90 interactome‐based GPX4 degrader GDCNF‐11 (IC_50_ = 0.79 µM, ferroptosis selectivity index about 45‐fold) in terms of antiproliferative activity and ferroptosis selectivity under the same experimental conditions.^[^ ^28^ ^]^ Furthermore, we confirmed that such high ferroptosis selectivity stems from the degradation of GPX4 because GDAz‐3 did not show a significant effect on other members of the GPX family like GPX1, and the other ferroptosis‐related proteins such as SLC7A11, indicating high targeting specificity and low side effects of GDAz‐3 (Figure 6C).

Degradation of GPX4 leads to the up‐regulation of lethal lipid peroxidation (LPO) accompanied by the generation of plentiful reactive oxygen species (ROS) for executing ferroptosis. Excessive generation of ROS is a prerequisite for ferroptosis; therefore we evaluated the intracellular level of ROS using 2′,7′‐dichlorofluorescin diacetate (DCFH‐DA) as a fluorescent probe. Confocal laser scanning microscopy (CLSM) showed that the level of intracellular ROS was enhanced after treatment with 1 µM GDAz‐3, which was higher than that observed after treatment with ML162, Apoptozole, and their combination (Figure 6D). We continued to evaluate the level of intracellular LPO in HT1080 cells treated with GDAz‐3 using C11‐BODIPY^581/591^, an oxidation‐sensitive and LPO‐specific fluorescent probe. As expected, intracellular LPO was enhanced following treatment with 1 µM GDAz‐3 in HT1080 cells, as determined by CLSM imaging; however, the level of intracellular LPO was significantly decreased when co‐incubated with 2 µM ferroptosis inhibitor Fer‐1 (Figure 6E). To further quantitatively evaluate the level of LPO, flow cytometry analysis revealed that the fluorescence intensity of C11‐BODIPY^581/591^ arising from lipid peroxidation was markedly enhanced in a concentration‐dependent manner when the cells were treated with GDAz‐3, with superior potency to that of ML162 at 1 µM (Figure 6F). Similarly, the fluorescence intensity was significantly decreased when the cells were co‐incubated with GDAz‐3 and Fer‐1. All the above‐mentioned phenomena provided strong evidence that GDAz‐3 possesses a high selectivity for ferroptosis.

As a metabolite of LPO, the content of malondialdehyde (MDA) is an extensively utilized indicator for the determination of LPO. As illustrated in Figure 6G, the content of MDA in HT1080 cells was concentration‐dependently increased after incubation with GDAz‐3. Moreover, GSH is a coenzyme essential for GPX4, and the level of intracellular GSH influences the enzymatic activity of GPX4. Therefore, we also evaluated the effect of GDAz‐3 on intracellular GSH levels. The level of intracellular GSH was significantly decreased after incubation with 0.5 and 1 µM GDAz‐3, suggesting a dual mechanism of action for GDAz‐3‐triggered ferroptosis in cells, involving direct degradation of GPX4 and exhaustion of GSH. As mentioned earlier, the accumulation of LPO is a hallmark of ferroptosis, which can be triggered by an increase in ferrous ions (Fe^2+^). Thus, we determined the level of intracellular Fe^2+^ with an iron colorimetric assay kit, and the results showed that the content of Fe^2+^ in HT1080 cells was increased in a concentration‐dependent manner by incubation with GDAz‐3. Besides, ferroptosis has been shown to induce distinct alterations in the morphology of intracellular mitochondria. The cell ferroptosis induced by 1 µM of GDAz‐3 in HT1080 cells was investigated by transmission electron microscopy. As illustrated in Figure 6H, the cells displayed typical morphological changes associated with ferroptosis, including ruptured mitochondria, mitochondrial swelling, decreased or absent mitochondrial crests, and broken outer membrane. Overall, the observed elevation in the intracellular levels of LPO, ROS, MDA, and Fe^2^⁺, accompanied by a reduction in GSH levels and alterations in mitochondrial morphology after treatment with GDAz‐3, strongly suggest the occurrence of ferroptosis.

### 2.4 In Vivo Pharmacokinetic and Ferroptosis‐Driven Antitumor Efficacy of GDAz‐3 in the HT1080 Xenograft Mouse Model

**Figure 7:**
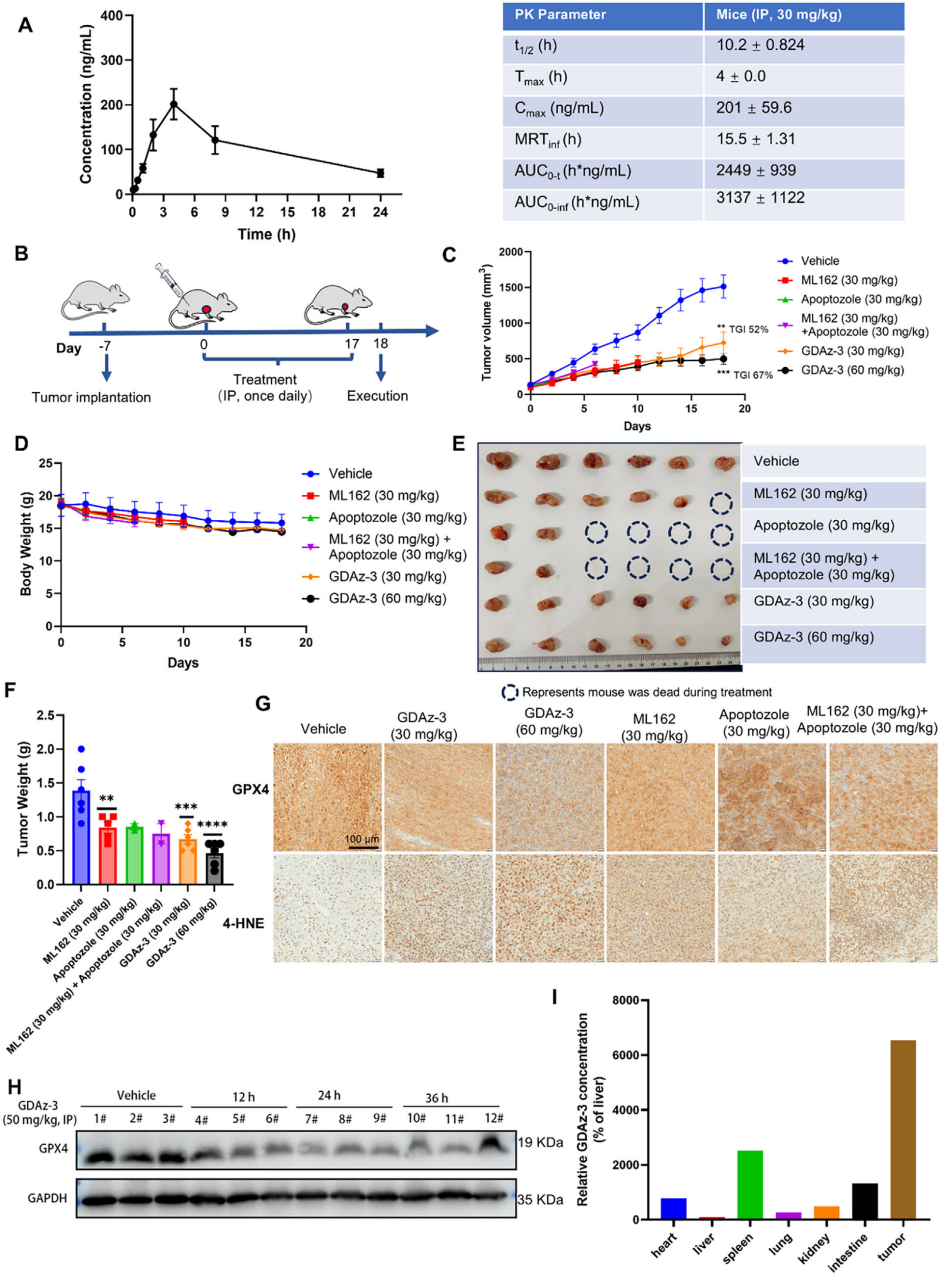
PK and antitumor evaluation of GDAz‐3 in vivo. A) Concentration‐time curve and PK parameters of GDAz‐3 (n = 3). B) Schematics of in vivo antitumor efficacy study of GDAz‐3 (n = 6 per group). C) Tumor growth curves of HT1080 tumor‐bearing mice subjected to diverse treatments (the data were not presented once the mouse died). D) Changes in the body weights of mice over the treatment period (the data were not presented once the mouse died). (E) Image of the subcutaneous tumor of xenografts in different groups of nude mice; F) Comparison of tumor weight in mice from different groups; G) representative immunohistochemistry staining analysis of GPX4 and 4‐HNE from different groups. H) Pharmacodynamic analysis of GPX4 protein in the HT1080 xenograft tumors in nude mice. Mice‐bearing xenograft tumors were treated with a single administration of either vehicle control or GDAz‐3 at 50 mg kg^−1^ via intraperitoneal injection, and the mice were sacrificed at the indicated time points. Tumor tissues were harvested for western blotting analysis. I) Quantitative analysis of GDAz‐3 concentration in tumor, and other major organ tissues of the HT1080 xenograft tumors in nude mice after intraperitoneal injection at 12 h (n = 1). Data represent mean ± SEM. *p < 0.05, **p <0.01, ***p < 0.001, ****p < 0.0001 compared to vehicle group by one‐way ANOVA with Dunnett's multiple comparisons (B and E).

To evaluate the in vivo pharmacokinetic (PK) profile of the GDAz‐3 and explore its therapeutic potential, a single intraperitoneal (IP) injection (30 mg kg^−1^) was conducted in male ICR mice. Plasma samples were collected at predetermined time points and analyzed using LC‐MS/MS. As shown in **Figure** **7**A, the half‐life (t_1/2_) of GDAz‐3 is 10.2 h, with a maximum concentration (C_max_) of 201 ng mL^−1^ and an area under the curve (AUC) of 3137 h*ng mL^−1^. These results collectively highlight the favorable pharmacokinetic properties of GDAz‐3 upon IP delivery, supporting the further pharmacodynamic study.

Motivated by the aforementioned in vitro findings, we subsequently sought to evaluate the in vivo antitumor efficacy of GDAz‐3 using an HT1080 xenograft tumor mouse model. The tumor‐bearing mice were randomly divided into six groups, minimizing weight and tumor size differences among the groups (n = 6 in each group). The treatment group mice were administered once daily by intraperitoneal injection (IP) with GDAz‐3 at a dosage of 30 or 60 mg kg^−1^, while the control group animals were treated with the corresponding solvent, ML162, and apoptozole at a dosage of 30 mg kg^−1^. Additionally, we set up a drug combination group to confirm the superiority of GDAz‐3. After treatment for 17 days, animals were humanely euthanized and the tumors were excised (Figure 7B). Compared with vehicle‐treated animals, GDAz‐3 could significantly suppress tumor growth in a dose‐dependent manner with TGI (tumor growth inhibition) values of 52% (30 mg kg^−1^) and 67% (60 mg kg^−1^) (Figure 7C). The morphology and weight of the tumors in each group also demonstrated the advantages of GDAz‐3 in suppressing tumors in vivo (Figure 7E,F). Moreover, treatment with GDAz‐3 at all the tested dosages did not obviously reduce the body weight of the mice, while ML162, apoptozole, and their combination therapy at a dosage of merely 30 mg kg^−1^ had the potential to induce serious toxic effects such as death during the treatment (Figure 7D,E). Notably, although apoptozole showed moderate cytotoxicity at the cellular level against HT1080 cell line, it exhibited significant toxicity in vivo in our study through an as‐yet‐unknown mechanism. The advantage of the safety of GDAz‐3 was also determined by the hematoxylin and eosin (H&E) staining. We did not observe significant toxic changes in the major organs (heart, liver, spleen, lung, and kidney) in the GDAz‐3‐treated group, while an obvious inflammatory reaction was further identified by H&E staining of the spleen for the drug combination therapy group (Figure S9, Supporting Information). In general, compared with the single inhibitor or their combination, the in vivo antitumor study confirmed the efficacy and safety of GDAz‐3.

In addition, the tumor samples from the six treatment groups were randomly collected and probed for the levels of GPX4, and 4‐HNE (4‐hydroxynonenal, a lipid peroxidation marker) by immunohistochemistry (IHC) staining (Figure 7G). As anticipated, the groups treated with GDAz‐3 demonstrated a dose‐dependent reduction in GPX4 levels and an increase in 4‐HNE levels compared to the vehicle group, which was consistent with our previous in vitro GPX4 degradation result. Simultaneously, the western blot analysis of tumor tissue indicated that a single intraperitoneal injection of GDAz‐3 (50 mg kg^−1^) could also reduce the level of GPX4 protein over the vehicle control at the 12, 24, and 36 h time points in the HT1080 tumor tissues (Figure 7H). To further confirm whether HSP70‐PROTAC possessed the selectivity for tumor tissues in the HT1080‐xenograft model, we determined the relative drug distribution in major organs by liquid chromatography‐tandem mass spectrometry after intraperitoneal injection of GDAz‐3 (50 mg kg^−1^) for 12 h. In line with our hypothesis, the result indicated that the concentration of GDAz‐3 in the tumor tissue was significantly higher than that in other tissues, especially the heart, liver, kidney, and lung tissues, suggesting the tumor‐targeted pharmacokinetics of HSP70‐PROTAC in the HT1080‐xenograft model (Figure 7I). In general, compared with other treatment groups, GDAz‐3 not only exhibited promising therapeutic efficiency in combating tumors in vivo but also alleviated the toxicity when the GPX4 inhibitor ML162 was covalently connected to the HSP70 inhibitor apoptozole. Collectively, the HSP70‐PROTAC GDAz‐3 has been confirmed as an effective ferroptosis‐inducing agent, which can degrade GPX4 both in vitro and in vivo via UPS and CMA dual processes.

## 3 Potential Applicability of Harnessing HSP70 for Protein Target Degradation

**Figure 8:**
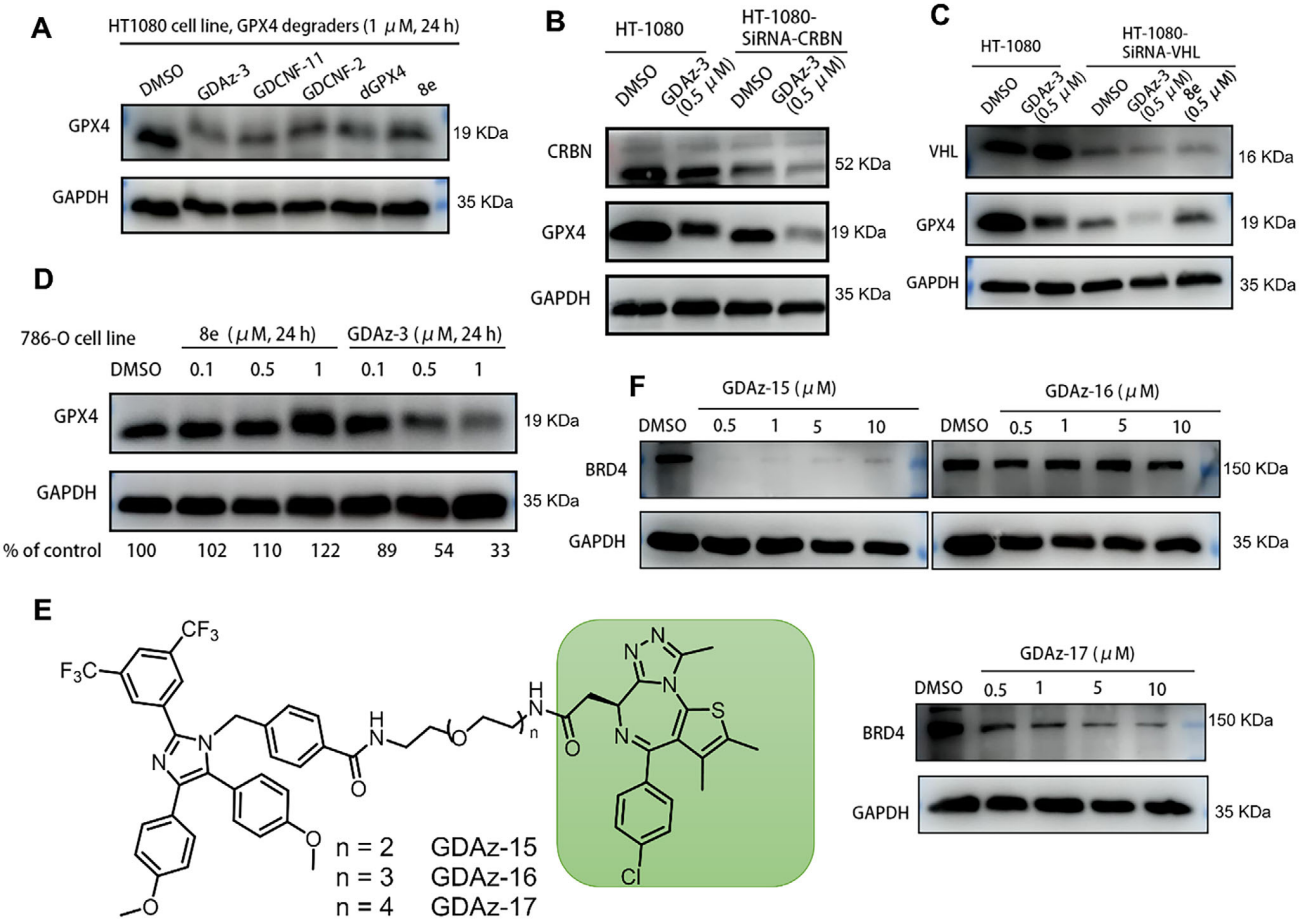
Potential Applicability of HSP70‐PROTAC. A) Western blot analysis of GPX4 in HT1080 cells after treatment with reported GPX4 degraders (1 µM) for 24 h. B,C) Western blot analysis of GPX4 in CRBN‐ or VHL‐knockdown HT1080 cells after treatment with GDAz‐3 and 8e for 24 h. D) Western blot analysis of GPX4 in 786‐O cells after treatment with GDAz‐3 and 8e for 24 h. E) Chemical structures of BRD4‐targeting HSP70‐PROTAC GDAz‐15–17. F) Western blot analysis of their protein degradation activity of BRD4 in the MOLT‐4 cell line after treatment for 48 h.

As previously mentioned, drug resistance and limited application scope are the significant challenges of traditional PROTAC, due partly to CRBN/VHL mutations or low expression. In this study, we investigated whether the developed HSP70‐PROTAC could address these challenges. Specifically, we examined the developed HSP70‐PROTAC in scenarios where VHL‐ or CRBN‐based PROTACs are ineffective due to intrinsic or acquired resistance. Several previously reported representative GPX4‐targeting degraders, including dGPX4 (CRBN‐based PROTAC, Figure 1A), 8e (VHL‐based PROTAC, Figure 1B), and GDCNF‐11/2 (HSP90‐based HIM‐PROTAC, Figure 1C, the structure of the GDCNF‐11 derivative GDCNF‐2 was not shown) were selected as control agents.^[^ ^18^, ^23^, ^28^ ^]^ As shown in **Figure** **8**A, GDAz‐3 showed a comparable GPX4 degradation efficiency to those reported GPX4 degraders after 24‐hr incubation in HT1080 cells at a concentration of 1 µM. Notably, treatment with 0.5 µM of HSP70‐PROTAC GDAz‐3 promoted the degradation of GPX4 both in HT1080 wild‐type and CRBN‐ or VHL‐knockdown cells (Figure 8B,C), while this degradation effect of 8e on VHL‐knockdown HT1080 cells was not observed after treatment for 24 h, which also validates the successful knockdown of VHL. The clear cell renal cell carcinoma (ccRCC) 786‐O cell line is a well‐established cancer cell model characterized by an intrinsic deficiency in VHL expression.^[^ ^43^, ^44^, ^45^ ^]^ We also confirmed this characteristic through evaluating the VHL expression levels in several randomly selected cancer cell lines (Figure S10A, Supporting Information). We further selected 786‐O cells as a model to confirm whether HSP70‐PROTAC could be used in such cases as an alternative to VHL‐based degraders, because VHL‐based PROTACs cannot be used to treat kidney cancer with a mutation or deletion in the VHL gene. To our delight, GDAz‐3 still showed an obvious GPX4 degradation at 0.5 µM in 786‐O cells following a 24 h treatment (Figure 8D), whereas neither the PROTAC dGPX4 (up to 1 µM, higher concentrations prevent reliable detection due to the internal reference protein degradations) nor our previously reported active HIM‐PROTAC GDCNF‐11 (up to 10 µM) induced any degradation effect even at higher concentrations (Figure S10B,C, Supporting Information), suggesting the superiority of HSP70‐hijacking degraders over the corresponding HSP90‐hijacking degraders. These results validated that HSP70‐PROTAC can be considered as an alternative TPD strategy for the application of PROTACs in biological contexts where CRBN or VHL is absent or altered due to mutations.

To expand beyond the GPX4 degradation, we next sought to investigate the potential of the HSP70 complex to facilitate the degradation of additional proteins. We selected oncoprotein bromodomain containing 4 (BRD4), a degradable protein that not only has been widely used as a model substrate for development of new technologies in the TPD field, but also serves as a validated therapeutic target for multiple cancers. We synthesized three heterobifunctional compounds (GDAz‐15–17) by conjugating the HSP70 binding moiety apoptozole to the well‐established BRD4 inhibitor (+)‐JQ‐1 (a potent, specific, and reversible BRD4 inhibitor extensively applied in TPD) via PEG linkers of varying lengths (Figure 8E; Scheme S6, Supporting Information). T lymphoblast MOLT‐4 cell line with BRD4 overexpression was selected for biological evaluation. As shown in Figure 8F, two of them, GDAz‐15 and GDAz‐17 could effectively trigger BRD4 degradation after 48 h of treatment in MOLT‐4 cells, especially for GDAz‐15 with nearly thorough BRD4 degradation at a concentration of 0.5 µM. Overall, these data suggest that the development of HSP70‐hijacking PROTACs for other targets is feasible, while similar to the well‐established PROTAC technology, an appropriate linker is particularly crucial for the formation of the ternary complex and the initiation of protein degradation.

Targeted protein degradation (TPD) represents an emerging therapeutic approach for eliminating disease‐causing proteins with aberrant expression. Current research on drugs and tools for targeted protein degradation is extensive, primarily focusing on proteolysis‐targeting chimeras (PROTACs) and molecular glues.^[^ ^13^, ^46^, ^47^ ^]^ These PROTACs and molecular glues predominantly rely on a single E3 ubiquitin ligase, such as CRBN, VHL, or other E3 ubiquitin ligases being developed for targeted protein degradation. However, relatively limited research is available on the utilization of molecular chaperone complexes to mediate protein degradation. Molecular chaperones like HSP90 can recruit multiple ubiquitin ligases rather than a specific E3 ligase to facilitate targeted protein degradation that can be utilized to develop novel protein degradation tools, as demonstrated in our previous study and other studies as well.^[^ ^28^, ^48^, ^49^ ^]^ Given that each E3 ligase has a dedicated type of substrate and that the use of a single E3 ligase may lead to drug resistance,^[^ ^50^ ^]^ molecular chaperone‐mediated heteromolecules could expand the application scope for substrate protein degradation. This study introduces HSP70‐PROTAC, a novel TPD tool that harnesses the HSP70 molecular chaperone (Hsc70 isoform) complex to effectively degrade GPX4 in both conventional tumor cell lines and cell lines with CRBN/VHL knockdown or deficiency. Thus, HSP70‐PROTAC demonstrates a notable advantage over traditional PROTACs and our previously reported HSP90 interactome‐mediated proteolysis targeting chimera (HIM‐PROTAC).^[^ ^28^ ^]^ Furthermore, compared with our previously reported active GPX4 degraders mediated by the HSP90 chaperone complex, the herein reported GDAz‐3 exhibited enhanced GPX4 degradation rate, prolonged degradation effect, and improved ferroptosis selectivity. Additionally, the high expression of Hsc70 in tumor tissues, combined with the presence of multiple E3 enzymes in the molecular chaperone complex, presumes that HSP70‐PROTAC may possess enhanced tumor selectivity and drug resistance‐prevention ability. However, it should be noted that the current research still faces some limitations, including 1) similar to CRBN, VHL or other E3, the HSP70‐based degrader is also sensitive to the linker for targeting POI, and a suitable linker is critical for POI degradation; 2) like the conventional PROTAC, the HSP70‐PROTAC also exhibits cellular‐dependent degradation of target proteins, as evidenced by our observation that these bifunctional molecules did not potently degrade our designated POI including GPX4, and BRD4 across all the selected cell lines during our experimental process, but the underlying mechanism whether related to the expression level of Hsc70 complex requires further investigation; 3) given a restricted range of cell lines and targets employed in this study, the generalizability of HSP70‐PROTAC remains to be further verified across a broader spectrum of cell lines and targets. To clearly clarify these potential limitations, our study focused on utilizing HSP70‐PROTAC for the degradation of more endogenous proteins like anaplastic lymphoma kinase (ALK) is still in progress. In general, the chemically induced proximity between the HSP70 complex and a protein of interest (POI) represents a novel and feasible targeted protein degradation strategy.

## 4 Conclusion

TPD technology has garnered much attention for therapeutic intervention. Herein, we report the first proof‐of‐concept study of HSP70‐PROTAC‐based protein degraders, which specifically target and eliminate endogenous GPX4 to trigger cancer cell ferroptosis. By altering the type of linker, we successfully developed a heterobifunctional small molecule GDAz‐3 that effectively and selectively degrades GPX4 in HT1080 cells with a DC_50_ value of 0.13 µM. Meanwhile, this compound not only potently inhibited the growth of HT1080 cells in vitro with IC_50_ values in the low micromolar range, but also showed an effective tumor growth inhibition effect in HT1080 tumor‐bearing nude mice without obvious toxicity. Further mechanistic studies demonstrated that GDAz‐3 reduced the level of GPX4 in an Hsc70 isoform‐dependent manner, with both the UPS and CMA implicated in the GPX4 degradation process. Furthermore, the HSP70‐PROTAC strategy could also be extended to degrade BRD4 when tagged with the BRD4 inhibitor (+)‐JQ‐1. As an emerging TPD paradigm, HSP70‐PROTAC facilitates the ubiquitination and degradation of POI by recruiting the overexpressed and hyperactivated Hsc70 chaperone complex in tumor tissues, rather than a single E3 ligase (VHL or CRBN). As a result, GDAz‐3‐mediated GPX4 degradation can also be observed with comparable efficiency in CRBN/VHL‐knockdown cells and cells intrinsically lacking VHL expression. Consequently, HSP70‐PROTAC provides a promising alternative to traditional PROTAC development, including our previously reported HIM‐PROTAC. In summary, HSP70‐PROTAC provides fresh insights into drug discovery, which may also act as a supplement to existing targeted protein degradation technology, particularly in providing a viable option to overcome intrinsic or acquired resistance to PROTAC molecules.

## 5 Experimental Section

### Synthesis of Compounds

The chemical synthesis and analytical data of the target compounds were described in the Supporting Information.

### Cell Culture

Fibrosarcoma HT1080 cells, mouse melanoma cells B16‐F10, and human breast cancer cells MDA‐MB‐231 were cultured in DMEM supplemented with 10% fetal bovine serum (FBS), while T lymphoblast cell line MOLT‐4 was grown in RPMI 1640 supplemented with 10% fetal bovine serum (FBS). All the cells were cultured in a humidified incubator (37 °C in an atmosphere of 5% CO_2_).

### Cell Viability Assay

The compounds under investigation were initially dissolved in dimethyl sulfoxide (DMSO) to generate stock solutions with a concentration of 10 mM. HT1080 cells or other tested cells were seeded in 96‐well plates at a density of 3000–5000 cells well^−1^ and cultured at 37 °C. Following a 24‐h incubation period, the tested compounds were added to the wells at various concentrations (the DMSO stock solutions of all the tested compounds were diluted with culture medium to obtain the designated concentration containing a final concentration of DMSO less than 0.5% (v/v)). The cells were then cultured for an additional 48 or 72 h. Cell Counting Kit 8 (CCK‐8) assay was conducted to examine the effects of the tested compounds on cell viability, and the absorbance was measured at a wavelength of 450 nm. The IC_50_ values were determined utilizing GraphPad Prism software through nonlinear regression analysis. All experiments were independently repeated at least three times.

### siRNA Transfection

Human Hsp70, Hsc70, BAG1, CHIP, CRBN, and VHL were knocked down using siRNA transfection. In brief, HT1080 cells were plated in 6‐well dishes at a density of 4 × 10^5^ cells per well and allowed to incubate overnight. When the cells reached 70% confluence, transfection was performed by treating the cells with a transfection mixture composed of 100 pmol siRNA and 7.5 µL of Lipofectamine 2000 Transfection Reagent (Invitrogen Co.) in 0.5 mL of serum‐free DMEM. Following a 6‐h incubation period, the cells were replenished with fresh culture medium containing 10% FBS for further culture for 72 h, and the efficacy of siRNA was confirmed by western blot assay. The specific sequences used in this experiment were shown in Table S1, Supporting Information.

### Western Blot Assay

Cells were seeded at a density of 4 × 10^5^ cells well^−1^ in 6‐well plates and allowed to incubate overnight. Cells were incubated with the indicated treatments. Thereafter, the cells were collected and lysed with RIPA buffer containing serine protease and phosphatase inhibitors for 30 min on ice. Proteins were quantified by using a BCA protein assay kit (Beyotime). Subsequently, SDS‐PAGE sample loading buffer was added, and the mixture was heated in a 100 °C metal bath for 10 min to denature the proteins. Protein samples from each group were separated by SDS‐PAGE gel and transferred to a PVDF membrane. After blocking with 5% skim milk for 1 h at room temperature, primary antibodies (GPX4: abcam (#ab125066, 1:1000); Hsp70: abcam (#ab2787, 1:1000); CHIP: HUABIO (#ST7108, 1:1000); UpingBio Technology Co., Ltd. (BRD4, #YP‐Ab‐18034, 1:1000; BAG‐1, #YP‐Ab‐00575, 1:1000); GAPDH: Proteintech (#60004‐1‐Ig, 1:50 000)) were used and incubated at 4 °C overnight on a rotary shaker. After washing the membranes with TBST buffer five times, the membranes were incubated with a second antibody (Cell Signaling Technology, #7074S, 1:1000 and #7076S, 1:1000) at room temperature for 2 h. Ultimately, the membrane was washed five times, and the antibody‐protein complexes were visualized using an ECL luminescence reagent (Biosharp, Shanghai, China). The relative intensities of the bands were analyzed by ImageJ and normalized to a loading control.

### In‐Cell Western

HT1080 cells were seeded into 96‐well plates at a density of 1 × 10⁴ cells per well and cultured overnight. After 24‐h treatment with the drugs, the culture medium was removed, and 150 µL of 4% paraformaldehyde was added to each well to fix the cells at 4 °C for 20 min. The paraformaldehyde was removed, and 150 µL of 0.2% Triton X‐100 was added to each well. The cells were permeabilized by shaking at room temperature for 30 min. After removing the Triton X‐100, 50 µL of 1% BSA buffer was added to each well, and shaken at room temperature for 90 min. The blocking buffer was discarded, and 50 µL of primary antibodies (GPX4 & GAPDH; diluted at ratios ranging from 1:50 to 1:500) was added to each well. The plates were shaken overnight at 4 °C, and the primary antibodies were recovered, followed by the addition of 150 µL PBST to each well. The plates were shaken at room temperature for 5 min and washed five times. Subsequently, 50 µL of fluorescent secondary antibodies (Goat anti‐rabbit IRDye 680 (1:200; LI‐COR #926‐32221); Goat anti‐mouse IRDye 800CW (1:800, LI‐COR #926‐32210)) were added to each well. The plates were wrapped with tin foil to protect them from light and shaken at room temperature for 60 min. The secondary antibodies were recovered, and 150 µL of PBST was added to each well. The plates were shaken at room temperature for 5 min and washed five times. Then, the cells were washed with PBS for an additional 5 min. The PBS was discarded, and the plates were read using a Typhoon biomolecular imager (Cytiva, GPX4: wavelength 680 nm; GAPDH: 800 nm).

### Quantitative Proteomics Analysis

The proteomics data were generated by Shanghai Bioprofile Co., Ltd. After treatment with compound GDAz‐3 (2 µM) or DMSO for 18 h, the samples were collected. LC‐MS/MS analysis was conducted using an Orbitrap Astral zoom mass spectrometer paired with a Vanquish Neo UHPLC system (Thermo Fisher Scientific). Peptides from each sample were loaded into a column (50 cm Low‐Load µPAC Neo HPLC Column, Thermo Scientific) at a flow rate of 2.2 µL min^−1^. The reversed‐phase high‐performance liquid chromatography (RP‐HPLC) mobile phases consisted of solvent A (0.1% formic acid in water) and solvent B (0.1% formic acid in 80% acetonitrile). Peptide elution was performed over 4.8 min with a linear gradient of buffer B at a flow rate 1.25 µL min^−1^. The linear gradient was programmed as follows: 0–0.1 min, an increase from 4% to 6% solvent B; 0.1–1.1 min, from 6% to 12% buffer B; 1.1–4.3 min, from 12% to 25% buffer B; 4.3–6.1 min, from 25% to 45% buffer B; 6.1–6.5 min, from 45% to 99% buffer B; 6.5–8 min, buffer B maintained at 99%. The eluted peptides were subsequently analyzed on a Orbitrap Astral mass spectrometer. The DIA method consisted of a full scan m/z range of 380–980 at resolution 240 000 with AGC target of 500% and 5 ms injection time. The DIA MS/MS scans were acquired by Astral from 150 to 2000 m/z with 2 m/z isolation window and with AGC target of 500% and 3 ms injection time. The normalized collision energy was set 25 and the cycle time was 0.6 s. Full MS scan spectra were recorded in profile mode, whereas DIA scan spectra were collected in centroid mode.

### Intracellular LPO Measurement

The level of LPO was determined by flow cytometry using the C11‐BODIPY^581/591^ dye. In short, HT1080 cells were incubated in 6‐well plates at a density of 5 × 10^4^ cells per well for 24 h. After that, the culture medium was substituted with a fresh medium containing GDAz‐3, ML162, or a mixture of GDAz‐3 and Fer‐1 at the specified concentrations. Following a 12‐h incubation period, HT1080 cells were stained with C11‐BODIPY^581/591^ dye for 10 min. Finally, the cell samples were collected, washed twice with phosphate‐buffered saline (PBS), and then analyzed by flow cytometry.

### Intracellular ROS Determination

To assess the total intracellular levels of reactive oxygen species (ROS), a DCFH‐DA (Dichloro‐dihydro‐fluorescein diacetate) assay kit was utilized. Briefly, HT1080 cells were seeded into confocal laser culture dishes and allowed to adhere for 24 h. Afterward, the cells were treated with GDAz‐3 at a concentration of 1 µM for 24 h. Subsequently, a DCFH‐DA probe was added to the cells and they were incubated for 20 min. After washing twice with PBS, the cells were visualized and imaged using a confocal laser scanning microscopy (CLSM).

### Immunofluorescence Staining

HT1080 cells were seeded at 70% confluence in a confocal laser culture dish and incubated for 24 h. Subsequently, the cells were treated with GDAz‐3 at a concentration of 1 µM for 12 h. Following treatment, the culture medium was removed, and the cells were washed two to three times with PBS before being fixed with 4% paraformaldehyde (in PBS) at room temperature for 15 min. Post‐fixation, the cells underwent three washes with PBS and then were permeabilized with 0.1% Triton X‐100 solution for 10 min at room temperature. Then, the cells were washed with PBS three times and blocked with 5% BSA for 60 min at room temperature. After blocking, the cells were incubated overnight at 4 °C with the specified primary antibodies (GPX4 at 1:50 dilution and LAMP2A at 1:100 dilution). Following primary antibody incubation, the cells were washed with PBS three times and then exposed to the appropriate combinations of Alexa Fluor (488 or 680)‐conjugated anti‐rabbit or anti‐mouse secondary antibodies for 1 h in the dark at room temperature. After incubation, the cells were washed with PBS three times. Nuclei were stained with 4′,6‐diamidino‐2‐phenylindole (DAPI) and incubated at room temperature for 10 min. After washing with PBS three times, the cells were visualized and imaged using a confocal laser scanning microscopy (A1 RHD25, Nikon).

### Cell Morphology Analysis

HT1080 cells were seeded in 10 cm culture dishes at a density of 2 × 10^6^ and allowed to incubate overnight. Subsequently, the cells were treated with GDAz‐3 (1 µM), while DMSO was used as the control group. After a 24‐h incubation period, the cells were harvested and washed twice with cold phosphate‐buffered saline (PBS). The cells were then centrifuged at 4000 rpm for 5 min. Following centrifugation, the cells were fixed with 2.5% glutaraldehyde. Then, the cells were stained with 1% osmic acid for 1 h at 4 °C. After staining, the cells were rinsed and dehydrated using a gradient of ethanol solutions (30%, 50%, 70%, 90%–100%). The dehydrated cells were then embedded in Epon. The cellular slices were stained with 2% uranyl acetate and 1% lead citrate, and the morphological features were imaged by transmission electron microscope (TEM).

### Real‐Time qRT‐PCR

Real‐time qRT‐PCR was used to determine the mRNA expression level of *GPX4* gene in HT1080 cells. In brief, HT1080 cells were seeded in 6‐well plates at a density of 4.5 × 10^5^ cells per well and allowed to adhere overnight. Subsequently, the cells were exposed to different concentrations of the test compound GDAz‐3 for 24 h. Total RNA from the various cell samples was extracted using an RNA‐Quick Purification Kit (#RC112, Vazyme, Nanjing, China) according to the manufacturer's instructions. The concentrations of the isolated RNA samples were determined using NanoDrop. A total of 2 µg RNA was used to synthesize cDNA using Fast‐All‐in‐One RT Kit (#RT001, ES Science, Shanghai) following the manufacturer's guidelines. Real‐time PCR was performed using 2xSuper SYBR Green QPCR Master Mix (#QP002, ES Science, Shanghai) and determined using a CFX96 PCR instrument with the following cycling conditions: 95 °C for 5 min, 95 °C for 10 s, and 60 °C for 30 s, 40 cycles. The data were analyzed by Bio‐Rad CFX Manager Software version 3.1. The primers used in this experiment were as follows:

GPX4: Forward‐ACAAGAACGGCTGCGTGGTGAA;

Reverse‐GCCACACACTTGTGGAGCTAGA.

GAPDH: Forward‐ACAACTTTGGTATCGTGGAAGG;

Reverse‐GCCATCACGCCACAGTTTC.

### In Vitro Ubiquitination Assay

HT1080 cells were seeded in 15 cm culture dishes and allowed to adhere overnight. Then, the cells were treated with either 1 µM GDAz‐3 or an equivalent volume of DMSO for 12 h. Next, the proteasome inhibitor MG‐132 (5 µM) was added, and the cells were further incubated for 6 h. Once the incubation was completed, the cells were harvested and subjected to lysis using an IP lysis solution (#P0013J, Beyotime, Shanghai, China) to extract proteins. Then, the cell lysates were incubated with the GPX4 antibody (Proteintech, #67763‐1‐Ig, 1:100) overnight at 4 °C. Subsequently, protein A/G‐magnetic beads (#P2179, Beyotime, Shanghai, China) were added, and the mixture was incubated at 4 °C for 2 h on a rotator. After incubation, the beads were washed four times with immunoprecipitation buffer and then boiled in 1× loading buffer. The protein samples were then subjected to SDS‐PAGE analysis to evaluate the interactions between the target proteins. Finally, western blotting was used to detect proteins of interest in the co‐immunoprecipitation products. The ubiquitin assay was carried out under denaturing conditions.

### Probe Pull‐Down Assay Streptavidin Agarose

Pull‐down experiments were performed using the GDAz‐3 probe. HT1080 cells were collected and lysed in RIPA buffer containing protease inhibitor MG132. GDAz‐biotin was added at varying concentrations and incubated for 3 h at room temperature. Subsequently, a fivefold volume excess of pre‐chilled methanol was added and incubated at −80 °C for 30 min to precipitate the protein. The mixture was then centrifuged at 14 000×g for 15 min, and the precipitated proteins were dissolved and incubated overnight with streptavidin agarose beads (Beyotime, P2159) at 4 °C. Then, the streptavidin agarose beads were washed three times with PBST buffer, and the bead‐bound proteins were collected and detected by western blot.

### Surface Plasmonic Resonance Assay

The SPR data was generated by our Shared Instrumentation Core Facility at the Hangzhou Institute of Medicine (HIM), Chinese Academy of Sciences. The in vitro binding affinity of compound GDAz‐3 to recombinant proteins (Hsc70, MedChemExpress, #HY‐P73915A; GPX4, Cayman, Item No. 26 906) was evaluated using a Biacore 8K system (Cytiva (GE)) with the method of LMW multi‐cycle kinetics/affinity. Pipelines and chips were flushed with PBS (pH 7.4) running buffer, and then, human Hsc70 was immobilized on a CM5 sensor chip. Analysis was performed at a flow rate of 30 µL min^−1^ at 25 °C with the injection of incremental concentrations of compounds dissolved in buffer solution (the final concentration of DMSO was 5%). For ternary‐complex binding measurements, Hsc70 was immobilized on a CM5 sensor chip, and compound GDAz‐3 at a concentration of 1 µM was preincubated with GPX4. The binding and dissociation durations were recorded as response unit values for 120 and 120 s in succession, respectively. Finally, the data were analyzed by Biacore evaluation software with the method of LMW multi‐cycle kinetics/affinity.

### Molecular Docking and Molecular Dynamics Simulation Procedures

To construct the ternary complexes of Hsc70‐GDAz‐3‐GPX4, a binary complex of Hsc70‐GPX4 was first modelled. The crystal structure of GPX4‐ML162 (PDB entry: 6HKQ) was downloaded from RCSB (http://www.rcsb.org/). The complex of Hsc70‐apoptozole was obtained by docking simulation with the software of AutoDock Vina based on the crystal structure of the published Hsc70 (PDB code: 3FZM) from RCSB, and the suitable ligand pose was chosen for docking with GPX4. Multiple residues at the entrance of the protein Hsc70 (R261, T265, E268, R342, N364, R36, N35, D53, and N57) and GPX4 (W136, G79, Q81, and Q45) were randomly selected as the indication of active binding sites, and constrained protein‐protein docking simulations were performed by using ZDOCK. Ten candidate binding conformations were reported and downloaded from the ZDOCK server. By analysis of their conformations, we selected a suitable GPX4‐Hsc70 complex for further study. Next, the linker was artificially attached to ML162 and apoptozole for constructing the initial ternary complexes, and the induced fit refine method was used to optimize the initial pose.

To assess the stability of the binding model, molecular dynamics (MD) simulations were conducted utilizing the Amber20 software package. The FF19SB and gaff2 force fields were used to parameterize the proteins, and the small molecule GDAz‐3, respectively. The complex was solvated in a periodic box of TIP3P water molecules and periodic boundary conditions were applied in all directions. Periodic boundary conditions (PBC) were employed to avoid edge effects in MD simulations. Any excess charge of the complex was neutralized by adding sodium or chloride ions. The long‐range electrostatic interactions were calculated by the particle mesh Ewald method. Energy minimization was performed to clear poor contacts, followed by 500 ps NVT and 500 ps NPT equilibrations. Finally, a 500 ns MD production simulation was performed with a 2 fs time step at constant temperature (300 K) and pressure (1 atm).

### Determination of Reduced Glutathione

HT1080 cells were seeded into 10‐cm‐diameter culture dishes at a density of 2 × 10⁶ cells overnight and then exposed to GDAz‐3 (0.1, 0.5, and 1 µM). Followed by 24‐h incubation, the culture medium was discarded, and the cells were digested with trypsin. The cells were collected and washed with PBS, and threefold of protein removal reagent S was added. Two freeze‐thaw cycles were performed using liquid nitrogen and a 37 °C water bath, followed by a 5‐min ice bath. The samples were centrifuged at 10 000 × g for 10 min at 4 °C. The supernatant was collected and measured according to the instructions of the Total Glutathione Assay Kit (Beyotime Biotechnology, S0052) using a kinetic assay method.

### Determination of MDA

HT1080 cells were seeded into 10‐cm diameter culture dishes at a density of 2 × 10⁶ cells overnight and then exposed to GDAz‐3 (0.1, 0.5, and 1 µM). The cells were cultured for another 24 h, and the culture medium was discarded, washed with PBS, and lysed using IP lysis buffer (Servicebio, G2038). After lysis, the samples were centrifuged at 12 000 × g for 15 min at 4 °C. The supernatant was collected, and the MDA content was determined according to the instructions of the Lipid Peroxidation MDA Assay Kit (Beyotime Biotechnology, S00131S).

### Determination of Iron (Fe^2^⁺) Intensity

HT1080 cells were seeded into 10‐cm culture dishes at a density of 2 × 10⁶ cells overnight, and various concentrations of GDAz‐3 (0.1, 0.5, and 1 µM) were added. The cells were digested and collected after treatment with drugs for 24 h. The cells were detected according to the instructions of the Cell Ferrous Iron (Fe^2^⁺) Fluorometric Assay Kit (E‐BC‐F101, Elabscience Biotechnology Co., Ltd.). In brief, the cells were washed twice with Reagent 1 working solution in the kit, and then Reagent 2 working solution was added (1 mL per 10⁶ cells). The cells were incubated at 37 °C in the dark for 30–60 min. The samples were centrifuged at 300 × g for 5 min, and the supernatant was discarded. The cells were washed two to three times with Reagent 1 working solution. After resuspending the cells in 0.2–0.5 mL of Reagent 1 working solution, the samples were detected using a fluorescence microplate reader (excitation wavelength: 542 nm, emission wavelength: 575 nm).

### Quantitative Analysis of the Relative Drug Biodistribution In Vivo

GDAz‐3 was intravenously injected into an HT1080‐bearing xenograft tumor mouse model at 50 mg kg^−1^. After 12 h of treatment, samples of tumor tissue and major organs, including the heart, liver, spleen, lung, kidney, and intestine, were collected. After weighing, these samples were processed into homogenates, and 500 µL of cold methanol was added. After thorough mixing, the supernatant was generated by centrifugation (4 °C, 12 000 rpm, 15 min), and then analyzed by liquid chromatography‐tandem mass spectrometry (Agilent 6495C Triple Quadrupole LC/MS). In brief, a volume of 1 µL from each sample was extracted and injected into a Waters Acquity Premmier BEH C18 (2.1 × 50 mm, 1.7 µm) column. The solvent delivery module was used to deliver an HPLC gradient of 20–95% B over 7 min (A: 0.1% formic acid in water, B: acetonitrile) at a flow rate of 0.3 mL min^−1^. Mass spectrometric detection was carried out using an Agilent LCMS‐6495C quadrupole mass spectrometer equipped with an electrospray ionization (ESI) interface (positive ionization mode). The instrument was scanned within the m/z range of 100–1500. The mass selective detector was operated in multiple reaction monitoring (MRM) mode to achieve optimal selectivity and sensitivity. The quantification was performed based on peak area. Data acquisition and processing were conducted using the Agilent LC‐MS solution software for the LCMS‐6495C system.

### Pharmacokinetic Study

The tested compound GDAz‐3 was IP administered to ICR mice (male, n = 3 per time‐point). Blood samples were collected at 0.0833, 0.25, 0.5, 1, 2, 4, 8, and 24 h after IP administration (30 mg kg^−1^) and then centrifuged at 6800 g for 6 min at 4 °C to obtain plasma, which were stored at −80 °C until analysis The compound concentrations in plasma were determined by liquid chromatography‐tandem mass spectrometry (API5000 LC‐MS/MS).

### In Vivo Mouse Anti‑Tumor Activity Study

The experimental procedures of the animal experiments were permitted by the Hangzhou Institute of Medicine (HIM), Chinese Academy of Sciences (AP2024‐07‐0010). Male BALB/c nude mice (6 weeks old) were housed under pathogen‐free conditions, maintained at a constant room temperature, and fed standard rodent chow and water. An HT1080 fibrosarcoma xenograft model in nude mice was established through subcutaneous transplantation. When the tumors emerged, the mice were randomly divided into six groups with six mice in each group: GDAz‐3‐treated at 30 or 60 mg kg^−1^ group and control groups, including the vehicle group, and each of the two inhibitor‐treated groups (ML162 and apoptozole) at 30 mg kg^−1^, and their combinations. The mice were intraperitoneally (IP) injected daily according to their body weight. Body weight and tumor size were measured every other day, and the tumor volume and body weight were recorded every other day after drug treatment. After treatment, the animals were euthanized by cervical dislocation, and the tumor bulks were peeled off and weighed. The organ tissues (tumor, heart, liver, spleen, lung, and kidney) were harvested and fixed with 4% paraformaldehyde for hematoxylin‐eosin (H&E) and immunohistochemistry (IHC) staining. In addition, the expression of GPX4 in tumor samples was analyzed in vehicle‐ and GDAz‐3‐treated groups by western blot assay.

### Statistical Analysis

For all data shown, stated values represent the mean ± S.D. or S.E.M. of at least three independent experiments (unless otherwise stated). Significant differences between the control and experimental groups were analyzed using Student's *t*‐test or one‐way ANOVA analysis. GraphPad Prism 9.0 software (GraphPad Software, USA) was used for data statistics and calculations. (****) *p* < 0.0001, (***) *p* < 0.001, (**) *p* < 0.01, and (*) *p* < 0.05 were considered statistically significant differences.

## Conflict of Interest

The authors declare no conflict of interest.

## Author Contributions

J.D., Y.L., H.L., and Z.W. contributed equally to this work. J.D., Y.L., and M.C. performed the medicinal chemistry. Y.L., H.L., and Z.W. performed the cellular and biochemical assays. S.W., J. X., H.Y., G.P., K.M., Z.‐S.C., and Y.L. provided guidance, expertise or specialized reagents. Y.W. performed the ICW assay. J.D., F.T., X.M., and J.‐J.Q. conceived the experiments and wrote the manuscript with input from all authors.

## Supporting information



Supporting Information

## Data Availability

The data that support the findings of this study are available in the supplementary material of this article.
