# Supplementary material for: HSP70 Interactome‐Mediated Proteolysis Targeting Chimera (HSP70‐PROTAC) for Ferroptosis‐Driven Cancer Treatment
Source: Adv Sci (Weinh). 2025 Dec 12;13(12):e13655. doi: 10.1002/advs.202513655 (PMC12948223; doi:10.1002/advs.202513655)

Supporting Information

**HSP70 Interactome-Mediated Proteolysis Targeting Chimera (HSP70-PROTAC) for Ferroptosis-Driven Cancer Treatment**

**Table of Contents**

*Supplementary Tables 2*

*Supplementary Figures 3*

*Materials and Methods for Chemistry 13*

**Supplementary Tables**

**Supplementary Table 1**. The specific sequences used in this study

| Gene name | Sequence |
| --- | --- |
| CRBN | Senseseq: 5'-CAAGCCAUAUUGGAUGGAAAUTT-3'  Antiseq: 5'-AUUUCCAUCCAAUAUGGCUUGTT-3' |
| Hsc70 | Senseseq: 5'-GCCCGAUUUGAAGAACUGAAUTT-3'  Antiseq: 5'-AUUCAGUUCUUCAAAUCGGGCTT-3' |
| Hsp70 | Senseseq: 5'-CGACCUGAACAAGAGCAUCAATT-3'  Antiseq: 5'-UUGAUGCUCUUGUUCAGGUCGTT-3' |
| CHIP | Senseseq: 5'- GACGCAUUCAUCUCUGAGAAUTT-3'  Antiseq: 5'-AUUCUCAGAGAUGAAUGCGUCTT-3' |
| VHL | Senseseq: 5'-GCCUAGUCAAGCCUGAGAATT-3'  Antiseq: 5'-UUCUCAGGCUUGACUAGGCTT-3' |
| BAG1 | Senseseq: 5'-GGUUGUUGAAGAGGUCAUATT-3'  Antiseq: 5'-UAUGACCUCUUCAACAACCTT-3' |
| BAG3 | Senseseq: 5'-CAAGGCAAGAAGACUGACAAATT-3'  Antiseq: 5'-UUUGUCAGUCUUCUUGCCUUCTT-3' |
| LAMP2A | Senseseq: 5'-GUACGCUAUGAAACUACAAAUTT-3'  Antiseq: 5'-AUUUGUAGUUUCAUAGCGUACTT-3' |

**Supplementary Figures**

**Supplementary Figure S1**


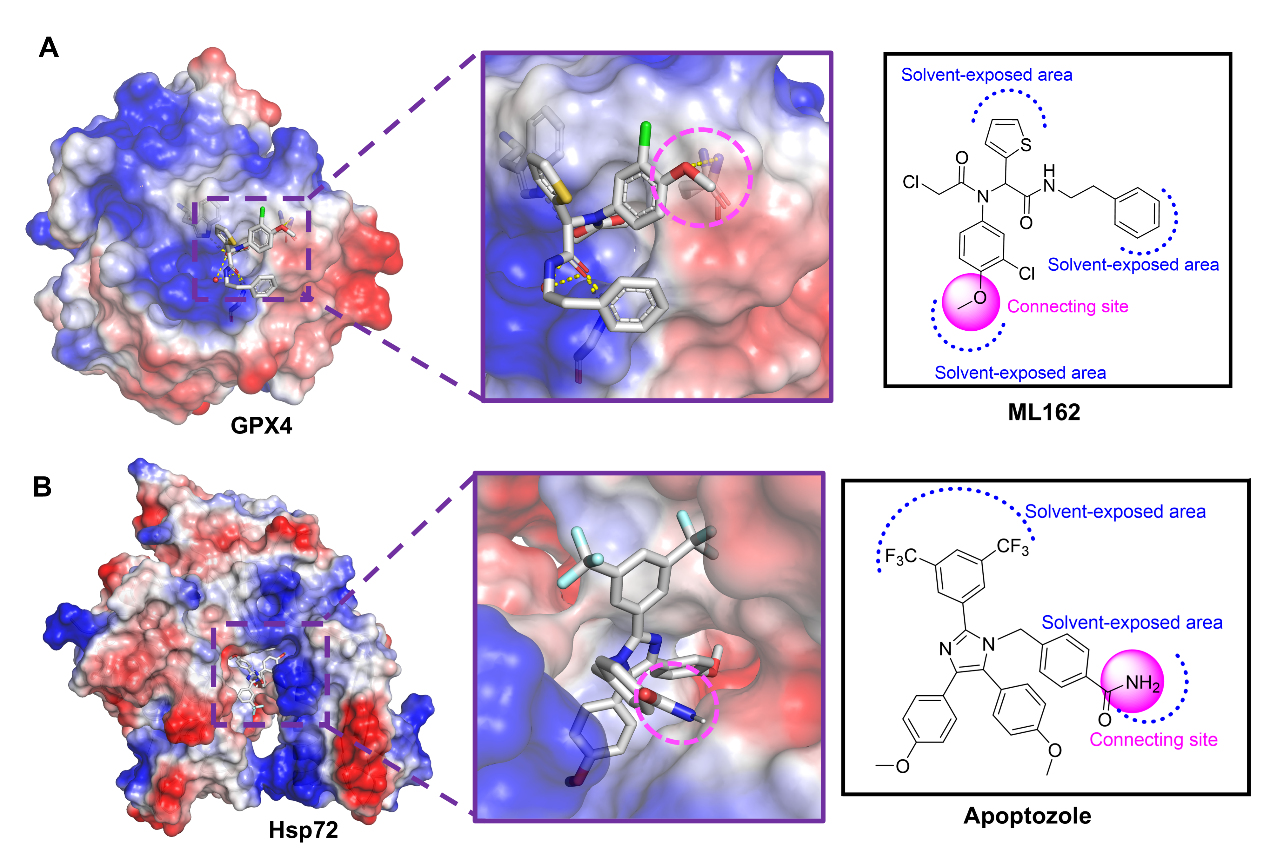


**Figure S1.** Analysis of complex structures. (A) Cocrystal structures of GPX4 with ML162 (A, PDB code: 6HKQ). (B) Docking simulation complex of Hsp72 with apoptozole

**Supplementary Figure S2**


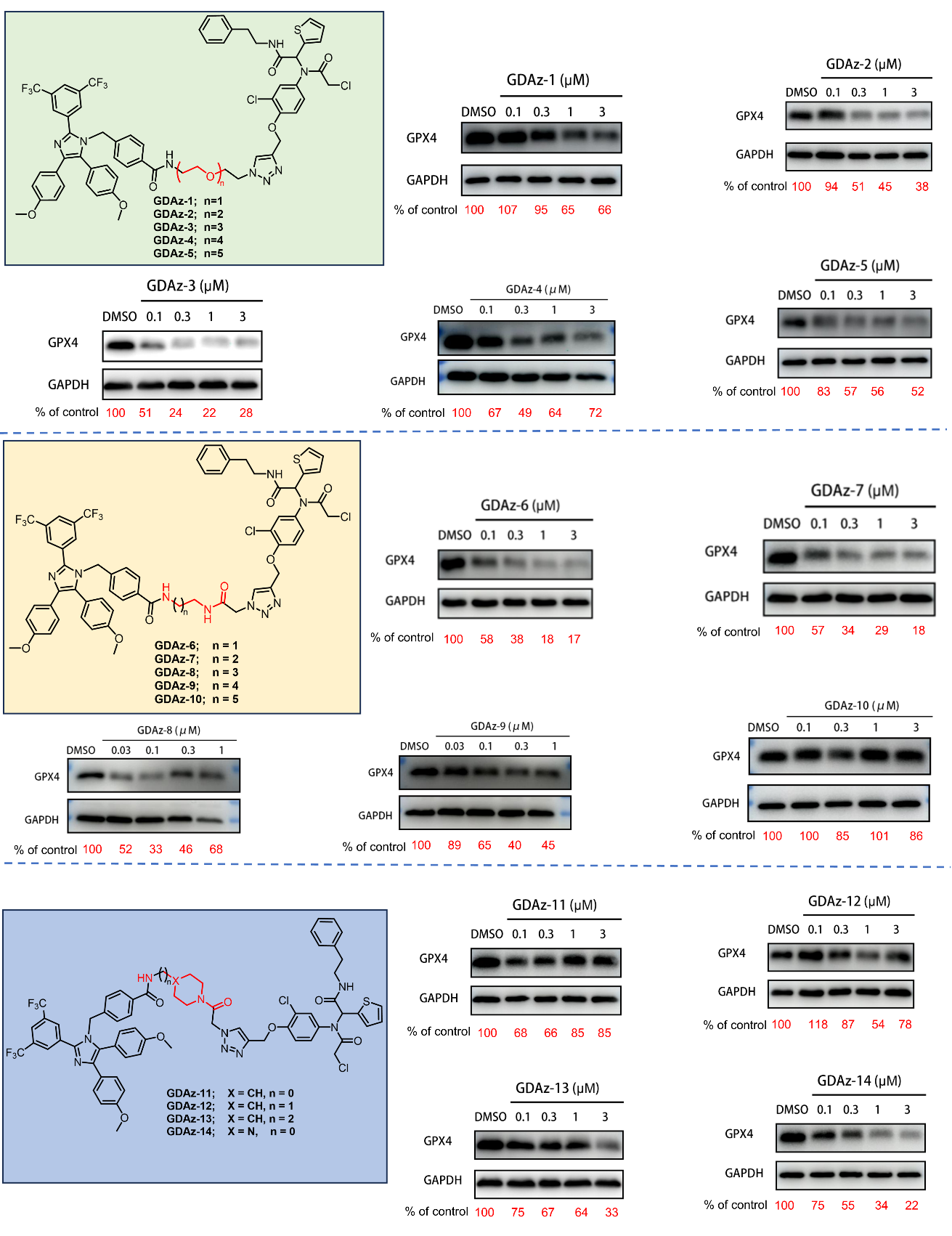


**Figure S2.** The preliminary screening of GPX4 degradation effect after treatment with different concentrations (0.1, 0.3, 1, and 3 µM) for 24 h in HT1080 cells (calculated using Image J software and the respective DMSO treatment group:100%). (GPX4: 19 KDa, GAPDH: 35 KDa).

**Supplementary Figure S3**


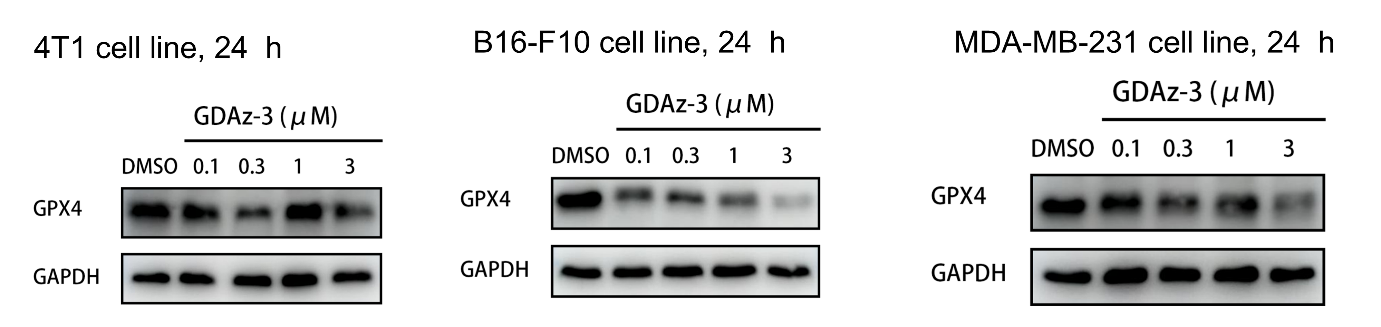


**Figure S3.** Western blot analysis of GPX4 levels in other cells after treatment with GDAz-3 for 24 h (GPX4: 19 KDa, GAPDH: 35 KDa).

**Supplementary Figure S4**


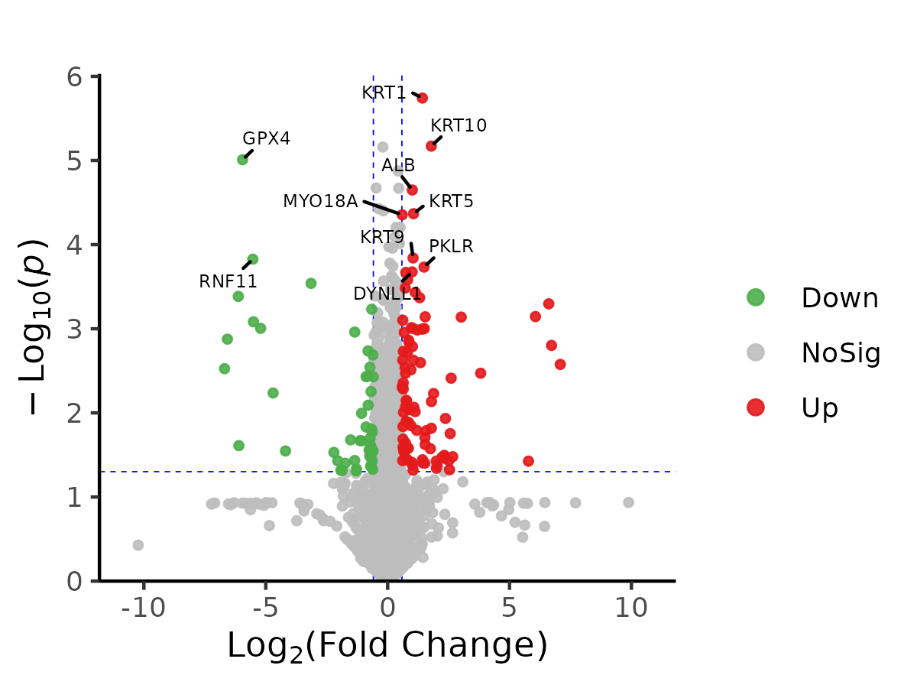


**Figure S4.** Quantitative proteomics analysis of the abundance of proteins in GDAz-3-treated HT1080 cells as compared with that in DMSO-treated HT1080 cells. (number of down regulated proteins: 206; number of up regulated: 280; and number of not sig proteins: 7650)

**Supplementary Figure S5**


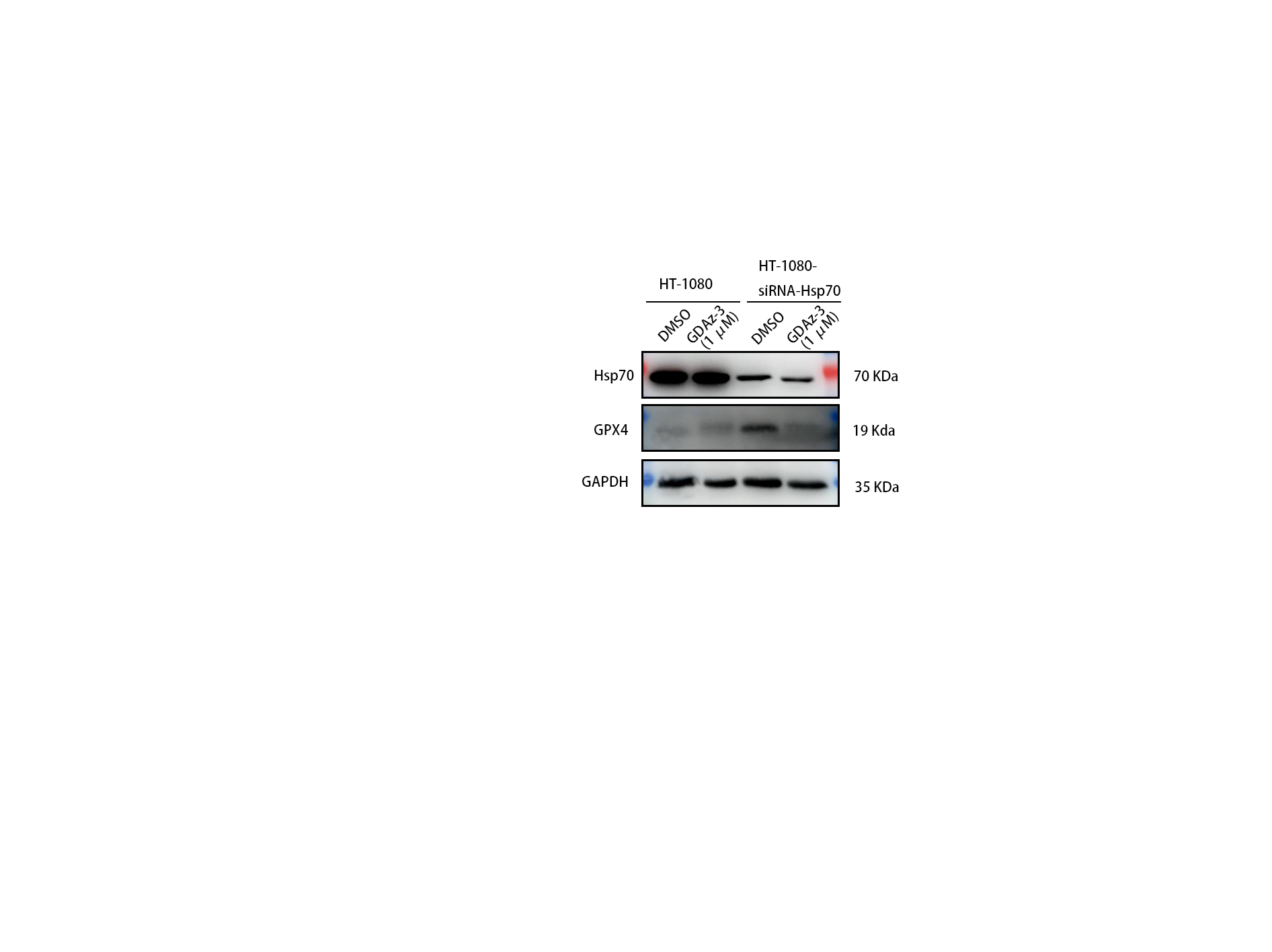


**Figure S5.** Western blot analysis of GPX4 levels in Hsp70 knockdown-HT1080 cells after treatment with GDAz-3 for 24 h.

**Supplementary Figure S6**


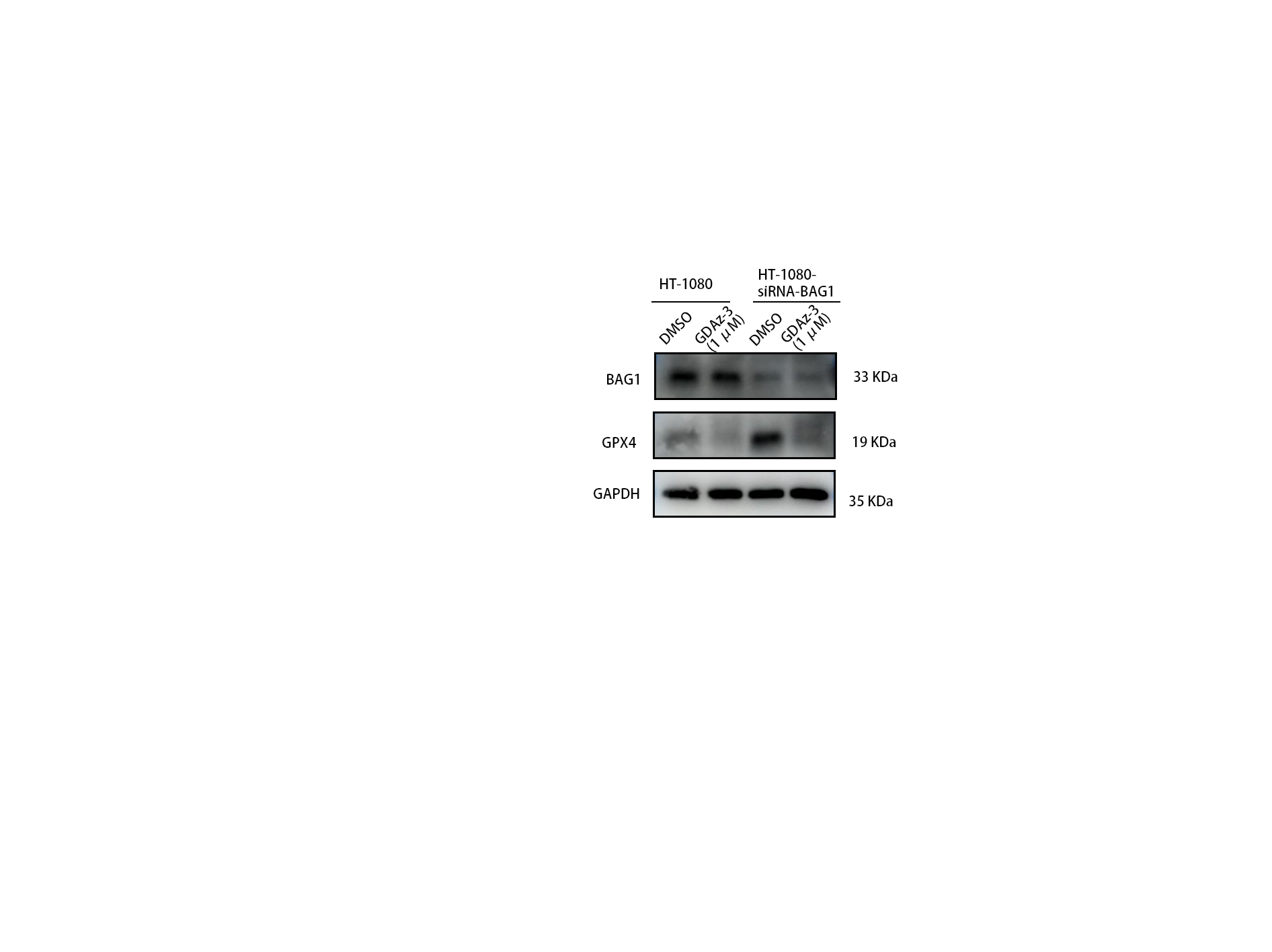


**Figure S6.** Western blot analysis of GPX4 levels in BAG1 knockdown-HT1080 cells after treatment with GDAz-3 for 24 h.

**Supplementary Figure S7**


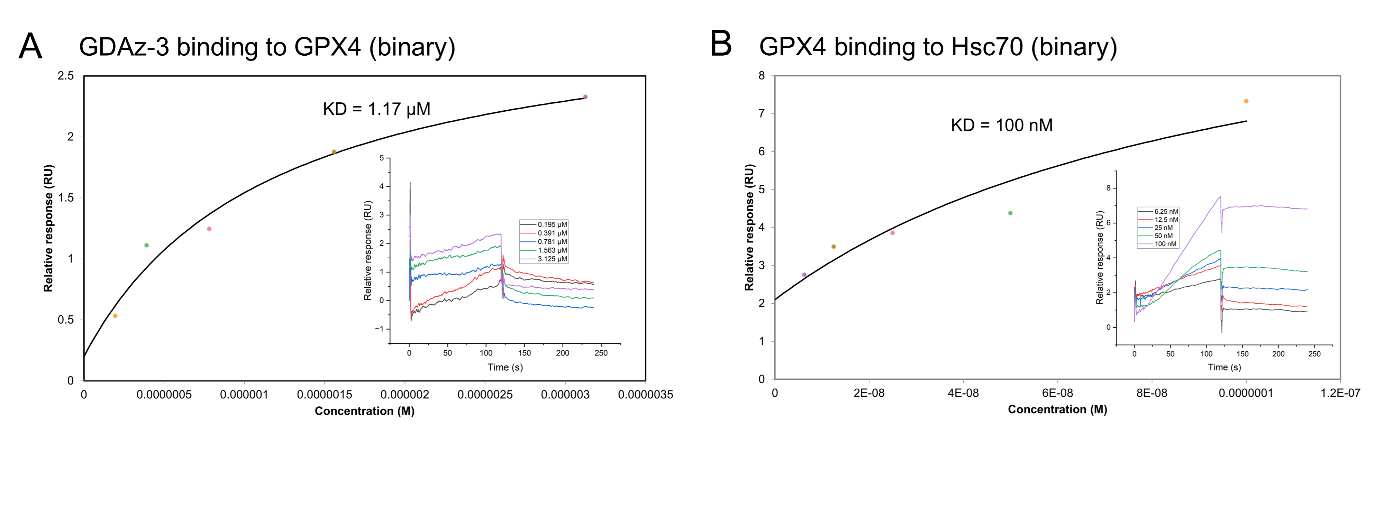


**Figure S7.** Determination of binding affinity using SPR. (A) Binding affinity of GDAz-3 with GPX4 recombinant protein; (B) Binding affinity of GPX4 recombinant protein with Hsc70 recombinant protein.

**Supplementary Figure S8**

Ten clusters were generated, and representative conformations were shown:


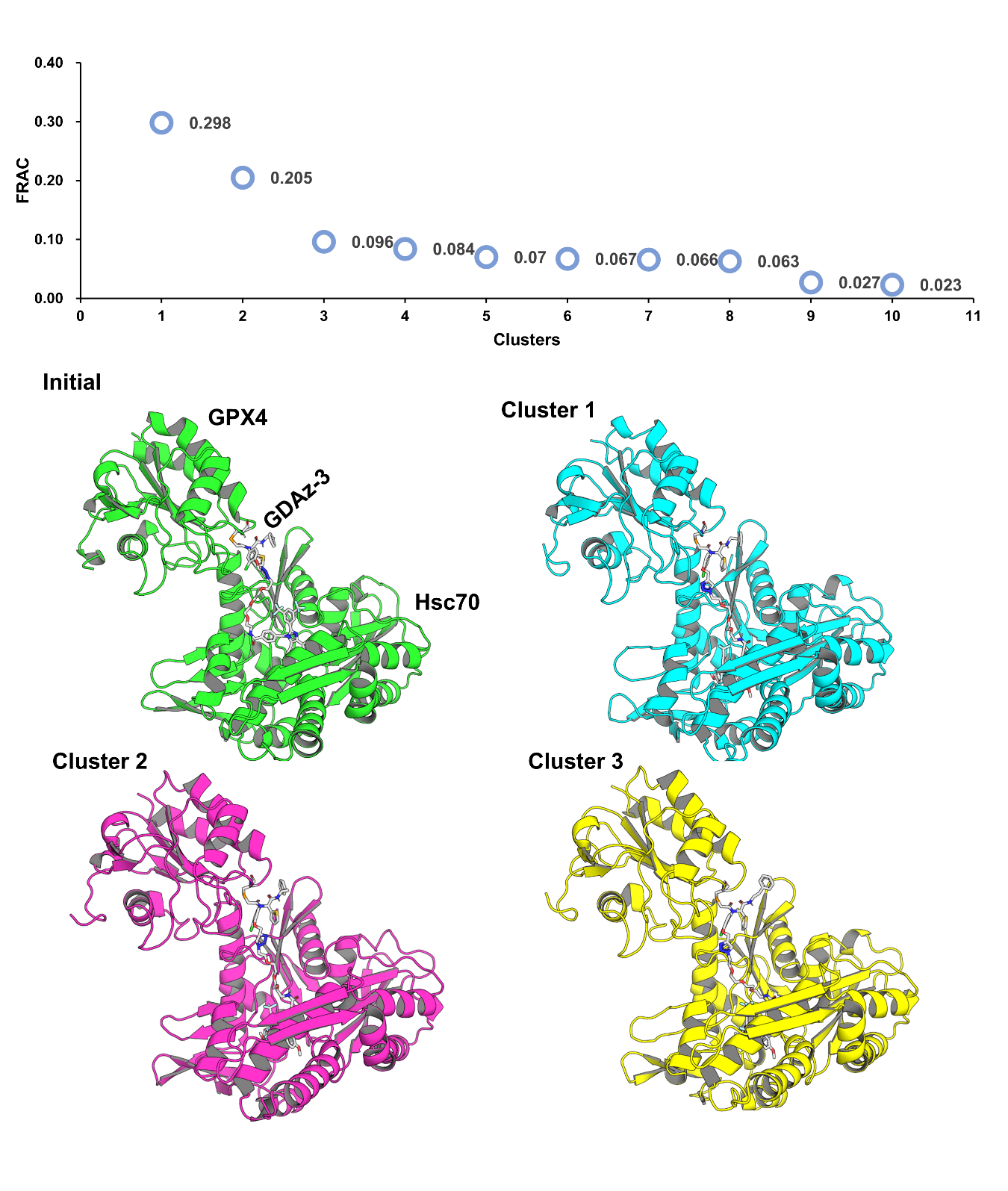


**Figure S8.** MD stimulations of Hsc70/GDAz-3/GPX4 complex. Ten clusters were generated, and representative conformations were presented.

**Supplementary Figure S9**


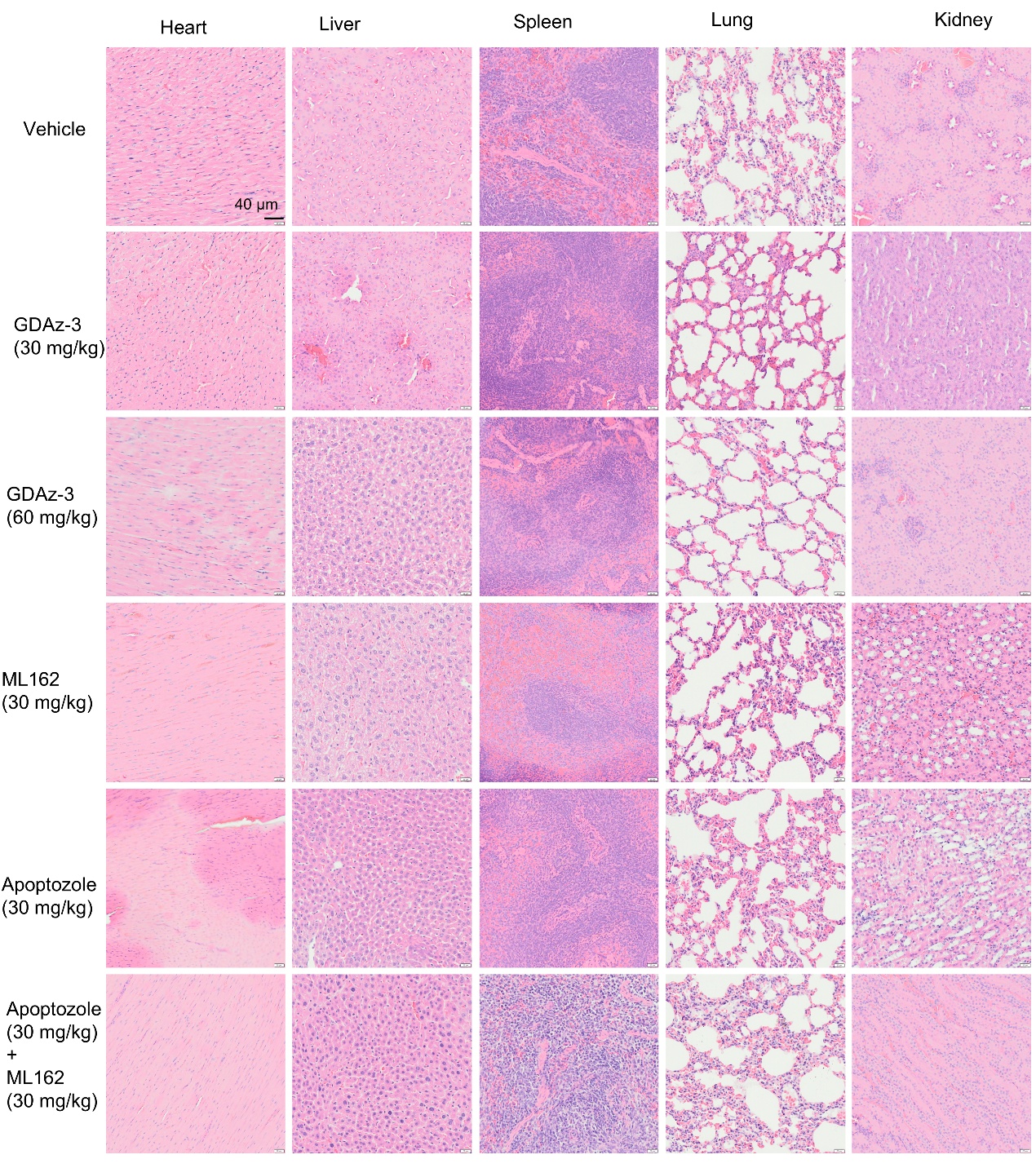


**Figure S9.** H&E analysis of the main organs.

**Supplementary Figure S10**


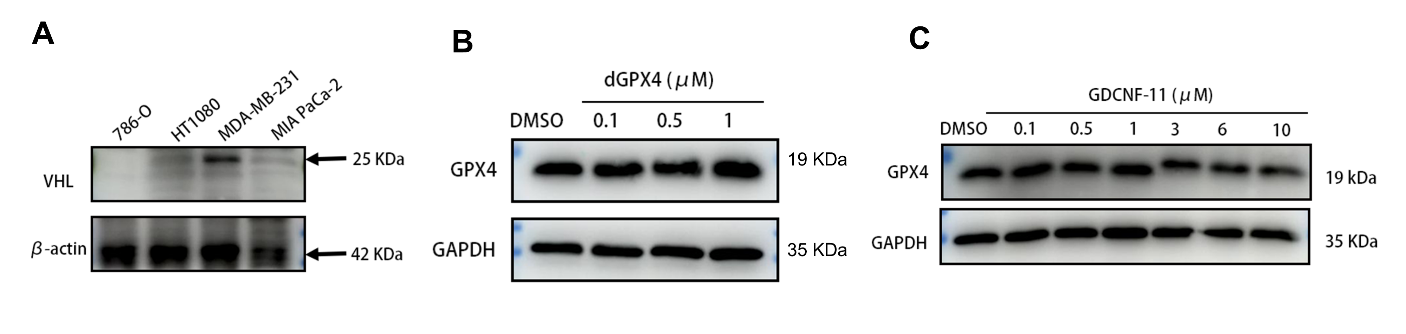


**Figure S10**. (A) Western blot analysis of VHL levels in the selected cell lines. (B, C) Western blot analysis of GPX4 levels in 786-O cells after treatment with dGPX4 and GDCNF-11 for 24 h.

.

***Materials and Methods for Chemistry***

**Chemistry Experimental Section**

Commercial reagents and solvents were used as received without further purification or drying unless otherwise noted. Anhydrous organic solvents were employed for the air- and moisture-sensitive reactions conducted in oven-dried glassware under a positive pressure of N_2_. Reaction processes were monitored by thin-layer chromatography (TLC) and visualized under UV light at wavelengths of 254 or 365 nm. Flash column chromatography was generally carried out on 200-300 mesh silica gel. The nuclear magnetic resonance (^1^H NMR and ^13^C NMR) spectra were performed on Bruker AVANCE NEO 400 spectrometer in the indicated solvent (DMSO-*d*_6_ or CDCl_3_). Chemical shift values (δ) are expressed in ppm relative to tetramethylsilane (TMS) as an internal standard, and coupling constants (*J*) are expressed in hertz (Hz). High-resolution mass spectra (HRMS) of all the target compounds were obtained on Orbitrap Exploris 120. The purity of the compounds was verified by the high-performance liquid chromatography (HPLC) analysis performed on a Hypersil GOLD aQ column (250 mm × 4.6 mm, 5 μm) using a mixture of solvent methanol (A)/water (0.1% H_3_PO_4_, B) with gradient elution (0 min: A/B = 90:10; 0~6 min: A/B = 90:10, v/v; 6~11 min: A/B = 1:99, v/v; 11~18 min: A/B = 1:99, v/v; 18~24 min: A/B = 90:10, v/v; 24~30 min: A/B = 90:10, v/v) at a flow rate of 1.0 mL/min. For compounds GDAz-8~9, the purity analysis was performed on an Amethyst C18-H column (250 mm × 4.6 mm) using a mixture of solvent CH_3_CN (A)/water (1% TEA-CH_3_COOH, B, pH 7.3-7.4) =80/20 at a flow rate of 1.0 mL/min. The purity of all the test compounds was greater than 95%.

**Scheme S1.** Synthesis of GDAz-1~14.

***Reagents and conditions***: a) Boc_2_O, THF, rt, 79%; b) propargyl bromide, K_2_CO_3_, DMF, rt, 80%; c) TFA, DCM, rt, 69%; d) chloroacetic acid, 2-thiophenecarboxaldehyde, 2-phenethylisocyanide, MeOH, rt, 27%; e) AlCl_3_, 0 ℃~ rt. 59%; f) CH_3_COONH_4_, CH_3_COOH, 100 ℃, 48%; g) EDCI/HOBT, DMF, rt; h) TFA, DCM, rt.; i) sodium ascorbate, CuSO_4_, *t*-BuOH:DMF:THF:H_2_O (V/V/V = 2/2/2/1), 0 ℃~ rt, 40-50%.

Intermediate **5** (ML162-yne) was obtained from starting material **1** according to a previously published procedure.

**1,2-bis(4-methoxyphenyl)ethane-1,2-dione (7)**: Oxalyl chloride (2.4 mL, 28.2 mmol) was slowly added to a mixture of methoxybenzene (**6**, 9.1 g, 84.8 mmol) and aluminum chloride (17 g, 200 mmol) at 0 ℃. Then, the resulting mixture was stirred at ambient temperature for 6 hours. After cooling, the mixture was poured into ice water and extracted with dichloromethane. The collected organic phase was washed with HCl aqueous solution (2N), dried with anhydrous Na_2_SO_4_, filtered, and concentrated under reduced pressure. The residue is recrystallized in ethanol. The resulting precipitate was heated, washed several times with ethanol, and dried to yield compound **7** (4.5 g, 59%) as a yellow solid.

**4-((2-(3,5-bis(trifluoromethyl)phenyl)-4,5-bis(4-methoxyphenyl)-1*H*-imidazol-1-yl)methyl)benzoic acid (8)**: A solution of 4-(aminomethyl)benzoic acid (2 g, 13.2 mmol), 3,5-bis(trifluoromethyl)benzaldehyde (3.27 mL, 19.8 mmol), 1,2-bis(4-methoxyphenyl)ethane-1,2-dione (**7**, 5.36 g, 19.8 mmol) and ammonium acetate (6.17 g, 79.4 mmol) in acetic acid was stirred for 12 h at 100 ℃. After cooling to room temperature, the reaction mixture was diluted with ethyl acetate, and washed with water, saturated NaHCO_3_ and brine. The organic layer was dried with anhydrous Na_2_SO_4_, filtered and concentrated under reduced pressure. The residue was purified by flash column chromatography (DCM:MeOH = 15:1) and dried to give compound **8** (3.9 g, 48%) as a white solid.

**Intermediates 9** and **10**: To a stirred solution of **8** (1 eq) in DMF was added 1-Ethyl-3-(3-dimethylaminopropyl)carbodiimide hydrochloride (EDCI) (1.2 eq) and 1-hydroxybenzotriazole (HOBt) (1.3 eq). After stirring for 1 h at room temperature, the designated amino substituted derivative (1.5 eq) was added to the mixture. After stirring for 2 h, the mixture was diluted with EtOAc, washed with brine, dried over anhydrous Na_2_SO_4_, and concentrated under reduced pressure. The residue was purified by flash column chromatography (DCM:MeOH = 15:1) to give the corresponding product.

**Intermediate** **11**: Compound **10** was dissolved in the solution of DCM/TFA (2:1), and the mixture was stirred at room temperature for 2 h. The reaction solution was concentrated under reduced pressure to obtain the BOC-deprotect intermediate, which was further added to a mixture of HATU (1.5 eq), DIPEA (3 eq), and 2-azidoacetic acid (1 eq) in dry DMF with stirring for 12 h at room temperature. After the reaction was completed, water was added and extracted with ethyl acetate. The combined organic layer was washed with brine, dried over anhydrous Na_2_SO_4_, filtered, and evaporated under reduced pressure. The crude residue was purified by silica gel column chromatography to afford the azido-substituted derivative **11**.

**Title compounds GDAz-1~12**: To an aqueous solution of sodium ascorbate (2.5 eq) and copper sulfate pentahydrate (0.5 eq) was added a mixture solution (*t*-BuOH:DMF:THF:H_2_O = 2/2/2/1) of ML162-yne (**5**, 1.1 eq) and azido-substituted derivative **9** or **11** (1 eq) at 0 °C, the resulting mixture was stirred under a nitrogen atmosphere for overnight. The reaction solution was diluted with water, extracted with DCM, washed with brine, dried over Na_2_SO_4_, filtered, and concentrated. The residue was purified by column chromatography (DCM:MeOH = 15 : 1) to give title compounds **GDAz-1~12**.

*4-((2-(3,5-bis(trifluoromethyl)phenyl)-4,5-bis(4-methoxyphenyl)-1H-imidazol-1-yl)methyl)-N-(2-(2-(4-((2-chloro-4-(2-chloro-N-(2-oxo-2-(phenethylamino)-1-(thiophen-2-yl)ethyl)acetamido)phenoxy)methyl)-1H-1,2,3-triazol-1-yl)ethoxy)ethyl)benzamide (****GDAz-1****)*

^1^H NMR (400 MHz, CDCl_3_) δ 7.98 (d, *J* = 1.7 Hz, 2H), 7.74 (s, 1H), 7.71 – 7.64 (m, 3H), 7.46 – 7.40 (m, 2H), 7.18 – 7.05 (m, 6H), 7.04 – 7.00 (m, 2H), 6.89 (d, *J* = 8.0 Hz, 2H), 6.85 – 6.77 (m, 3H), 6.77 – 6.68 (m, 5H), 6.18 (t, *J* = 5.9 Hz, 1H), 6.01 (s, 1H), 5.06 (s, 3H), 4.46 (t, *J* = 4.9 Hz, 2H), 3.80 (t, *J* = 5.0 Hz, 2H), 3.72 (d, *J* = 5.5 Hz, 5H), 3.68 (s, 3H), 3.53 (t, *J* = 4.2 Hz, 3H), 3.46 – 3.41 (m, 1H), 2.81 (d, *J* = 31.6 Hz, 3H), 2.72 – 2.67 (m, 1H). ^13^C NMR (101 MHz, CDCl_3_) δ 166.99, 165.71, 165.59, 161.56, 159.16, 157.61, 153.18, 143.11, 142.15, 139.43, 137.81, 137.60, 133.75, 132.76, 131.22, 131.05, 130.77, 130.71, 130.57, 129.45, 129.09, 128.39, 127.79, 127.59, 127.55, 127.22, 126.96, 126.86, 125.54, 125.46, 125.43, 124.81, 123.33, 122.95, 121.18, 120.91, 120.62, 113.65, 112.71, 112.51, 68.74, 67.92, 62.12, 54.27, 54.17, 49.23, 47.13, 41.38, 40.04, 38.61, 35.48, 34.41. HRMS (ESI) calcd for [C_62_H_54_Cl_2_F_6_N_8_O_7_S + H]^+^ 1239.3196, found 1239.3181; HPLC purity: 96.669%, retention time = 15.051 min.

*4-((2-(3,5-bis(trifluoromethyl)phenyl)-4,5-bis(4-methoxyphenyl)-1H-imidazol-1-yl)methyl)-N-(2-(2-(2-(4-((2-chloro-4-(2-chloro-N-(2-oxo-2-(phenethylamino)-1-(thiophen-2-yl)ethyl)acetamido)phenoxy)methyl)-1H-1,2,3-triazol-1-yl)ethoxy)ethoxy)ethyl)benzamide (****GDAz-2****)*

^1^H NMR (400 MHz, CDCl_3_) δ 7.99 (d, *J* = 1.7 Hz, 2H), 7.74 (d, *J* = 5.3 Hz, 2H), 7.65 (d, *J* = 8.0 Hz, 2H), 7.44 (d, *J* = 8.9 Hz, 2H), 7.17 (d, *J* = 7.5 Hz, 3H), 7.11 (dd, *J* = 7.8, 4.0 Hz, 3H), 7.05 (d, *J* = 7.0 Hz, 2H), 6.89 (d, *J* = 8.0 Hz, 3H), 6.84 – 6.74 (m, 4H), 6.72 (d, *J* = 8.9 Hz, 2H), 6.65 (d, *J* = 5.4 Hz, 1H), 6.01 (d, *J* = 12.3 Hz, 2H), 5.14 (s, 1H), 5.06 (s, 2H), 4.44 (t, *J* = 5.0 Hz, 2H), 3.80 (t, *J* = 5.1 Hz, 2H), 3.73 (d, *J* = 6.4 Hz, 5H), 3.70 (s, 3H), 3.52 (d, *J* = 6.0 Hz, 8H), 3.46 (dd, *J* = 14.6, 6.9 Hz, 2H), 2.77 – 2.65 (m, 2H). ^13^C NMR (101 MHz, CDCl_3_) δ 166.92, 165.65, 165.61, 159.15, 157.62, 153.27, 143.09, 142.04, 139.35, 137.79, 137.57, 133.70, 133.00, 131.20, 131.07, 130.81, 130.73, 130.53, 129.41, 129.10, 128.35, 127.80, 127.57, 127.29, 126.95, 126.77, 125.54, 125.49, 124.81, 123.32, 123.14, 122.14, 121.21, 120.88, 120.61, 113.65, 112.72, 112.56, 69.47, 69.15, 68.78, 68.28, 62.22, 59.74, 54.28, 54.18, 49.31, 47.12, 41.33, 40.04, 38.75, 34.43. HRMS (ESI) calcd for [C_64_H_58_Cl_2_F_6_N_8_O_8_S + H]^+^ 1283.3458, found 1283.3431; HPLC purity: 96.802%, retention time = 15.067 min.

*4-((2-(3,5-bis(trifluoromethyl)phenyl)-4,5-bis(4-methoxyphenyl)-1H-imidazol-1-yl)methyl)-N-(2-(2-(2-(2-(4-((2-chloro-4-(2-chloro-N-(2-oxo-2-(phenethylamino)-1-(thiophen-2-yl)ethyl) acetamido)phenoxy)methyl)-1H-1,2,3-triazol-1-yl)ethoxy)ethoxy)ethoxy)ethyl)benzamide (****GDAz-3****)*

^1^H NMR (400 MHz, CDCl_3_) δ 8.01 (s, 2H), 7.76 (d, *J* = 8.0 Hz, 2H), 7.67 (d, *J* = 7.9 Hz, 2H), 7.45 (d, *J* = 8.8 Hz, 2H), 7.19 – 7.15 (m, 3H), 7.11 (t, *J* = 7.5 Hz, 4H), 7.07 – 7.03 (m, 2H), 6.88 (d, *J* = 8.0 Hz, 2H), 6.86 – 6.75 (m, 6H), 6.73 (d, *J* = 8.9 Hz, 2H), 5.99 (s, 1H), 5.13 (s, 2H), 5.07 (s, 2H), 4.41 (t, *J* = 5.0 Hz, 2H), 3.80 – 3.67 (m, 11H), 3.60 – 3.41 (m, 15H), 2.88 (s, 1H), 2.81 (s, 1H). ^13^C NMR (101 MHz, CDCl_3_) δ 166.91, 165.62, 165.56, 161.55, 159.26, 157.80, 153.29, 142.88, 141.94, 137.56, 133.69, 133.14, 131.22, 131.15, 130.81, 130.51, 129.37, 129.10, 128.33, 127.80, 127.73, 127.58, 127.31, 127.07, 126.88, 125.54, 125.50, 124.73, 123.27, 123.24, 122.13, 121.51, 120.56, 113.70, 112.78, 112.55, 69.47, 69.34, 69.13, 68.76, 68.28, 62.20, 54.30, 54.20, 49.29, 47.20, 41.31, 40.04, 38.78, 34.44. HRMS (ESI) calcd for [C_66_H_62_Cl_2_F_6_N_8_O_9_S + H]^+^ 1327.3720, found 1327.3690; HPLC purity: 95.160%, retention time = 15.052 min.

*4-((2-(3,5-bis(trifluoromethyl)phenyl)-4,5-bis(4-methoxyphenyl)-1H-imidazol-1-yl)methyl)-N-(14-(4-((2-chloro-4-(2-chloro-N-(2-oxo-2-(phenethylamino)-1-(thiophen-2-yl)ethyl)acetamido)phenoxy)methyl)-1H-1,2,3-triazol-1-yl)-3,6,9,12-tetraoxatetradecyl)benzamide (****GDAz-4****)*

^1^H NMR (400 MHz, CDCl_3_) δ 8.00 (d, *J* = 1.6 Hz, 2H), 7.77 (d, *J* = 11.4 Hz, 2H), 7.70 (d, *J* = 8.1 Hz, 2H), 7.43 (d, *J* = 8.8 Hz, 2H), 7.16 (d, *J* = 7.5 Hz, 3H), 7.13 – 7.07 (m, 4H), 7.04 (d, *J* = 6.9 Hz, 3H), 6.88 (d, *J* = 8.0 Hz, 3H), 6.83 – 6.74 (m, 4H), 6.72 (d, *J* = 8.9 Hz, 2H), 6.08 (t, *J* = 5.8 Hz, 1H), 6.00 (s, 1H), 5.13 (s, 1H), 5.06 (s, 2H), 4.41 (t, *J* = 5.0 Hz, 2H), 3.73 (d, *J* = 3.7 Hz, 7H), 3.69 (s, 3H), 3.54 (t, *J* = 8.6 Hz, 10H), 3.48 (d, *J* = 18.0 Hz, 8H), 2.83 (d, *J* = 30.4 Hz, 1H), 2.75 – 2.69 (m, 1H). ^13^C NMR (101 MHz, CDCl_3_) δ 166.95, 165.62, 165.58, 159.15, 157.61, 153.32, 143.07, 141.87, 139.21, 137.79, 137.59, 133.73, 133.06, 131.19, 131.09, 130.75, 130.73, 130.53, 129.44, 129.10, 128.35, 127.80, 127.56, 127.50, 127.26, 126.93, 125.54, 125.48, 124.65, 123.39, 123.32, 121.18, 120.89, 120.61, 113.65, 112.71, 112.53, 69.45, 69.43, 69.41, 69.39, 69.29, 69.11, 68.90, 68.29, 62.15, 54.28, 54.18, 49.26, 47.14, 41.35, 40.04, 38.81, 34.43. HRMS (ESI) calcd for [C_68_H_66_Cl_2_F_6_N_8_O_10_S + H]^+^ 1371.3982, found 1371.3958; HPLC purity: 95.816%, retention time = 15.056 min.

*4-((2-(3,5-bis(trifluoromethyl)phenyl)-4,5-bis(4-methoxyphenyl)-1H-imidazol-1-yl)methyl)-N-(17-(4-((2-chloro-4-(2-chloro-N-(2-oxo-2-(phenethylamino)-1-(thiophen-2-yl)ethyl)acetamido)phenoxy)methyl)-1H-1,2,3-triazol-1-yl)-3,6,9,12,15-pentaoxaheptadecyl)benzamide (****GDAz-5****)*

^1^H NMR (400 MHz, CDCl_3_) δ 8.00 (d, *J* = 1.7 Hz, 2H), 7.81 (s, 1H), 7.77 – 7.69 (m, 3H), 7.45 – 7.40 (m, 2H), 7.26 (s, 1H), 7.19 – 7.06 (m, 7H), 7.05 – 7.01 (m, 2H), 6.88 (d, *J* = 8.1 Hz, 2H), 6.86 – 6.73 (m, 5H), 6.73 – 6.69 (m, 2H), 6.14 (t, *J* = 5.9 Hz, 1H), 6.01 (s, 1H), 5.14 (s, 1H), 5.06 (s, 2H), 4.43 (t, *J* = 5.0 Hz, 2H), 3.73 (s, 7H), 3.69 (s, 3H), 3.60 – 3.49 (m, 13H), 3.46 (d, *J* = 8.1 Hz, 9H), 2.87 – 2.68 (m, 2H). ^13^C NMR (101 MHz, CDCl_3_) δ 166.98, 165.61, 165.57, 159.13, 157.58, 153.34, 143.09, 141.83, 139.18, 137.85, 137.61, 133.76, 133.04, 131.82, 131.18, 131.07, 130.74, 130.68, 130.54, 129.46, 129.10, 128.37, 127.80, 127.55, 127.50, 127.46, 127.24, 127.00, 126.92, 125.55, 125.51, 125.46, 124.62, 123.48, 123.33, 122.08, 121.13, 120.94, 120.62, 113.63, 112.70, 112.53, 69.43, 69.40, 69.37, 69.31, 69.07, 68.90, 68.27, 62.13, 59.67, 54.27, 54.18, 49.28, 47.15, 41.38, 40.05, 38.83, 34.43. HRMS (ESI) calcd for [C_70_H_70_Cl_2_F_6_N_8_O_11_S + H]^+^ 1415.4244, found 1415.4225; HPLC purity: 95.264%, retention time = 15.038 min.

*4-((2-(3,5-bis(trifluoromethyl)phenyl)-4,5-bis(4-methoxyphenyl)-1H-imidazol-1-yl)methyl)-N-(2-(2-(4-((2-chloro-4-(2-chloro-N-(2-oxo-2-(phenethylamino)-1-(thiophen-2-yl)ethyl)acetamido)phenoxy)methyl)-1H-1,2,3-triazol-1-yl)acetamido)ethyl)benzamide (****GDAz-6****)*

^1^H NMR (400 MHz, CDCl_3_) δ 7.96 (d, *J* = 1.7 Hz, 2H), 7.73 (d, *J* = 14.8 Hz, 3H), 7.63 (d, *J* = 8.1 Hz, 2H), 7.51 – 7.45 (m, 1H), 7.43 – 7.37 (m, 2H), 7.12 – 7.07 (m, 5H), 7.06 – 7.01 (m, 1H), 6.99 – 6.94 (m, 2H), 6.86 (d, *J* = 8.0 Hz, 2H), 6.80 – 6.75 (m, 2H), 6.72 – 6.68 (m, 3H), 6.67 (d, *J* = 2.1 Hz, 1H), 6.40 (t, *J* = 5.9 Hz, 1H), 6.02 (s, 1H), 5.03 (d, *J* = 5.7 Hz, 4H), 4.95 (s, 2H), 3.71 (s, 2H), 3.68 (s, 3H), 3.64 (s, 3H), 3.42 (d, *J* = 6.5 Hz, 2H), 3.37 – 3.31 (m, 2H), 2.65 (dq, *J* = 14.1, 6.9 Hz, 4H). ^13^C NMR (101 MHz, CDCl_3_) δ 167.17, 166.57, 165.76, 165.37, 159.13, 157.59, 153.16, 143.11, 142.21, 139.64, 137.84, 137.56, 133.70, 132.33, 131.76, 131.19, 131.00, 130.70, 130.66, 130.57, 129.52, 129.20, 128.43, 127.74, 127.52, 127.24, 126.98, 126.91, 125.61, 125.47, 125.43, 124.83, 124.08, 123.32, 121.16, 120.86, 120.61, 113.65, 112.73, 112.54, 61.87, 59.75, 54.24, 54.15, 51.52, 47.11, 41.64, 40.06, 39.23, 38.96, 34.31. HRMS (ESI) calcd for [C_62_H_53_Cl_2_F_6_N_9_O_7_S + H]^+^ 1252.3148, found 1252.3135; HPLC purity: 95.779%, retention time = 14.957 min.

*4-((2-(3,5-bis(trifluoromethyl)phenyl)-4,5-bis(4-methoxyphenyl)-1H-imidazol-1-yl)methyl)-N-(3-(2-(4-((2-chloro-4-(2-chloro-N-(2-oxo-2-(phenethylamino)-1-(thiophen-2-yl)ethyl)acetamido)phenoxy)methyl)-1H-1,2,3-triazol-1-yl)acetamido)propyl)benzamide (****GDAz-7****)*

^1^H NMR (400 MHz, CDCl_3_) δ 7.96 (d, *J* = 1.7 Hz, 2H), 7.81 (d, *J* = 6.9 Hz, 1H), 7.74 – 7.63 (m, 4H), 7.47 – 7.37 (m, 3H), 7.11 – 7.05 (m, 5H), 7.05 – 7.01 (m, 1H), 6.97 – 6.93 (m, 2H), 6.85 (d, *J* = 8.0 Hz, 2H), 6.79 – 6.74 (m, 2H), 6.71 – 6.65 (m, 4H), 6.47 (d, *J* = 5.9 Hz, 1H), 6.03 (s, 1H), 5.12 – 4.94 (m, 6H), 3.73 – 3.60 (m, 8H), 3.24 (dq, *J* = 33.5, 6.0 Hz, 4H), 2.74 (d, *J* = 12.1 Hz, 2H), 2.64 (dd, *J* = 15.4, 8.2 Hz, 2H), 1.91 (s, 1H), 1.60 (s, 1H). ^13^C NMR (101 MHz, CDCl_3_) δ 168.24, 167.04, 166.76, 165.88, 160.17, 158.62, 154.24, 144.15, 143.16, 140.47, 138.86, 138.60, 134.77, 133.68, 132.83, 132.21, 132.04, 131.70, 130.59, 130.23, 128.75, 128.57, 128.53, 128.23, 128.02, 127.86, 126.64, 126.52, 126.45, 125.83, 125.13, 124.35, 121.87, 121.64, 114.67, 113.74, 62.94, 60.43, 55.25, 55.16, 48.15, 42.68, 41.11, 36.54, 35.34, 29.03. HRMS (ESI) calcd for [C_63_H_55_Cl_2_F_6_N_9_O_7_S + H]^+^ 1266.3305, found 1266.3284; HPLC purity: 98.087%, retention time = 14.957 min.

4-((2-(3,5-bis(trifluoromethyl)phenyl)-4,5-bis(4-methoxyphenyl)-1*H*-imidazol-1-yl)methyl)-*N*-(4-(2-(4-((2-chloro-4-(2-chloro-*N*-(2-oxo-2-(phenethylamino)-1-(thiophen-2-yl)ethyl)acetamido)phenoxy)methyl)-1*H*-1,2,3-triazol-1-yl)acetamido)butyl)benzamide *(****GDAz-8****)*

^1^H NMR (400 MHz, CDCl_3_) δ 8.01 – 7.95 (m, 2H), 7.91 (s, 1H), 7.81 (s, 1H), 7.74 (s, 1H), 7.65 (d, *J* = 8.2 Hz, 2H), 7.45 – 7.39 (m, 2H), 7.17 – 7.13 (m, 3H), 7.12 – 7.08 (m, 3H), 7.04 – 7.00 (m, 2H), 6.88 (d, *J* = 8.0 Hz, 3H), 6.81 (d, *J* = 2.1 Hz, 1H), 6.80 (d, *J* = 1.9 Hz, 1H), 6.78 – 6.74 (m, 3H), 6.72 (d, *J* = 2.1 Hz, 1H), 6.71 (d, *J* = 2.1 Hz, 1H), 6.00 (s, 1H), 5.13 (s, 2H), 5.05 (s, 2H), 4.99 (s, 2H), 3.73 (s, 5H), 3.69 (s, 3H), 3.50 – 3.39 (m, 2H), 3.32 (q, *J* = 6.6, 5.9 Hz, 2H), 3.20 (p, *J* = 6.4 Hz, 2H), 2.76 – 2.66 (m, 2H), 1.55 – 1.50 (m, 2H), 1.22 – 1.19 (m, 2H). ^13^C NMR (101 MHz, CDCl_3_) δ 167.02, 166.02, 165.68, 164.13, 161.59, 159.18, 157.65, 153.23, 143.12, 142.36, 139.42, 137.86, 137.56, 133.71, 132.93, 131.78, 131.21, 131.08, 130.87, 130.75, 130.57, 129.48, 129.16, 128.36, 127.78, 127.53, 127.29, 126.97, 126.78, 125.59, 125.49, 125.44, 124.82, 122.27, 121.19, 120.90, 120.62, 113.67, 112.74, 112.61, 62.05, 59.80, 54.28, 54.19, 51.79, 47.13, 41.43, 40.07, 38.53, 38.44, 35.50, 34.41, 30.56. HRMS (ESI) calcd for [C_64_H_57_Cl_2_F_6_N_9_O_7_S + H]^+^ 1280.3461, found 1280.3439; HPLC purity: 95.58%, retention time = 6.366 min.

4-((2-(3,5-bis(trifluoromethyl)phenyl)-4,5-bis(4-methoxyphenyl)-1*H*-imidazol-1-yl)methyl)-*N*-(5-(2-(4-((2-chloro-4-(2-chloro-*N*-(2-oxo-2-(phenethylamino)-1-(thiophen-2-yl)ethyl)acetamido)phenoxy)methyl)-1*H*-1,2,3-triazol-1-yl)acetamido)pentyl)benzamide *(****GDAz-9****)*

^1^H NMR (400 MHz, CDCl_3_) δ 7.98 (s, 2H), 7.92 (s, 1H), 7.82 (s, 1H), 7.74 (s, 1H), 7.65 (d, *J* = 7.9 Hz, 2H), 7.43 (d, *J* = 8.4 Hz, 2H), 7.19 – 7.14 (m, 3H), 7.13 – 7.07 (m, 3H), 7.05 – 7.00 (m, 2H), 6.91 (t, *J* = 14.7 Hz, 4H), 6.84 – 6.79 (m, 2H), 6.76 (p, *J* = 3.1, 2.5 Hz, 2H), 6.71 (d, *J* = 8.4 Hz, 2H), 6.01 (s, 1H), 5.13 (s, 2H), 5.06 (s, 2H), 4.98 (s, 2H), 3.73 (d, *J* = 1.5 Hz, 5H), 3.69 (s, 3H), 3.46 (ddq, *J* = 19.8, 13.6, 6.6 Hz, 2H), 3.32 (q, *J* = 6.6 Hz, 2H), 3.17 (q, *J* = 6.3 Hz, 2H), 2.77 – 2.64 (m, 2H), 1.56 – 1.42 (m, 4H), 1.33 – 1.25 (m, 2H). ^13^C NMR (101 MHz, CDCl_3_) δ 166.98, 165.95, 165.64, 163.97, 161.56, 159.19, 157.65, 153.24, 143.10, 139.36, 137.83, 137.57, 133.73, 133.09, 131.77, 131.21, 131.08, 130.87, 130.75, 130.59, 129.47, 129.14, 128.37, 127.78, 127.57, 127.28, 126.97, 126.72, 125.58, 125.49, 124.85, 123.33, 122.26, 121.19, 120.89, 120.61, 117.90, 113.67, 112.74, 112.59, 62.05, 59.76, 54.28, 54.19, 51.86, 47.14, 41.42, 40.06, 38.60, 38.55, 35.49, 34.42, 30.43, 28.09. HRMS (ESI) calcd for [C_65_H_59_Cl_2_F_6_N_9_O_7_S + H]^+^ 1294.3618, found 1294.3590; HPLC purity: 96.24%, retention time = 6.757 min.

*4-((2-(3,5-bis(trifluoromethyl)phenyl)-4,5-bis(4-methoxyphenyl)-1H-imidazol-1-yl)methyl)-N-(6-(2-(4-((2-chloro-4-(2-chloro-N-(2-oxo-2-(phenethylamino)-1-(thiophen-2-yl)ethyl)acetamido)phenoxy)methyl)-1H-1,2,3-triazol-1-yl)acetamido)hexyl)benzamide (****GDAz-10****)*

^1^H NMR (400 MHz, CDCl_3_) δ 7.97 (s, 2H), 7.81 (s, 1H), 7.74 (s, 1H), 7.64 (d, *J* = 8.4 Hz, 2H), 7.42 (d, *J* = 8.9 Hz, 2H), 7.17 – 7.12 (m, 3H), 7.10 (dd, *J* = 9.0, 2.5 Hz, 3H), 7.04 – 7.00 (m, 2H), 6.97 (t, *J* = 5.8 Hz, 1H), 6.88 (d, *J* = 8.1 Hz, 3H), 6.80 (d, *J* = 8.8 Hz, 2H), 6.75 (d, *J* = 5.2 Hz, 2H), 6.71 (d, *J* = 8.9 Hz, 2H), 6.55 (t, *J* = 5.9 Hz, 1H), 6.18 (t, *J* = 5.9 Hz, 1H), 6.01 (s, 1H), 5.12 (s, 1H), 5.05 (s, 2H), 4.98 (s, 2H), 3.75 – 3.67 (m, 8H), 3.51 – 3.36 (m, 2H), 3.30 (q, *J* = 6.7 Hz, 2H), 3.15 (q, *J* = 6.5 Hz, 2H), 2.74 – 2.65 (m, 2H), 1.49 – 1.38 (m, 3H), 1.24 (s, 4H), 1.18 (d, *J* = 7.1 Hz, 1H). ^13^C NMR (101 MHz, CDCl_3_) δ 167.02, 165.88, 165.67, 163.99, 159.15, 157.62, 153.21, 143.11, 142.31, 139.32, 137.79, 137.56, 133.68, 133.11, 131.72, 131.19, 131.05, 130.80, 130.72, 130.57, 129.47, 129.16, 128.37, 127.77, 127.59, 127.56, 127.29, 126.97, 126.69, 125.59, 125.48, 125.38, 124.83, 123.96, 123.31, 121.20, 120.85, 120.60, 113.65, 112.72, 112.54, 61.98, 59.76, 54.27, 54.18, 51.80, 47.11, 41.47, 40.06, 38.49, 38.33, 34.38, 28.30, 27.82, 24.80, 24.73. HRMS (ESI) calcd for [C_66_H_61_Cl_2_F_6_N_9_O_7_S + H]^+^ 1308.3774, found 1308.3755; HPLC purity: 95.242%, retention time = 15.011 min.

*4-((2-(3,5-bis(trifluoromethyl)phenyl)-4,5-bis(4-methoxyphenyl)-1H-imidazol-1-yl)methyl)-N-(1-(2-(4-((2-chloro-4-(2-chloro-N-(2-oxo-2-(phenethylamino)-1-(thiophen-2-yl)ethyl)acetamido)phenoxy)methyl)-1H-1,2,3-triazol-1-yl)acetyl)piperidin-4-yl)benzamide (****GDAz-11****)*

^1^H NMR (400 MHz, CDCl_3_) δ 7.97 (s, 2H), 7.74 (d, *J* = 6.5 Hz, 2H), 7.66 (d, *J* = 8.0 Hz, 2H), 7.41 (d, *J* = 8.4 Hz, 2H), 7.15 – 7.04 (m, 6H), 6.98 (d, *J* = 7.3 Hz, 2H), 6.85 (t, *J* = 9.7 Hz, 4H), 6.78 (d, *J* = 8.3 Hz, 2H), 6.75 – 6.66 (m, 4H), 6.31 (t, *J* = 6.0 Hz, 1H), 6.01 (s, 1H), 5.32 (d, *J* = 16.3 Hz, 1H), 5.08 (dd, *J* = 16.3, 9.1 Hz, 5H), 4.41 (d, *J* = 12.9 Hz, 1H), 4.18 – 4.03 (m, 1H), 3.78 (d, *J* = 13.5 Hz, 1H), 3.70 (d, *J* = 3.1 Hz, 4H), 3.66 (s, 3H), 3.39 (dp, *J* = 30.2, 6.6 Hz, 2H), 3.15 (t, *J* = 12.5 Hz, 1H), 2.82 (s, 1H), 2.74 (s, 1H), 2.66 (q, *J* = 7.7 Hz, 2H), 2.39 (s, 2H), 1.95 (dd, *J* = 43.0, 11.7 Hz, 2H). ^13^C NMR (101 MHz, CDCl_3_) δ 167.10, 165.60, 165.08, 162.27, 161.57, 159.12, 157.58, 153.18, 143.10, 142.07, 139.49, 137.84, 137.60, 133.77, 132.72, 131.82, 131.19, 131.01, 130.73, 130.67, 129.49, 129.16, 127.76, 127.52, 127.23, 126.95, 126.90, 125.59, 125.50, 125.43, 124.74, 124.36, 123.33, 120.89, 120.62, 113.63, 112.71, 112.59, 62.01, 54.25, 54.16, 49.94, 47.10, 45.86, 43.32, 41.56, 40.05, 35.49, 34.35, 31.09, 30.40, 30.29. HRMS (ESI) calcd for [C_65_H_57_Cl_2_F_6_N_9_O_7_S + H]^+^ 1292.3461, found 1292.3446; HPLC purity: 96.921%, retention time = 15.001 min.

*4-((2-(3,5-bis(trifluoromethyl)phenyl)-4,5-bis(4-methoxyphenyl)-1H-imidazol-1-yl)methyl)-N-((1-(2-(4-((2-chloro-4-(2-chloro-N-(2-oxo-2-(phenethylamino)-1-(thiophen-2-yl)ethyl)acetamido)phenoxy)methyl)-1H-1,2,3-triazol-1-yl)acetyl)piperidin-4-yl)methyl)benzamide (****GDAz-12****)*

^1^H NMR (400 MHz, CDCl_3_) δ 7.97 (s, 2H), 7.87 (s, 1H), 7.74 (s, 2H), 7.65 (d, *J* = 8.0 Hz, 2H), 7.42 (d, *J* = 8.4 Hz, 2H), 7.17 – 7.05 (m, 6H), 7.01 (d, *J* = 7.3 Hz, 2H), 6.88 (d, *J* = 7.7 Hz, 4H), 6.80 (d, *J* = 8.3 Hz, 3H), 6.75 – 6.62 (m, 4H), 6.25 (t, *J* = 6.0 Hz, 1H), 6.01 (s, 1H), 5.15 (d, *J* = 16.1 Hz, 3H), 5.05 (s, 2H), 4.42 (d, *J* = 13.1 Hz, 1H), 3.87 – 3.69 (m, 6H), 3.67 (s, 2H), 3.40 (dd, *J* = 15.6, 8.3 Hz, 1H), 3.21 (dt, *J* = 22.6, 6.7 Hz, 2H), 3.02 (t, *J* = 12.9 Hz, 1H), 2.85 (s, 2H), 2.76 (s, 2H), 2.68 (d, *J* = 8.2 Hz, 1H), 2.54 (t, *J* = 12.9 Hz, 1H), 2.26 (s, 2H), 1.89 – 1.63 (m, 3H). ^13^C NMR (101 MHz, CDCl_3_) δ 167.05, 165.95, 165.61, 162.02, 161.58, 159.15, 157.60, 153.23, 143.10, 142.10, 139.47, 137.84, 137.61, 133.75, 132.85, 131.81, 131.19, 131.03, 130.70, 129.48, 129.15, 128.38, 127.78, 127.54, 127.24, 126.95, 126.78, 125.59, 125.45, 124.81, 124.18, 123.33, 122.15, 121.15, 120.88, 120.62, 113.65, 112.72, 112.63, 62.09, 59.67, 54.27, 54.18, 50.01, 47.10, 44.13, 44.01, 41.54, 41.34, 40.05, 35.50, 35.05, 34.38, 30.41, 29.16, 28.29. HRMS (ESI) calcd for [C_66_H_59_Cl_2_F_6_N_9_O_7_S + H]^+^ 1306.3618, found 1306.3601; HPLC purity: 95.368%, retention time = 14.990 min.

*4-((2-(3,5-bis(trifluoromethyl)phenyl)-4,5-bis(4-methoxyphenyl)-1H-imidazol-1-yl)methyl)-N-(2-(1-(2-(4-((2-chloro-4-(2-chloro-N-(2-oxo-2-(phenethylamino)-1-(thiophen-2-yl)ethyl)acetamido)phenoxy)methyl)-1H-1,2,3-triazol-1-yl)acetyl)piperidin-4-yl)ethyl)benzamide (****GDAz-13****)*

^1^H NMR (400 MHz, CDCl_3_) δ 7.97 (d, *J* = 1.6 Hz, 2H), 7.76 (d, *J* = 6.6 Hz, 2H), 7.62 (d, *J* = 8.2 Hz, 2H), 7.47 – 7.39 (m, 2H), 7.16 (t, *J* = 7.2 Hz, 3H), 7.11 (d, *J* = 8.7 Hz, 4H), 7.05 – 7.00 (m, 2H), 6.88 (d, *J* = 8.1 Hz, 3H), 6.80 (s, 3H), 6.78 – 6.74 (m, 2H), 6.72 (d, *J* = 8.9 Hz, 2H), 6.34 (t, *J* = 5.7 Hz, 1H), 6.11 (t, *J* = 5.9 Hz, 1H), 6.00 (s, 1H), 5.17 (d, *J* = 5.0 Hz, 2H), 5.05 (s, 2H), 4.44 (d, *J* = 13.6 Hz, 1H), 3.73 (d, *J* = 2.4 Hz, 5H), 3.69 (s, 3H), 3.49 – 3.36 (m, 4H), 3.08 – 2.96 (m, 1H), 2.71 (dt, *J* = 10.3, 6.9 Hz, 2H), 2.57 (d, *J* = 10.0 Hz, 1H), 1.96 (s, 1H), 1.80 (d, *J* = 13.8 Hz, 1H), 1.72 (d, *J* = 13.1 Hz, 1H), 1.58 – 1.43 (m, 3H), 1.21 – 1.03 (m, 3H). ^13^C NMR (101 MHz, CDCl_3_) δ 166.98, 165.75, 165.63, 161.87, 159.15, 157.61, 153.26, 143.11, 142.19, 139.41, 137.82, 137.58, 133.69, 133.03, 131.76, 131.19, 131.05, 130.72, 129.43, 129.14, 128.31, 127.79, 127.56, 127.29, 126.95, 126.63, 125.57, 125.48, 125.40, 124.86, 124.05, 123.32, 121.19, 120.88, 120.61, 113.65, 112.72, 112.65, 62.16, 54.28, 54.18, 50.02, 47.09, 44.46, 41.67, 41.47, 40.04, 36.42, 35.07, 34.40, 32.53, 31.28, 30.47. HRMS (ESI) calcd for [C_67_H_61_Cl_2_F_6_N_9_O_7_S + H]^+^ 1320.3774, found 1320.3751; HPLC purity: 95.084%, retention time = 15.005 min.

*N-(4-((1-(2-(4-(4-((2-(3,5-bis(trifluoromethyl)phenyl)-4,5-bis(4-methoxyphenyl)-1H-imidazol-1-yl)methyl)benzoyl)piperazin-1-yl)-2-oxoethyl)-1H-1,2,3-triazol-4-yl)methoxy)-3-chlorophenyl)-2-chloro-N-(2-oxo-2-(phenethylamino)-1-(thiophen-2-yl)ethyl)acetamide (****GDAz-14****)*

^1^H NMR (400 MHz, CDCl_3_) δ 7.96 (d, *J* = 1.7 Hz, 2H), 7.77 (s, 1H), 7.74 (s, 1H), 7.46 – 7.41 (m, 2H), 7.27 (d, *J* = 8.0 Hz, 2H), 7.20 (s, 1H), 7.16 – 7.11 (m, 5H), 7.10 – 7.07 (m, 1H), 7.04 – 6.99 (m, 2H), 6.94 (d, *J* = 7.9 Hz, 3H), 6.85 – 6.80 (m, 2H), 6.77 – 6.68 (m, 4H), 6.24 (t, *J* = 5.9 Hz, 1H), 6.03 (s, 1H), 5.27 – 4.99 (m, 6H), 3.73 (d, *J* = 3.9 Hz, 6H), 3.68 (s, 4H), 3.60 – 3.24 (m, 8H), 2.80 (d, *J* = 31.7 Hz, 1H), 2.71 – 2.65 (m, 1H). ^13^C NMR (101 MHz, CDCl_3_) δ 168.87, 167.03, 165.59, 162.71, 159.19, 157.62, 153.20, 143.04, 142.33, 138.41, 137.85, 137.64, 133.76, 133.39, 131.82, 131.15, 130.94, 130.73, 130.60, 129.56, 129.13, 128.46, 127.79, 127.53, 127.23, 126.99, 126.89, 125.58, 125.44, 125.00, 124.11, 123.37, 120.87, 120.66, 113.72, 112.73, 62.10, 59.56, 54.29, 54.18, 49.91, 47.07, 41.55, 40.03, 34.39. HRMS (ESI) calcd for [C_64_H_55_Cl_2_F_6_N_9_O_7_S + H]^+^ 1278.3305, found 1278.3286; HPLC purity: 98.805%, retention time = 14.896 min.

**Scheme S2.** Synthesis of GDAz-Neg1

*N-(4-((1-(2-(2-(2-(2-aminoethoxy)ethoxy)ethoxy)ethyl)-1H-1,2,3-triazol-4-yl)methoxy)-3-chlorophenyl)-2-chloro-N-(2-oxo-2-(phenethylamino)-1-(thiophen-2-yl)ethyl)acetamide (****GDAz-Neg1****)*: To an aqueous solution of sodium ascorbate (679 mg, 3.43 mmol) and copper sulfate pentahydrate (109 mg 0.68 mmol) was added a mixture solution (*t*-BuOH:DMF:THF:H_2_O = 2/2/2/1) of ML162-yne (**5**, 759 mg 1.51 mmol) and 2-(2-(2-(2-azidoethoxy)ethoxy)ethoxy)ethan-1-amine (300 mg 1.37 mmol) at 0 °C, and the resulting mixture was stirred under a nitrogen atmosphere for overnight. The reaction solution was diluted with water, extracted with DCM, washed with brine, dried over Na_2_SO_4_, filtered, and concentrated. The residue was purified by flash column chromatography (DCM: MeOH = 15:1) and dried to yield the title compound 534 mg (53%). ^1^H NMR (400 MHz, CDCl_3_) δ 7.86 (s, 1H), 7.15 (dd, *J* = 19.2, 6.6 Hz, 5H), 7.06 (d, *J* = 7.2 Hz, 3H), 6.84 – 6.71 (m, 3H), 6.00 (s, 1H), 5.18 (s, 2H), 4.51 (s, 2H), 3.83 (d, *J* = 5.1 Hz, 1H), 3.75 (s, 2H), 3.54 (p, *J* = 10.1 Hz, 15H), 2.85 (d, *J* = 31.1 Hz, 1H), 2.75 – 2.71 (m, 1H). ^13^C NMR (101 MHz, CDCl_3_) δ 166.98, 165.69, 153.30, 137.58, 133.70, 130.76, 130.50, 129.15, 128.35, 127.81, 127.58, 127.32, 125.58, 125.50, 112.64, 71.43, 69.47, 69.46, 68.30, 62.21, 49.39, 41.38, 40.08, 34.43, 28.67. HRMS (ESI) calcd for [C_33_H_40_Cl_2_N_6_O_6_S + H]^+^ 719.2185, found 719.2173;

**Scheme S3.** Synthesis of GDAz-Neg2

***Reagents and conditions***: a) CH_3_COONH_4_, CH_3_COOH, 100 ℃, 56%; b) EDCI/HOBT, DMF, rt; c) sodium ascorbate, CuSO_4_, *t*-BuOH:DMF:THF:H_2_O (V/V/V = 2/2/2/1), 0 ℃~ rt, 41%.

*4-((4,5-bis(4-methoxyphenyl)-2-(pyridin-4-yl)-1H-imidazol-1-yl)methyl)benzoic acid (****13****)*: A solution of 1,2-bis(4-methoxyphenyl)ethane-1,2-dione (500 mg, 1.85 mmol), isonicotinaldehyde (420 mg, 2.77 mmol), 4,4-dimethoxybenzil (298 mg, 2.77 mmol) and ammonium acetate (855 mg, 11 mmol) in acetic acid was stirred for 12 h at 100 °C. After cooling, the mixture was diluted with ethyl acetate, and washed with water, saturated NaHCO_3_ and brine. The organic layer was dried with anhydrous Na_2_SO_4_, filtered and concentrated under reduced pressure. The residue was purified by flash column chromatography (DCM: MeOH = 15:1) and dried to yield the goal compound **13** as a yellow solid (510 mg, 56%).

The other procedures for preparation of compound ***GDAz-Neg2*** were similar to the related synthetic method described above.

*4-((4,5-bis(4-methoxyphenyl)-2-(pyridin-4-yl)-1H-imidazol-1-yl)methyl)-N-(2-(2-(2-(2-(4-((2-chloro-4-(2-chloro-N-(2-oxo-2-(phenethylamino)-1-(thiophen-2-yl)ethyl)acetamido)phenoxy)methyl)-1H-1,2,3-triazol-1-yl)ethoxy)ethoxy)ethoxy)ethyl)benzamide (****GDAz-Neg2****)*

^1^H NMR (400 MHz, CDCl_3_) δ 8.48 (d, *J* = 4.9 Hz, 2H), 7.75 (d, *J* = 2.6 Hz, 1H), 7.70 – 7.64 (m, 2H), 7.50 – 7.44 (m, 2H), 7.44 – 7.39 (m, 2H), 7.18 – 7.13 (m, 3H), 7.12 – 7.08 (m, 1H), 7.06 – 7.00 (m, 5H), 6.87 (d, *J* = 8.3 Hz, 2H), 6.80 – 6.74 (m, 4H), 6.71 – 6.67 (m, 2H), 6.26 (t, *J* = 5.9 Hz, 1H), 6.01 (s, 1H), 5.10 (d, *J* = 3.9 Hz, 4H), 4.37 (t, *J* = 5.0 Hz, 2H), 3.75 – 3.70 (m, 7H), 3.68 (s, 3H), 3.58 – 3.52 (m, 6H), 3.50 (d, *J* = 13.4 Hz, 8H), 2.76 – 2.62 (m, 2H). ^13^C NMR (101 MHz, CDCl_3_) δ 167.04, 165.84, 165.61, 159.05, 157.52, 153.24, 149.06, 143.27, 141.89, 139.57, 138.03, 137.63, 137.11, 133.78, 132.98, 131.18, 129.55, 129.11, 128.44, 127.80, 127.55, 127.24, 126.91, 126.86, 125.63, 125.57, 125.45, 124.70, 123.30, 121.38, 120.97, 113.53, 112.67, 112.52, 69.43, 69.29, 69.08, 68.80, 68.27, 62.14, 54.26, 54.18, 49.25, 47.04, 41.42, 40.06, 38.76, 34.41. HRMS (ESI) calcd for [C_63_H_63_Cl_2_N_9_O_9_S + H]^+^ 1192.3925, found 1192.3901; HPLC purity: 97.451%, retention time = 14.621 min.

**Scheme S4.** Synthesis of GDAz-OH

To a stirred solution of **GDAz-3** (50 mg) in THF (2 mL), 20% sodium hydroxide aqueous solution (2 mL) was added. After stirring for 48 h at room temperature, the reaction solution was extracted with EA, and the concentrated residue was purified by silica gel column chromatography (silica plate). Due to the extremely small mass of the synthesized compound, only high-resolution mass spectrometry data was provided here. MS (ESI) calcd for [C_66_H_63_ClF_6_N_8_O_10_S + H]^+^ 1309.41, found 1309.50

**Scheme S5.** Synthesis of GDAz-biotin

***Reagents and conditions***: a) HATU/DIPEA, DMF, rt.; b) CH_3_COONH_4_, CH_3_COOH, 100 ℃, 52%; c) sodium ascorbate, CuSO_4_, *t*-BuOH: DMF: THF: H_2_O (V/V/V = 2/2/2/1), 0 ℃~ rt, ~15%; d) EDCI/HOBT, DMF, rt.

The target compound **GDAz-biotin** was obtained according to the related synthetic methods described above. Due to the extremely small mass of the synthesized compound, only high-resolution mass spectrometry data was provided here. HRMS (ESI) calcd for [C_85_H_98_Cl_2_N_14_O_15_S_2_ + H]^+^ 1689.6233, found 1689.6229.

**Scheme S6. Synthesis of GDAz-15~17**

***Reagents and conditions****:* a) TFA, DCM, rt.; b) HATU/DIPEA, DMF, rt.; c) TFA, DCM, rt.; d) HATU/DIPEA, DMF, rt.

*(S)-2-(4-(4-chlorophenyl)-2,3,9-trimethyl-6H-thieno[3,2-f][1,2,4]triazolo[4,3-a][1,4]diazepin-6-yl)acetic acid (****19****):* The JQ1 (**18**, 1 g, 2.2 mmol) was dissolved in 40% TFA/DCM solution (24 mL) and stirred at room temperature for 5 h. The reaction mixture was then concentrated and dissolved in ethyl acetate (EtOAc). The organic layer was washed with water and brine, dried over Na_2_SO_4_, and concentrated under vacuum. The residue was purified by flash column chromatography to give the compound **19** (790 mg, 90%) as a yellow solid.

The other procedures for preparation of compounds **GDAz-15~17** were similar to the related synthetic method described above.

*(S)-4-((2-(3,5-bis(trifluoromethyl)phenyl)-4,5-bis(4-methoxyphenyl)-1H-imidazol-1-yl)methyl)-N-(2-(2-(2-(2-(4-(4-chlorophenyl)-2,3,9-trimethyl-6H-thieno[3,2-f][1,2,4]triazolo[4,3-a][1,4]diazepin-6-yl)acetamido)ethoxy)ethoxy)ethyl)benzamide (****GDAz-15****)*

^1^H NMR (400 MHz, CDCl_3_) δ 7.97 (s, 2H), 7.81 – 7.70 (m, 3H), 7.65 (d, *J* = 5.3 Hz, 1H), 7.43 (d, *J* = 8.8 Hz, 2H), 7.28 (d, *J* = 8.0 Hz, 3H), 7.10 (d, *J* = 8.3 Hz, 2H), 6.93 – 6.67 (m, 6H), 4.95 (q, *J* = 17.3 Hz, 2H), 4.56 (dd, *J* = 8.7, 5.5 Hz, 1H), 3.72 (d, *J* = 11.7 Hz, 6H), 3.65 – 3.56 (m, 7H), 3.55 – 3.29 (m, 5H), 3.20 (dd, *J* = 13.7, 5.0 Hz, 1H), 2.41 (d, *J* = 74.7 Hz, 10H). ^13^C NMR (101 MHz, CDCl_3_) δ 169.38, 165.69, 163.06, 159.36, 157.94, 154.54, 142.60, 138.43, 135.86, 135.48, 133.23, 131.36, 131.27, 131.11, 130.98, 130.78, 130.10, 129.93, 129.52, 129.46, 128.84, 127.94, 127.69, 127.27, 127.10, 124.70, 123.22, 121.73, 120.51, 113.74, 112.83, 69.30, 69.24, 69.11, 68.81, 54.29, 54.19, 53.48, 47.33, 38.91, 38.33, 38.07, 13.34, 12.06, 10.65. HRMS (ESI) calcd for [C_58_H_53_ClF_6_N_8_O_6_S + H]^+^ 1139.3480, found 1139.3458; HPLC purity: 96.074%, retention time = 15.310 min.

*(S)-4-((2-(3,5-bis(trifluoromethyl)phenyl)-4,5-bis(4-methoxyphenyl)-1H-imidazol-1-yl)methyl)-N-(1-(4-(4-chlorophenyl)-2,3,9-trimethyl-6H-thieno[3,2-f][1,2,4]triazolo[4,3-a][1,4]diazepin-6-yl)-2-oxo-6,9,12-trioxa-3-azatetradecan-14-yl)benzamide (****GDAz-16****)*

^1^H NMR (400 MHz, CDCl_3_) δ 8.00 (s, 2H), 7.76 – 7.71 (m, 2H), 7.43 (d, *J* = 8.5 Hz, 2H), 7.30 (d, *J* = 8.1 Hz, 3H), 7.24 (d, *J* = 12.9 Hz, 2H), 7.10 (d, *J* = 8.4 Hz, 2H), 6.86 (d, *J* = 8.1 Hz, 2H), 6.81 (d, *J* = 8.3 Hz, 2H), 6.72 (d, *J* = 8.5 Hz, 2H), 5.03 (s, 2H), 4.58 (t, *J* = 7.0 Hz, 1H), 3.72 (d, *J* = 16.0 Hz, 5H), 3.58 (d, *J* = 17.3 Hz, 13H), 3.48 – 3.43 (m, 2H), 3.35 (t, *J* = 5.2 Hz, 2H), 3.26 (dd, *J* = 14.6, 6.7 Hz, 1H), 2.56 (d, *J* = 24.2 Hz, 6H), 2.32 (s, 4H). ^13^C NMR (101 MHz, CDCl_3_) δ 169.48, 165.80, 162.99, 159.11, 157.57, 154.61, 148.85, 143.10, 139.04, 137.80, 135.76, 135.52, 133.08, 131.74, 131.20, 130.94, 130.72, 129.92, 129.48, 128.84, 128.82, 127.67, 127.53, 127.05, 126.97, 125.49, 124.57, 123.33, 120.93, 113.62, 112.69, 69.40, 69.10, 68.80, 68.75, 54.26, 54.17, 53.33, 47.16, 38.80, 38.30, 37.81, 13.36, 12.06, 10.68. HRMS (ESI) calcd for [C_60_H_57_ClF_6_N_8_O_7_S + H]^+^ 1183.3742, found 1183.3724; HPLC purity: 95.122%, retention time = 15.336 min.

*(S)-4-((2-(3,5-bis(trifluoromethyl)phenyl)-4,5-bis(4-methoxyphenyl)-1H-imidazol-1-yl)methyl)-N-(1-(4-(4-chlorophenyl)-2,3,9-trimethyl-6H-thieno[3,2-f][1,2,4]triazolo[4,3-a][1,4]diazepin-6-yl)-2-oxo-6,9,12,15-tetraoxa-3-azaheptadecan-17-yl)benzamide (****GDAz-17****)*

^1^H NMR (400 MHz, CDCl_3_) δ 8.01 (s, 2H), 7.78 – 7.73 (m, 3H), 7.71 – 7.67 (m, 1H), 7.43 (d, *J* = 8.8 Hz, 2H), 7.30 (d, *J* = 8.3 Hz, 2H), 7.22 (s, 1H), 7.09 (d, *J* = 8.5 Hz, 2H), 6.83 (dd, *J* = 17.4, 8.3 Hz, 4H), 6.72 (d, *J* = 8.8 Hz, 2H), 5.05 (s, 2H), 4.60 (t, *J* = 6.9 Hz, 1H), 3.73 (s, 3H), 3.69 (s, 3H), 3.56 (d, *J* = 7.3 Hz, 12H), 3.52 (s, 4H), 3.45 (d, *J* = 4.8 Hz, 2H), 3.36 (dd, *J* = 10.8, 5.0 Hz, 4H), 2.84 (s, 3H), 2.52 (s, 3H), 2.31 (s, 3H). ^13^C NMR (101 MHz, CDCl_3_) δ 169.59, 165.83, 162.79, 159.13, 157.57, 154.74, 148.80, 143.09, 138.97, 137.79, 135.68, 135.55, 133.13, 131.76, 131.19, 130.74, 129.94, 129.89, 129.57, 129.50, 128.87, 127.62, 127.53, 127.13, 126.94, 125.48, 124.52, 123.33, 120.91, 113.63, 112.69, 69.42, 69.36, 69.28, 69.19, 69.07, 69.01, 68.87, 68.83, 54.26, 54.17, 53.15, 47.17, 38.46, 38.22, 37.45, 13.37, 12.06, 10.67. HRMS (ESI) calcd for [C_62_H_61_ClF_6_N_8_O_8_S + H]^+^ 1227.4004, found 1227.3993; HPLC purity: 97.357%, retention time = 15.359 min.

**^1^H-NMR, and ^13^C-NMR-Spectra for target compounds**

GDAz-1


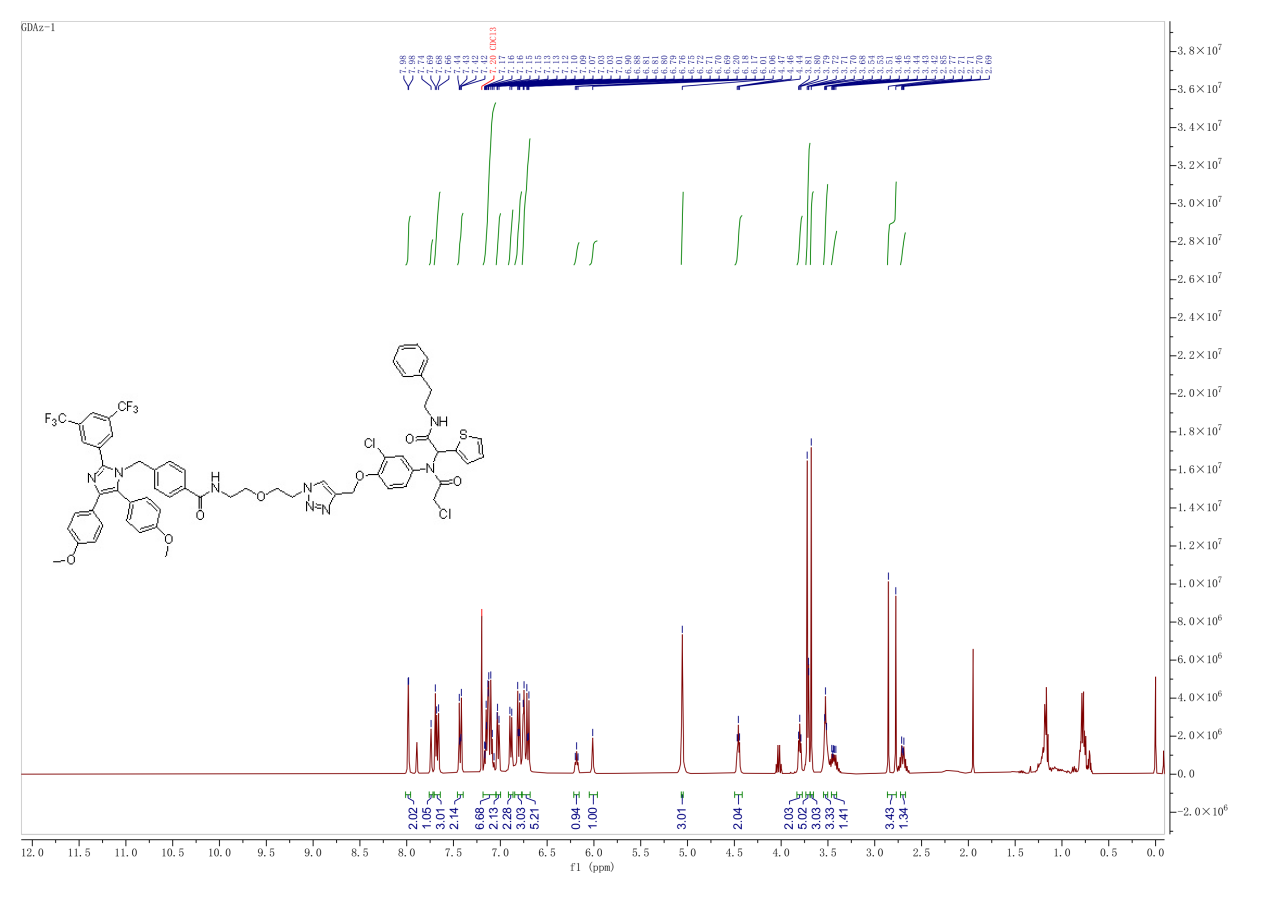


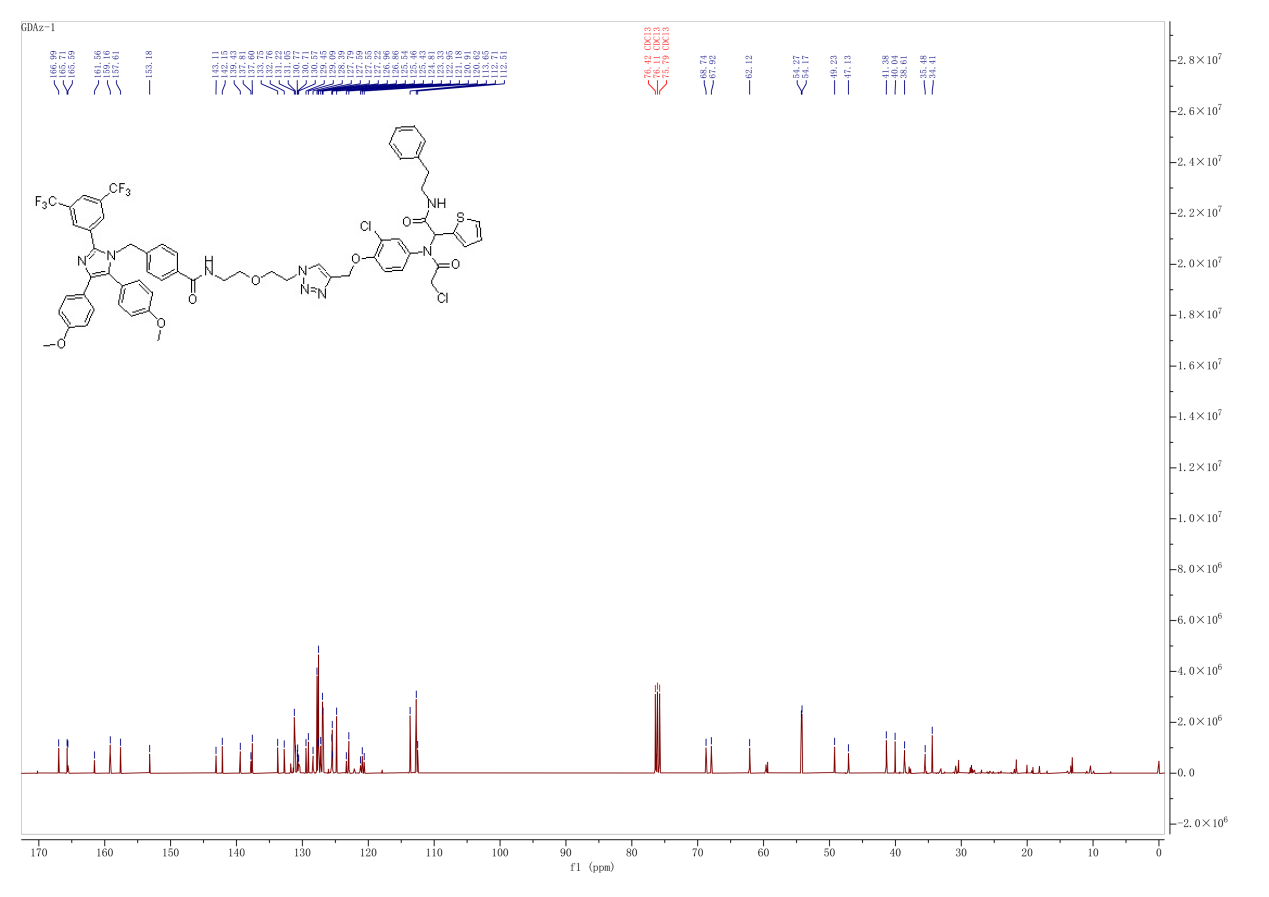


GDAz-2


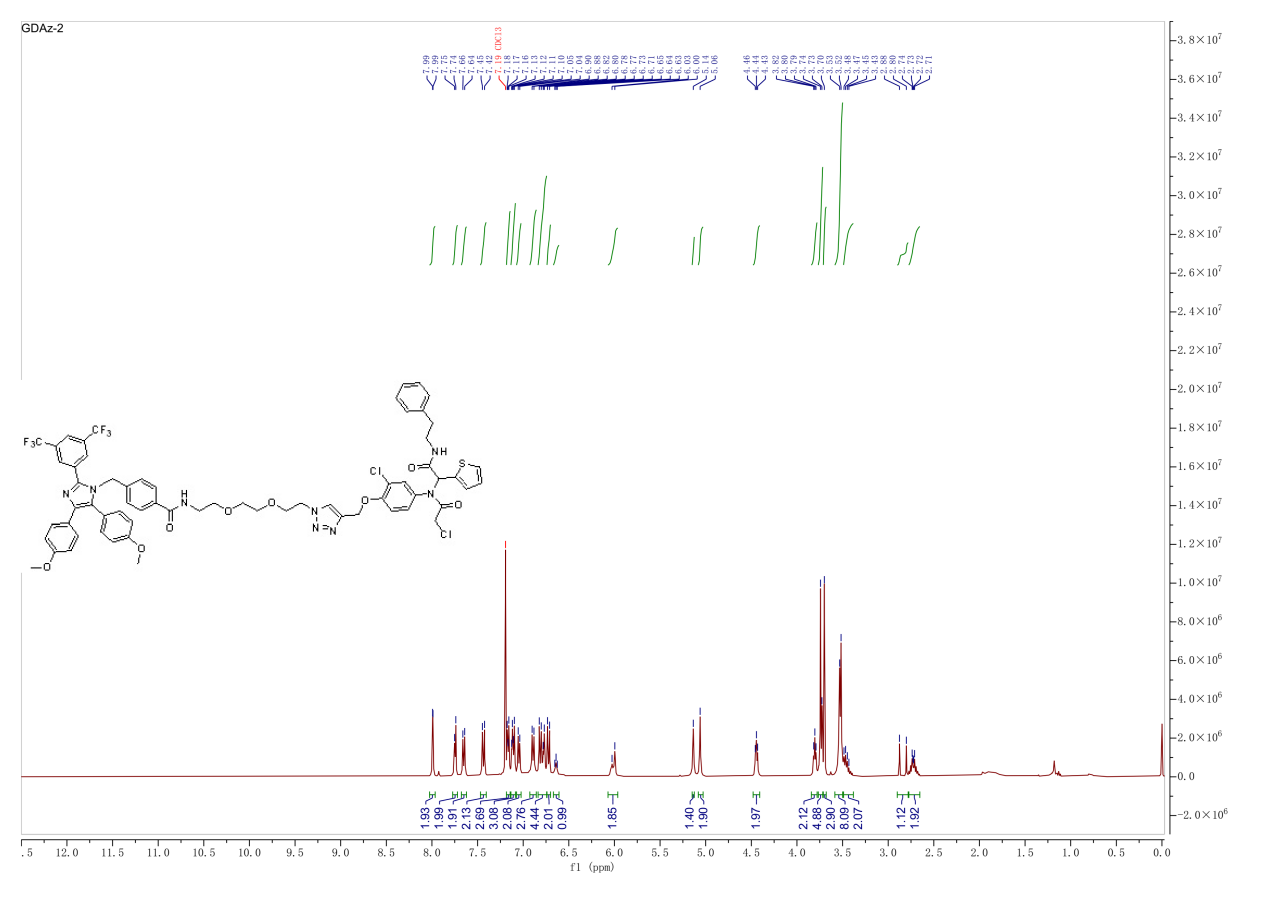


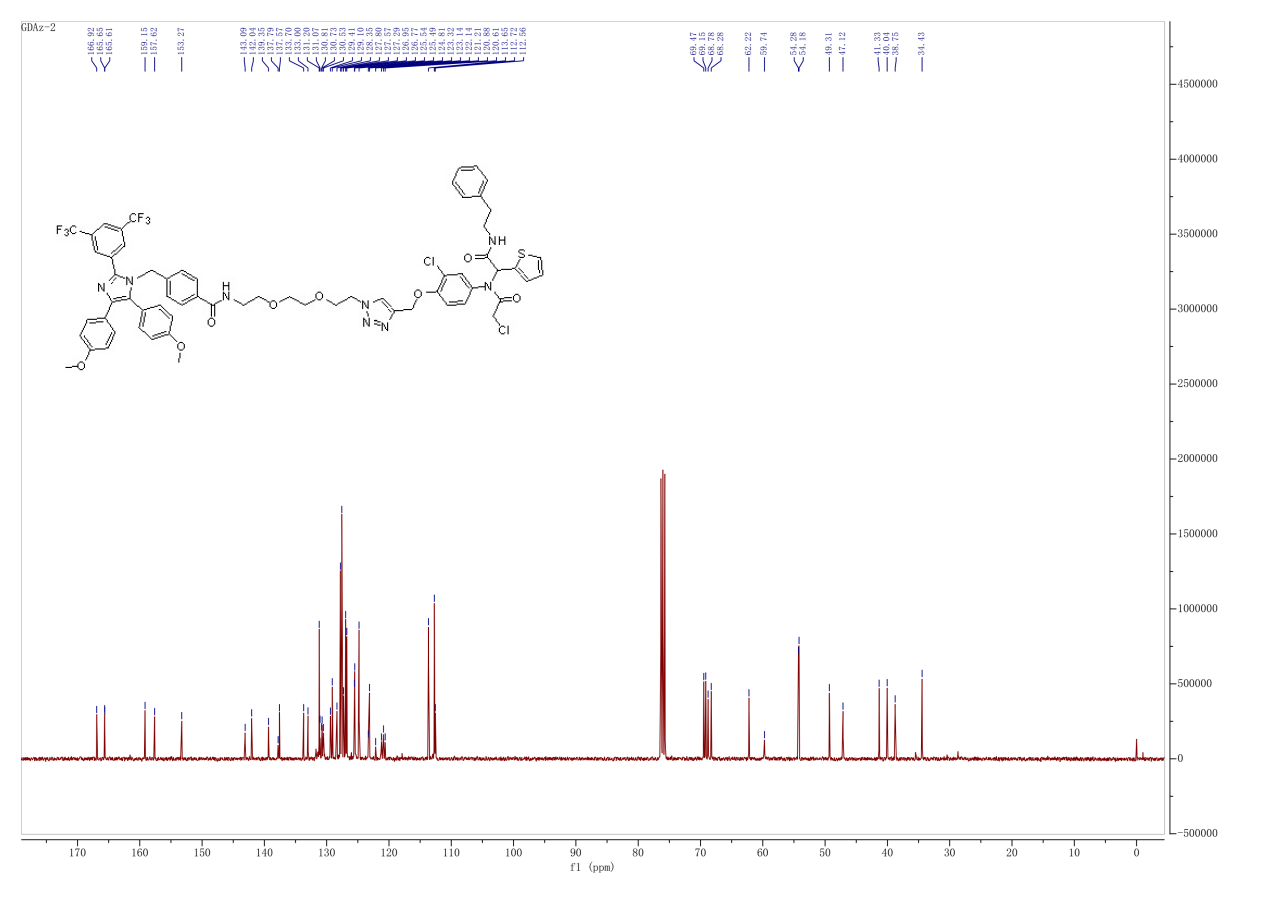


GDAz-3


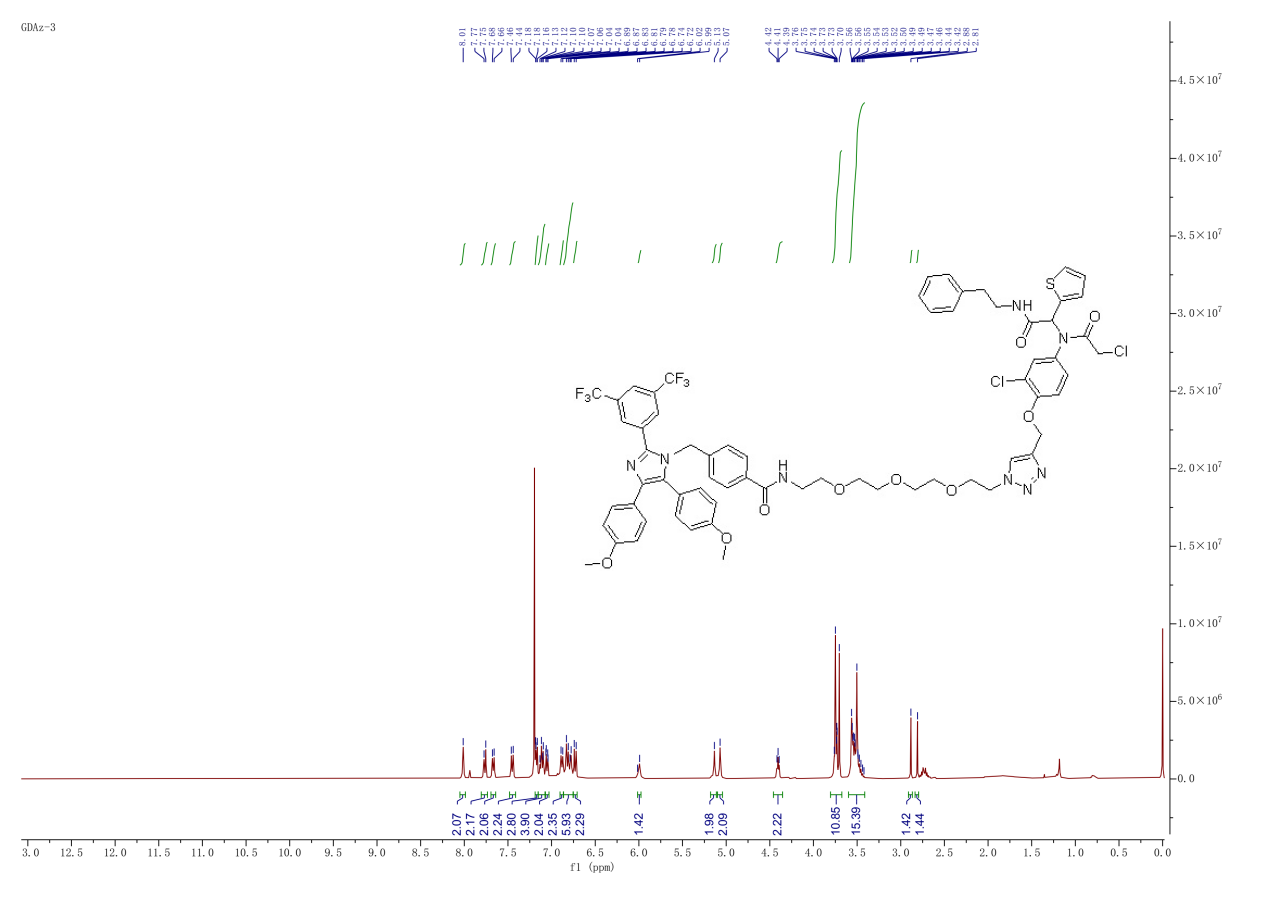


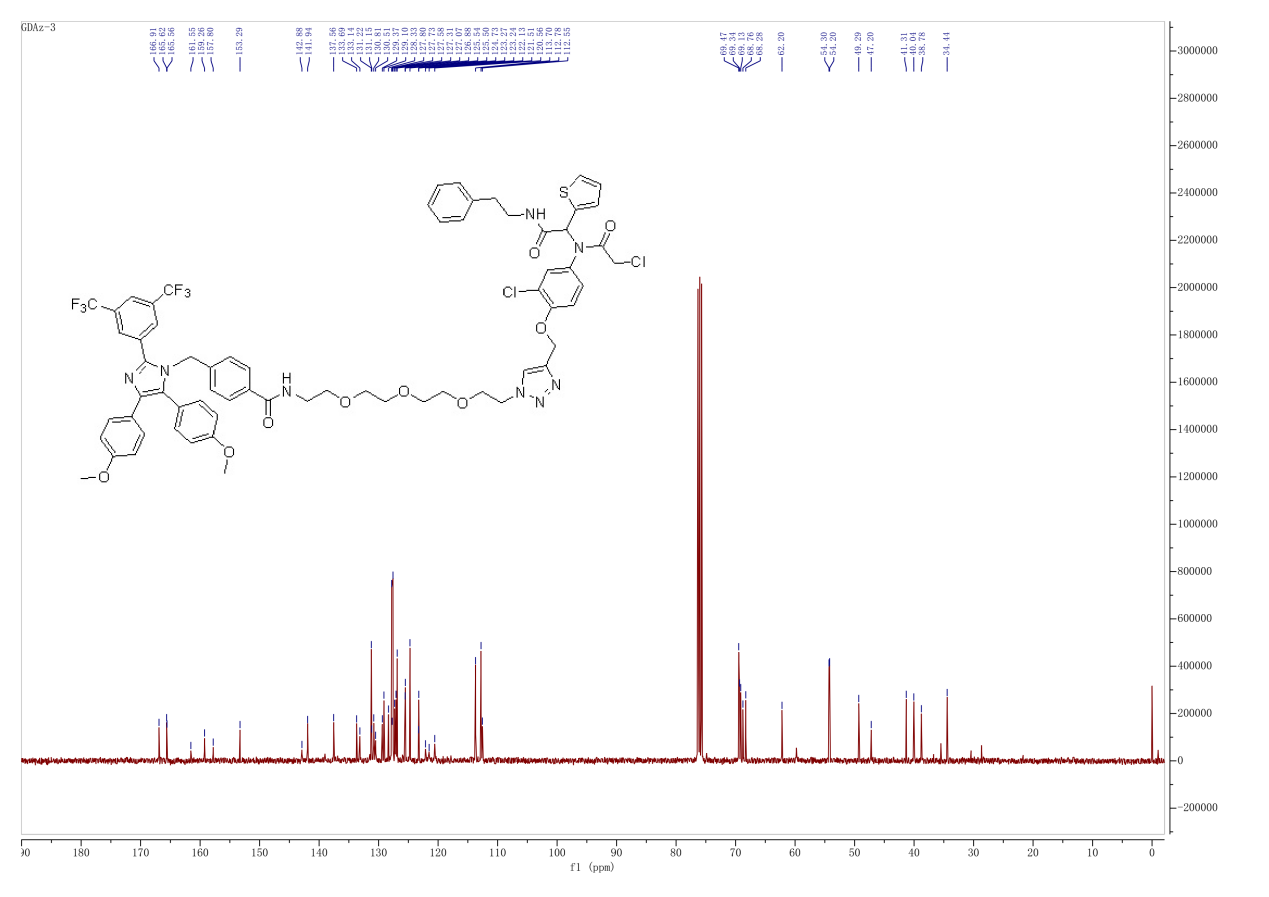


GDAz-4


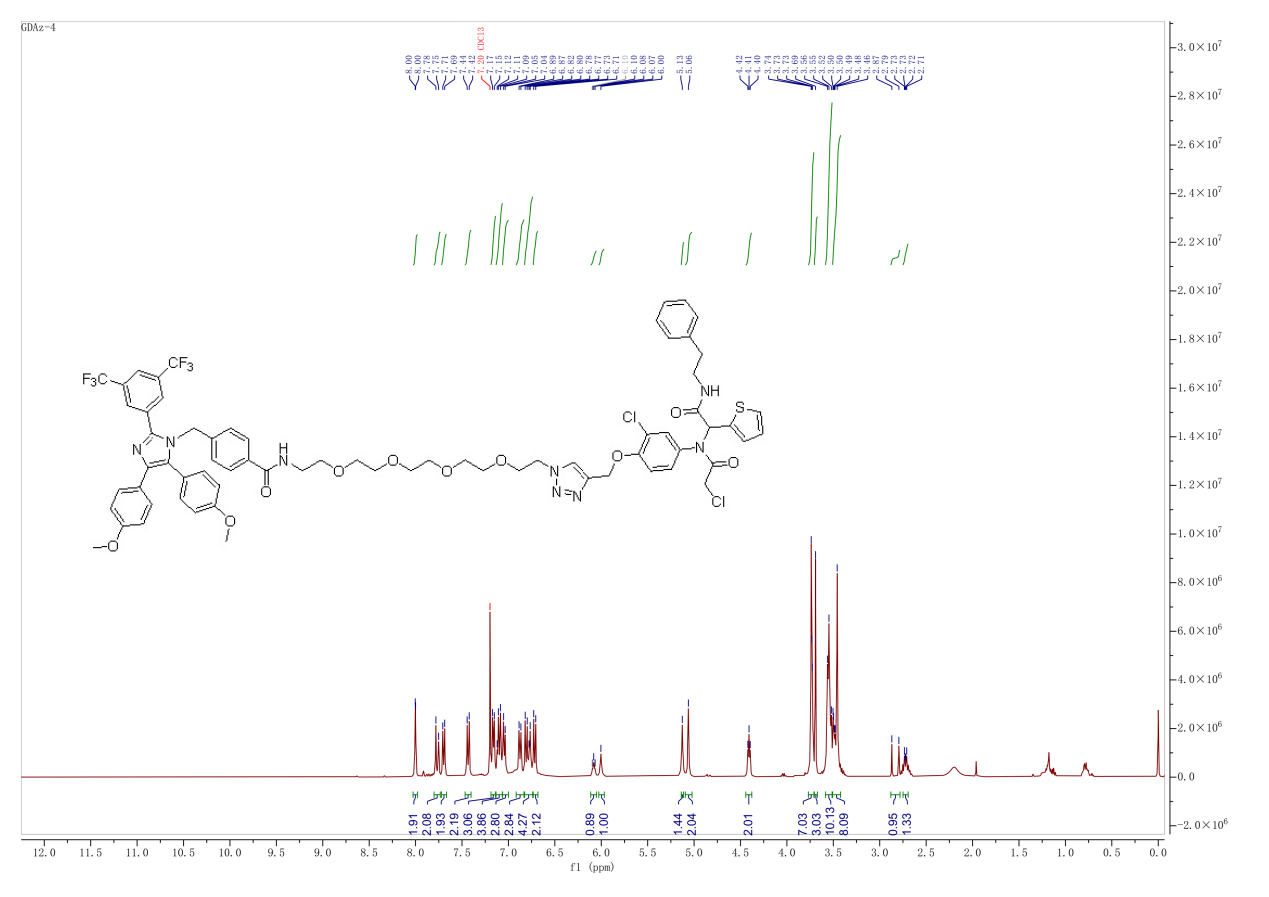


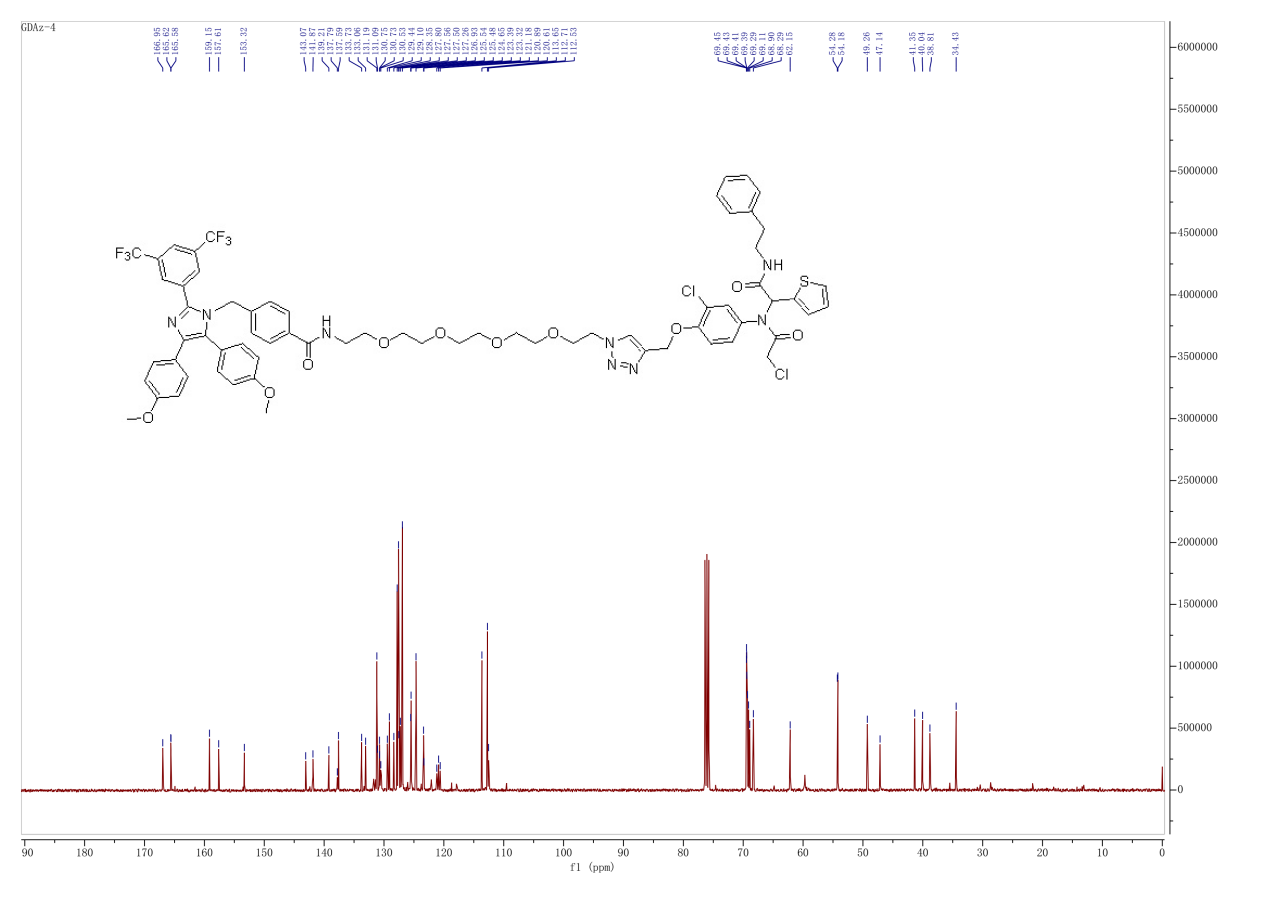


GDAz-5


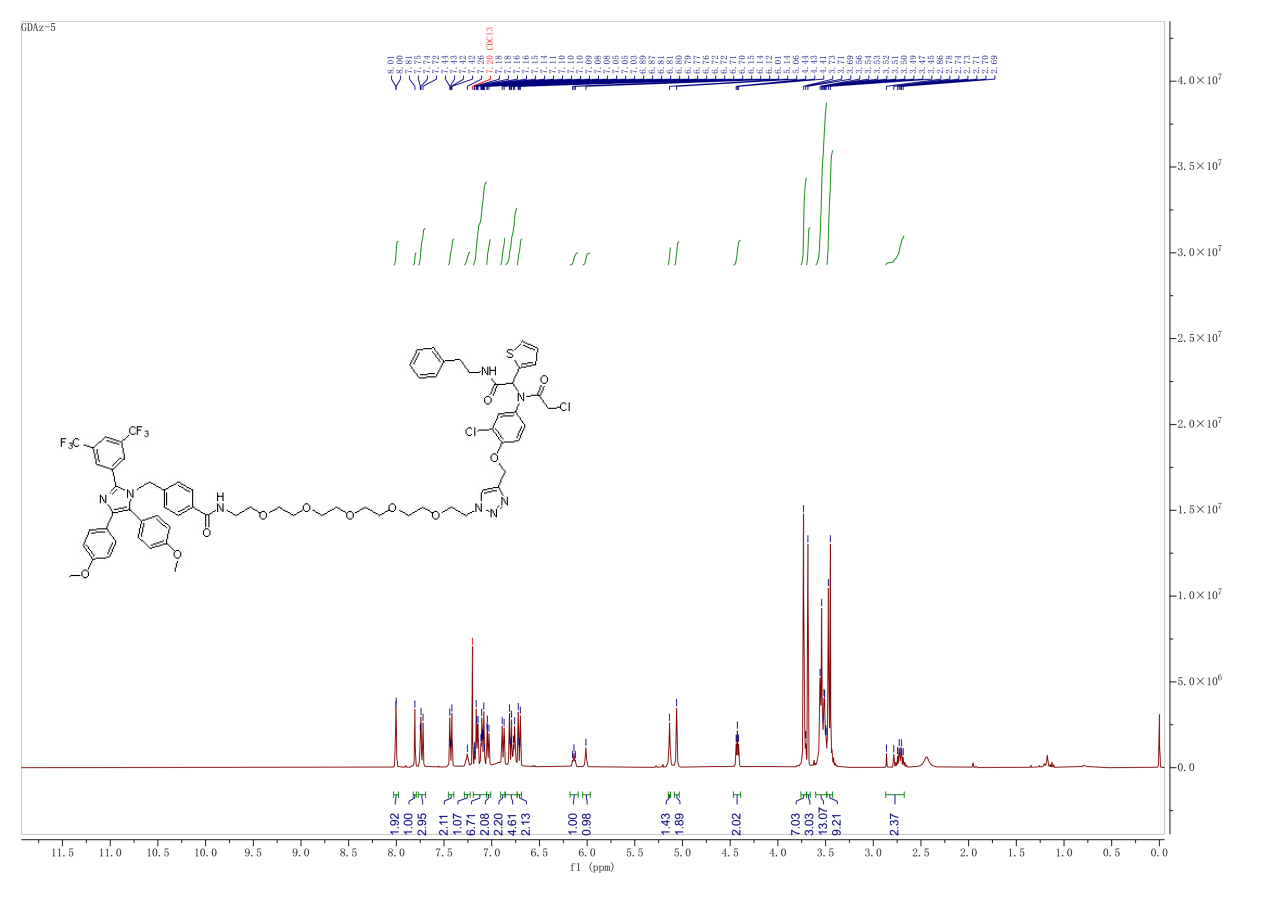


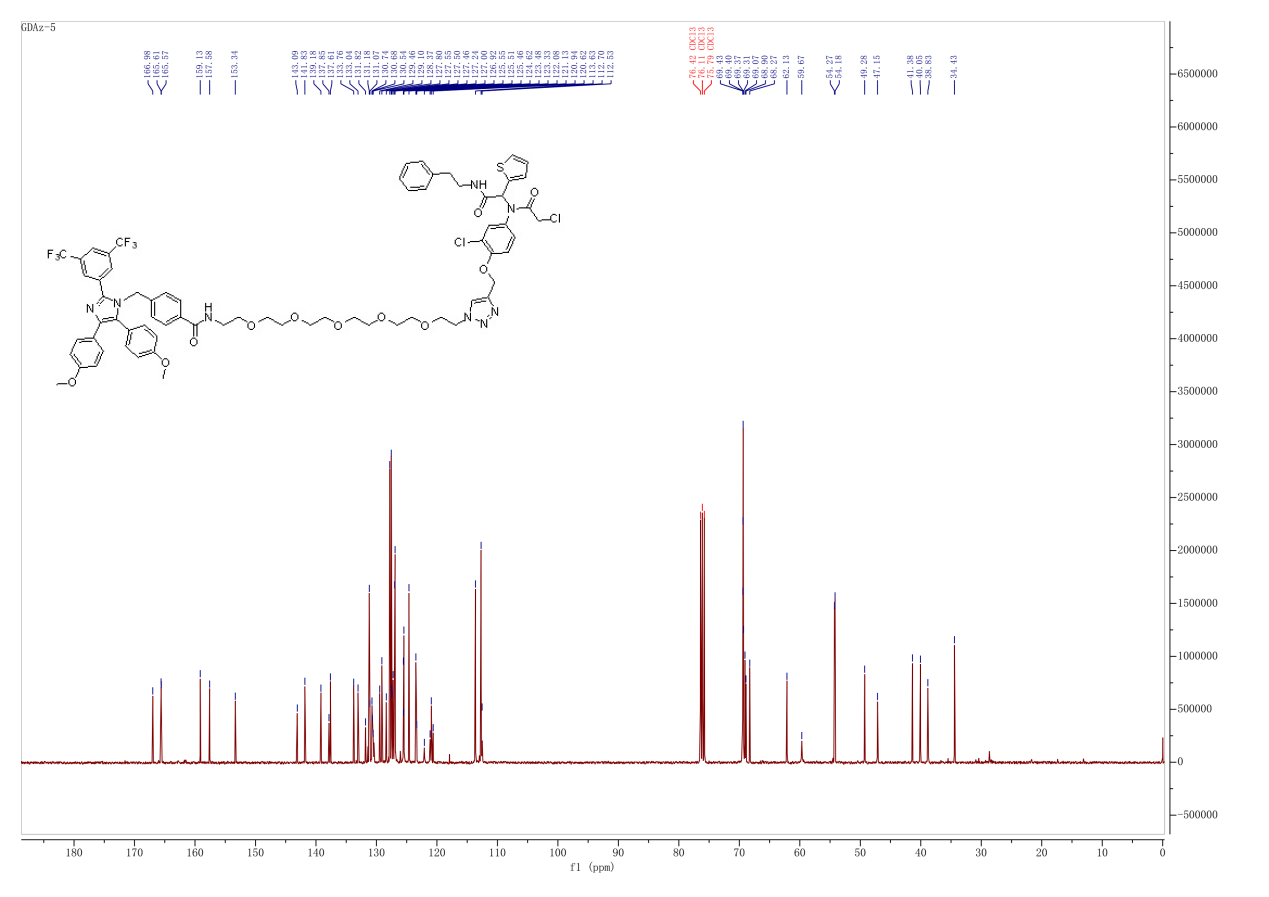


GDAz-6


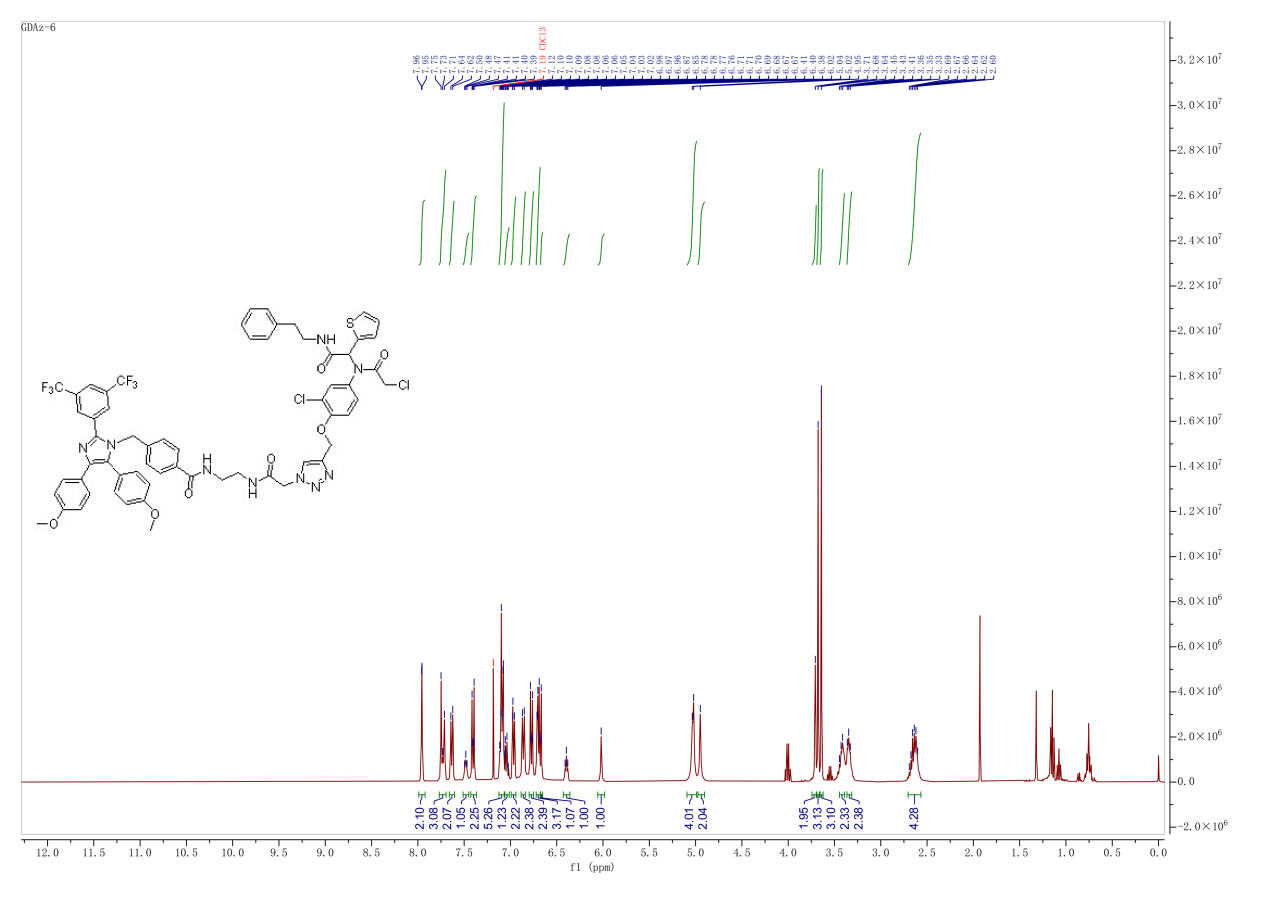


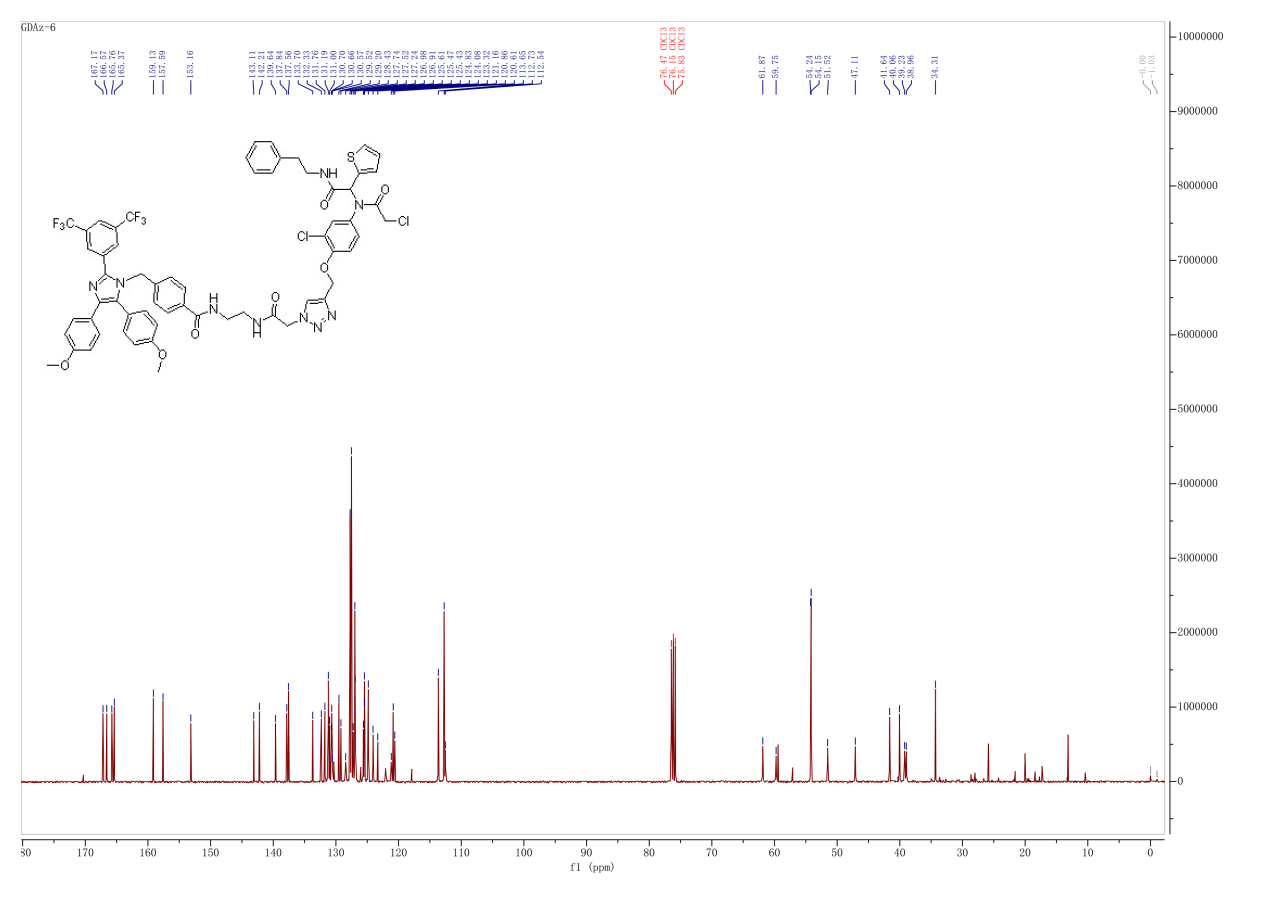


GDAz-7


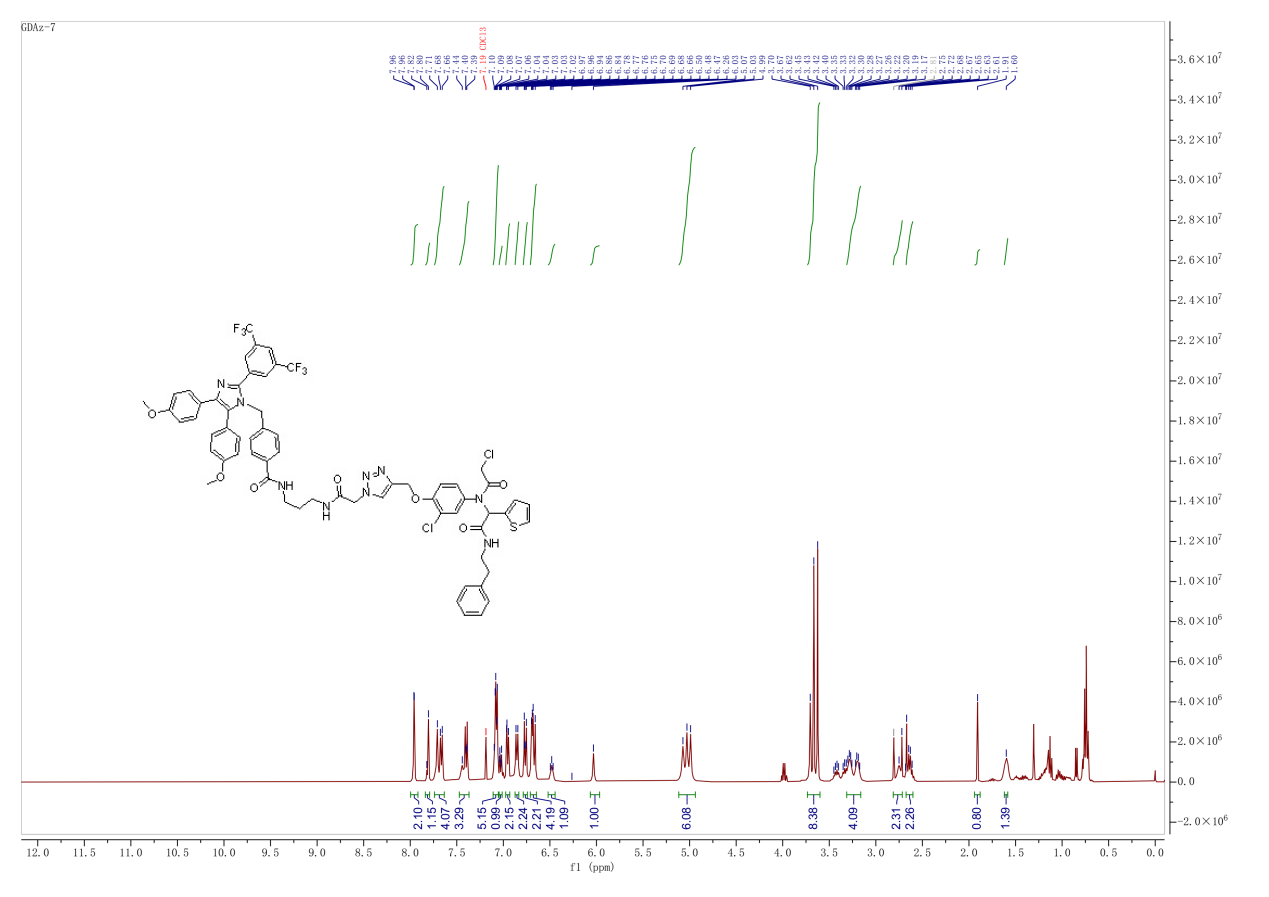


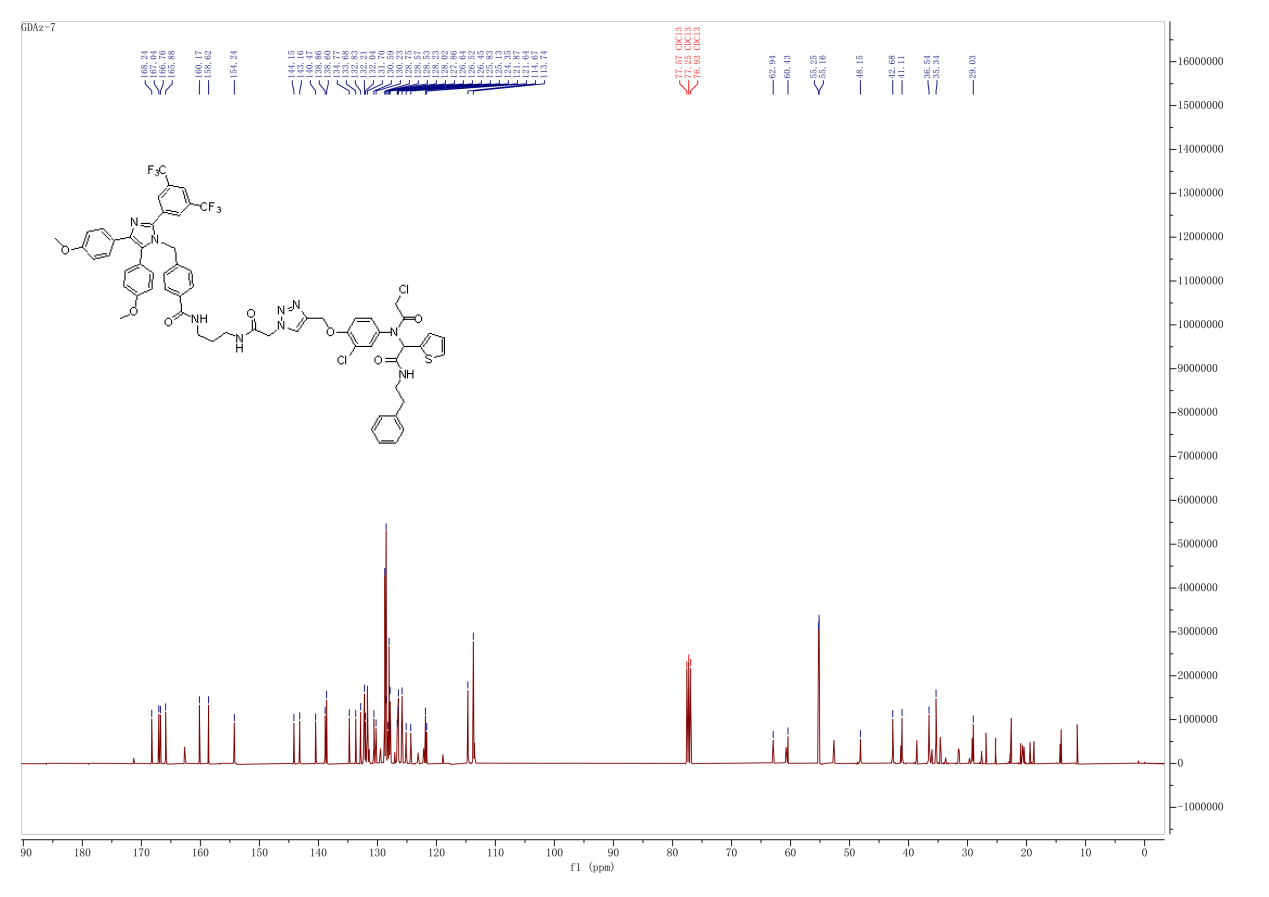


GDAz-8


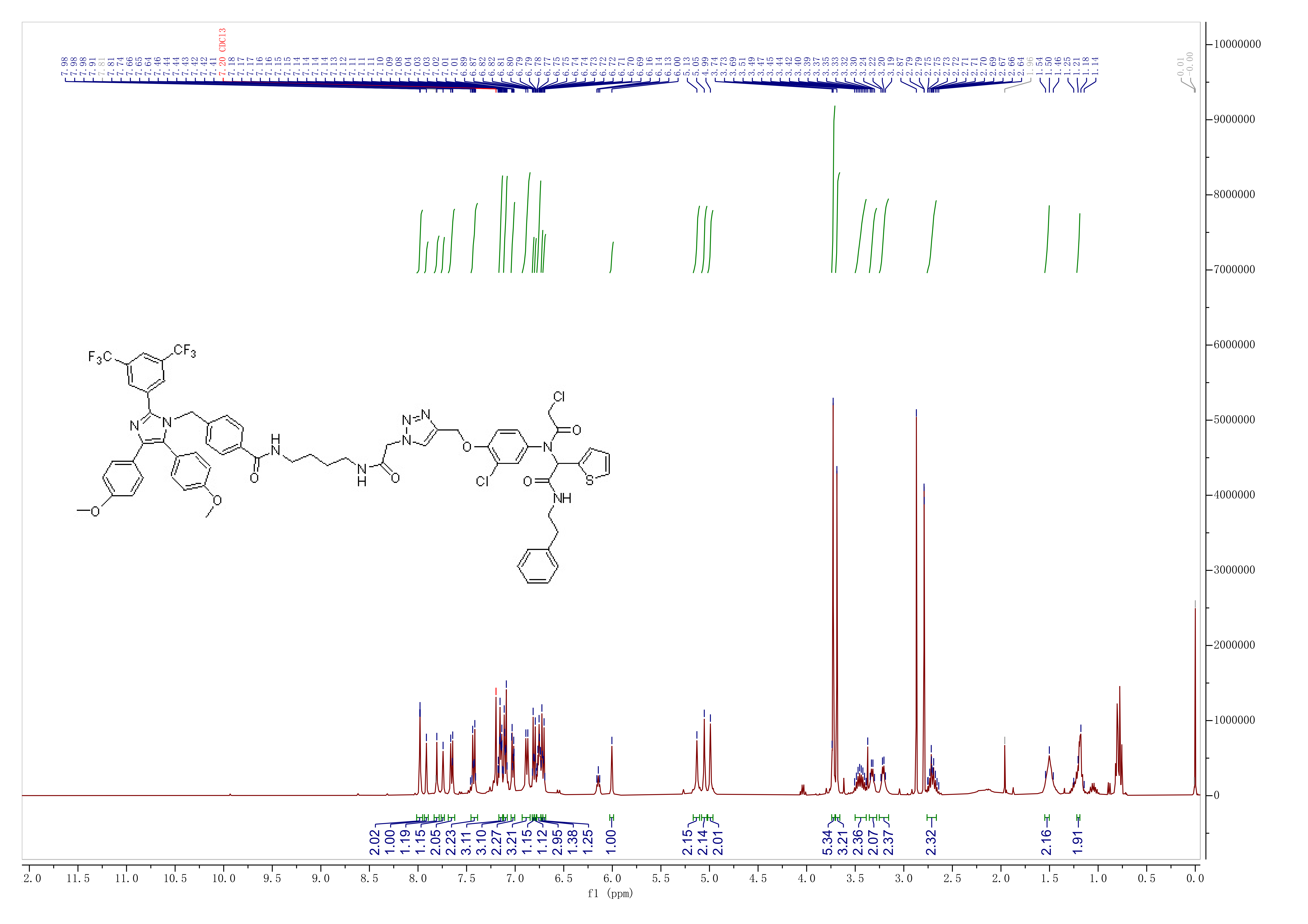


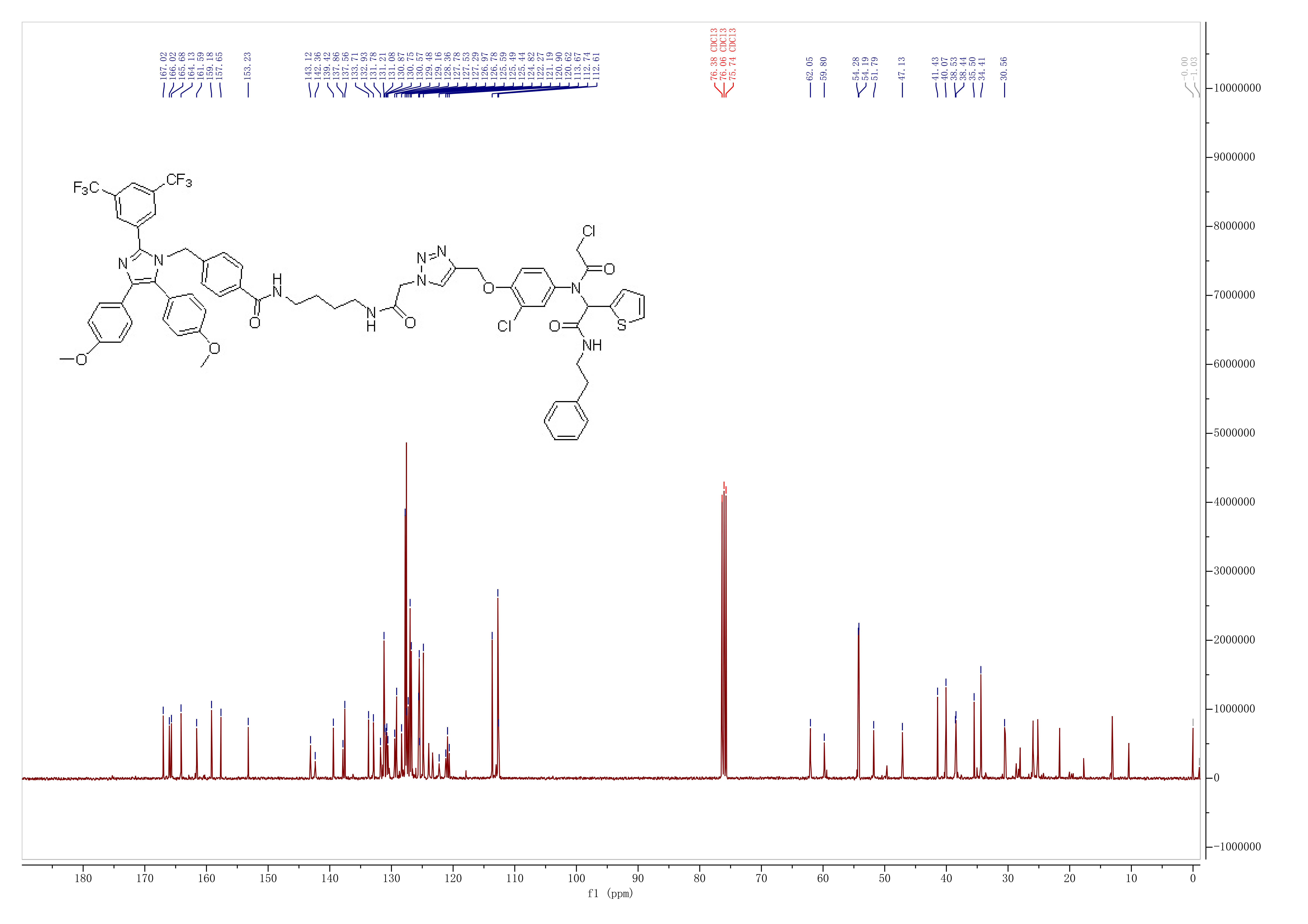


GDAz-9


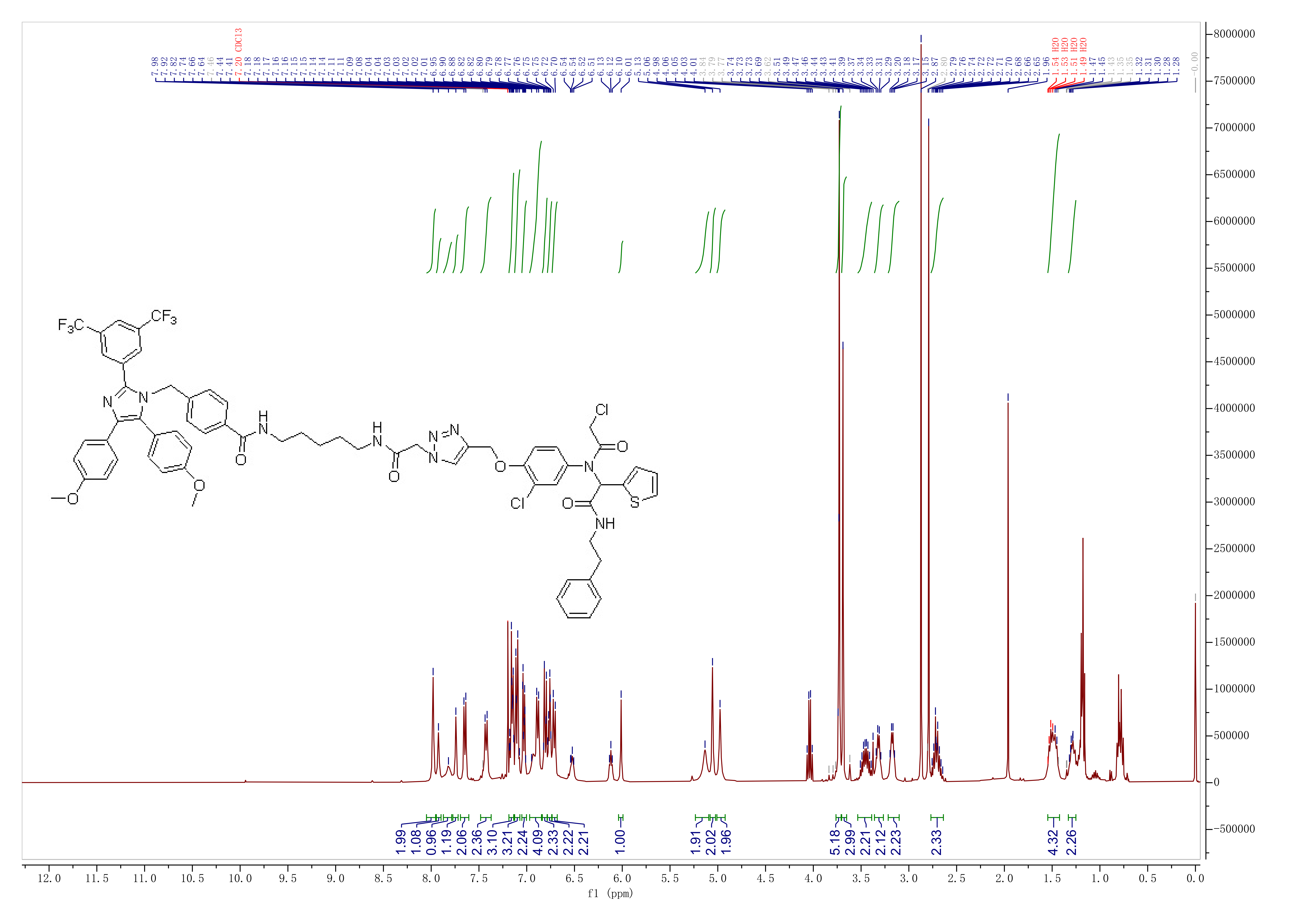


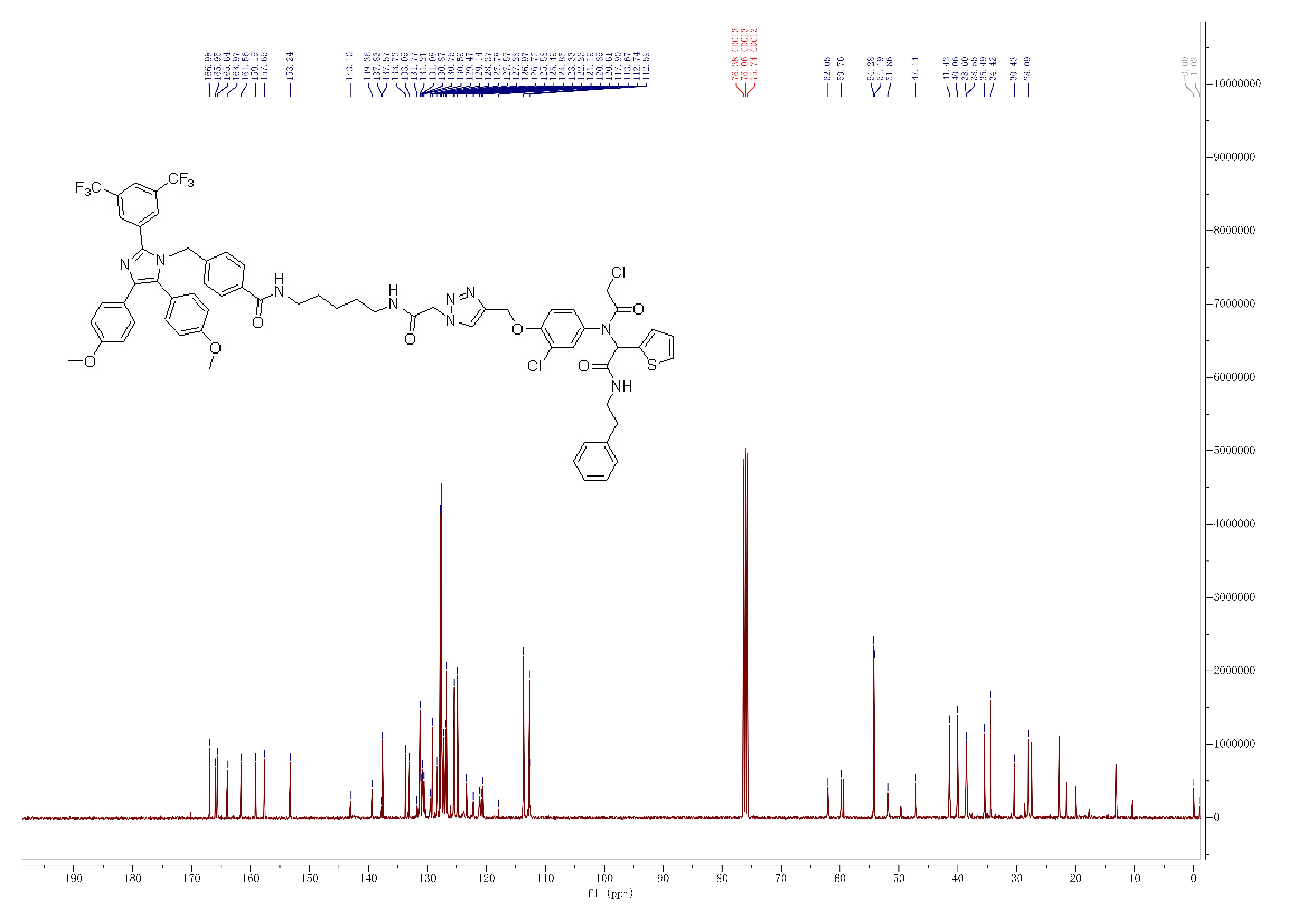


GDAz-10


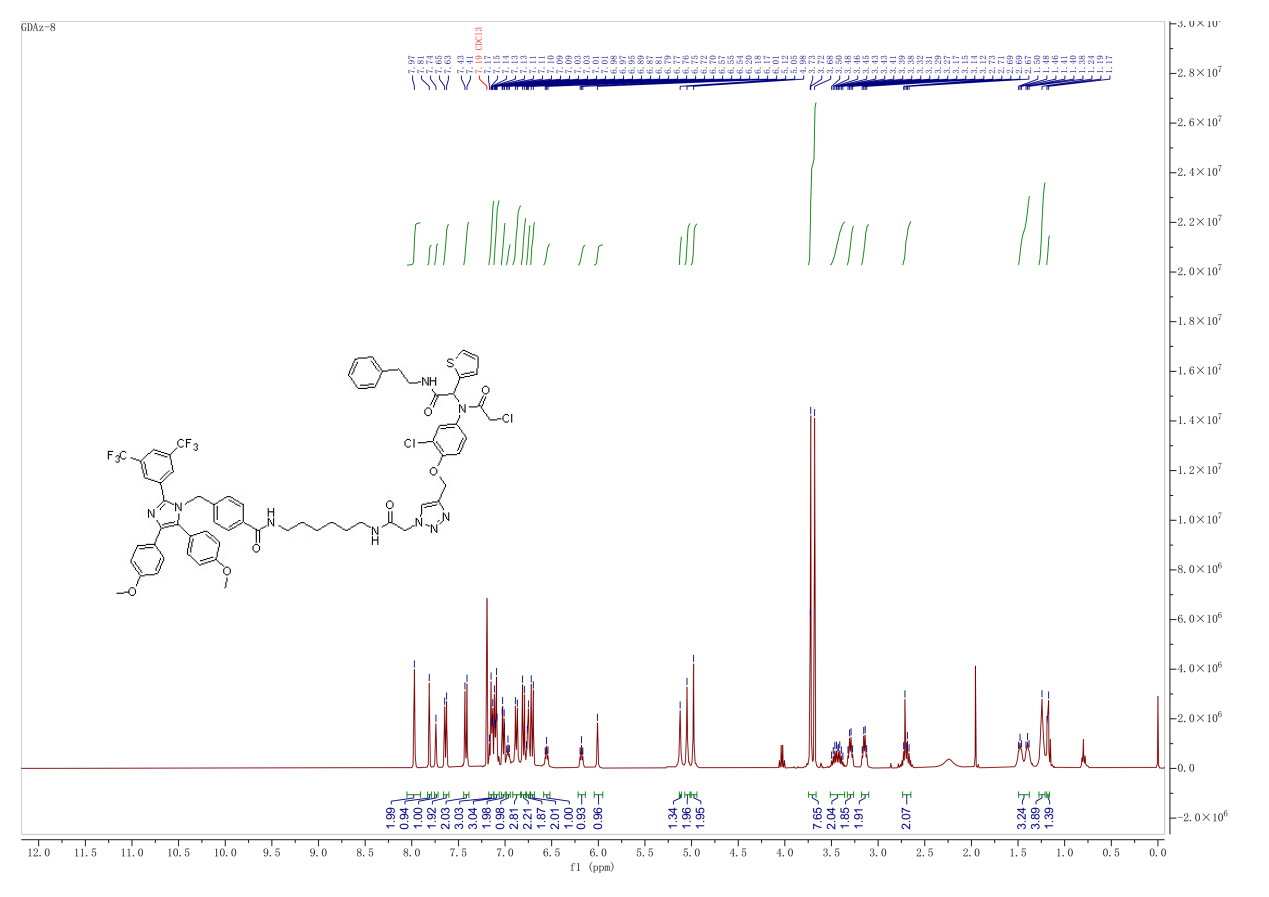


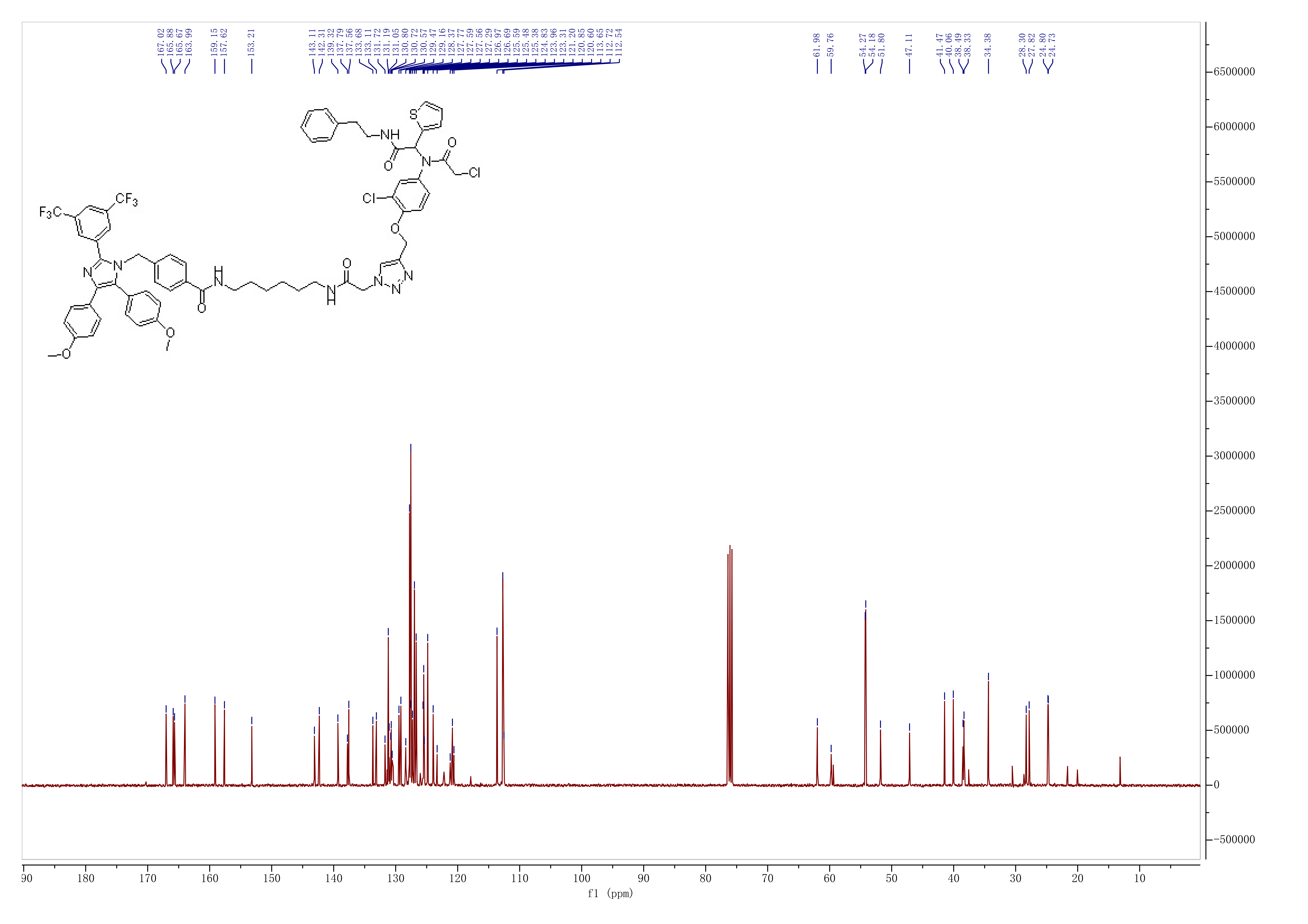


GDAz-11


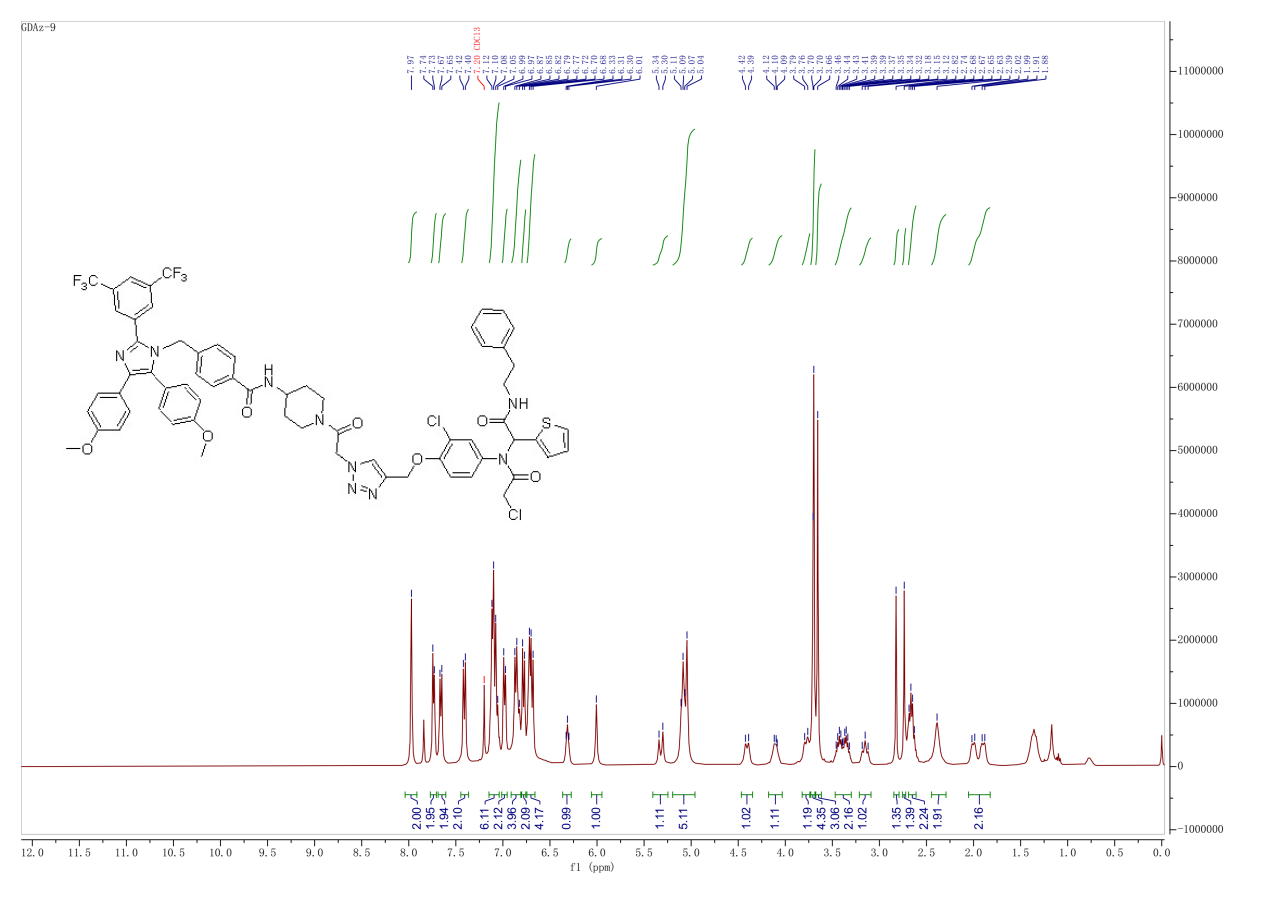


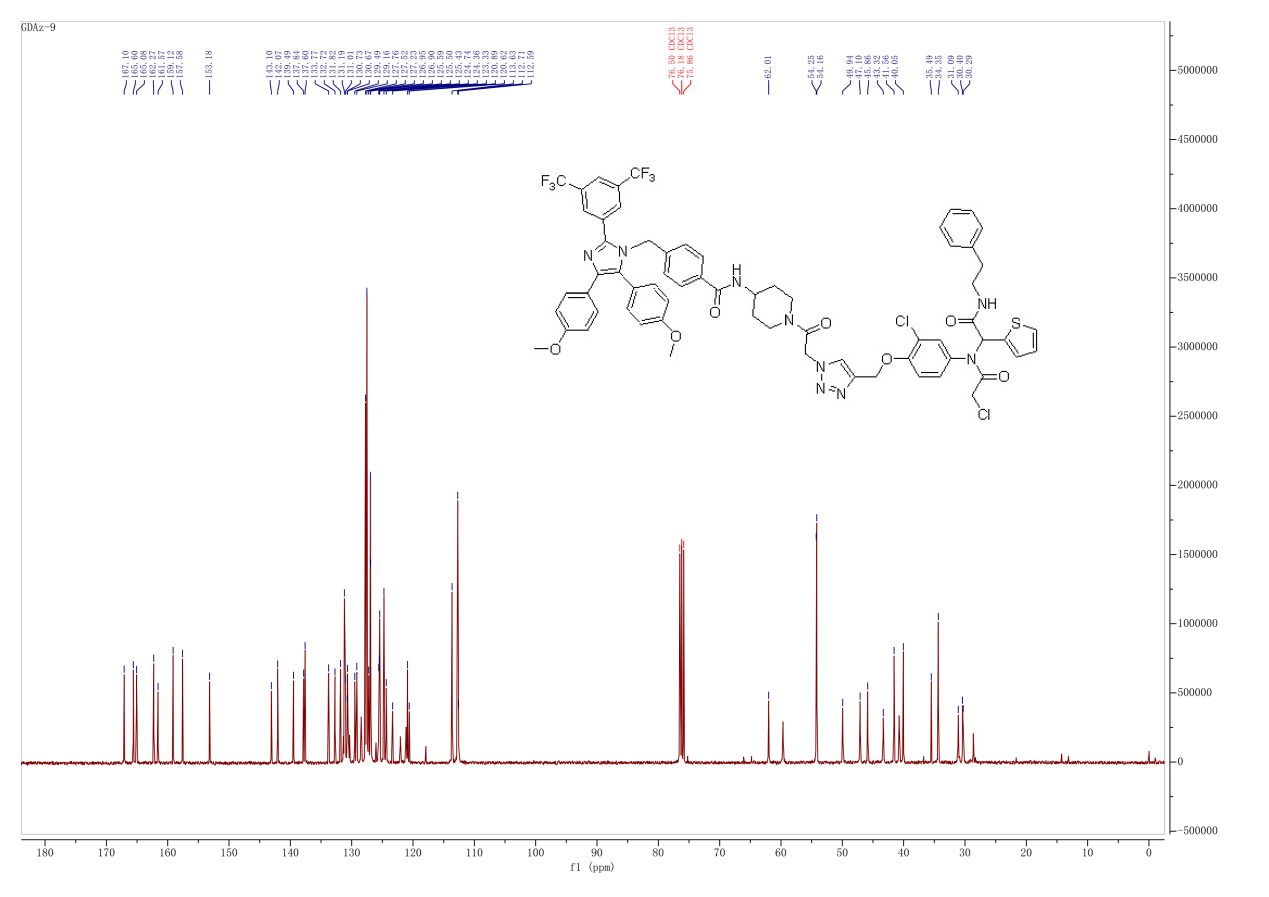


GDAz-12


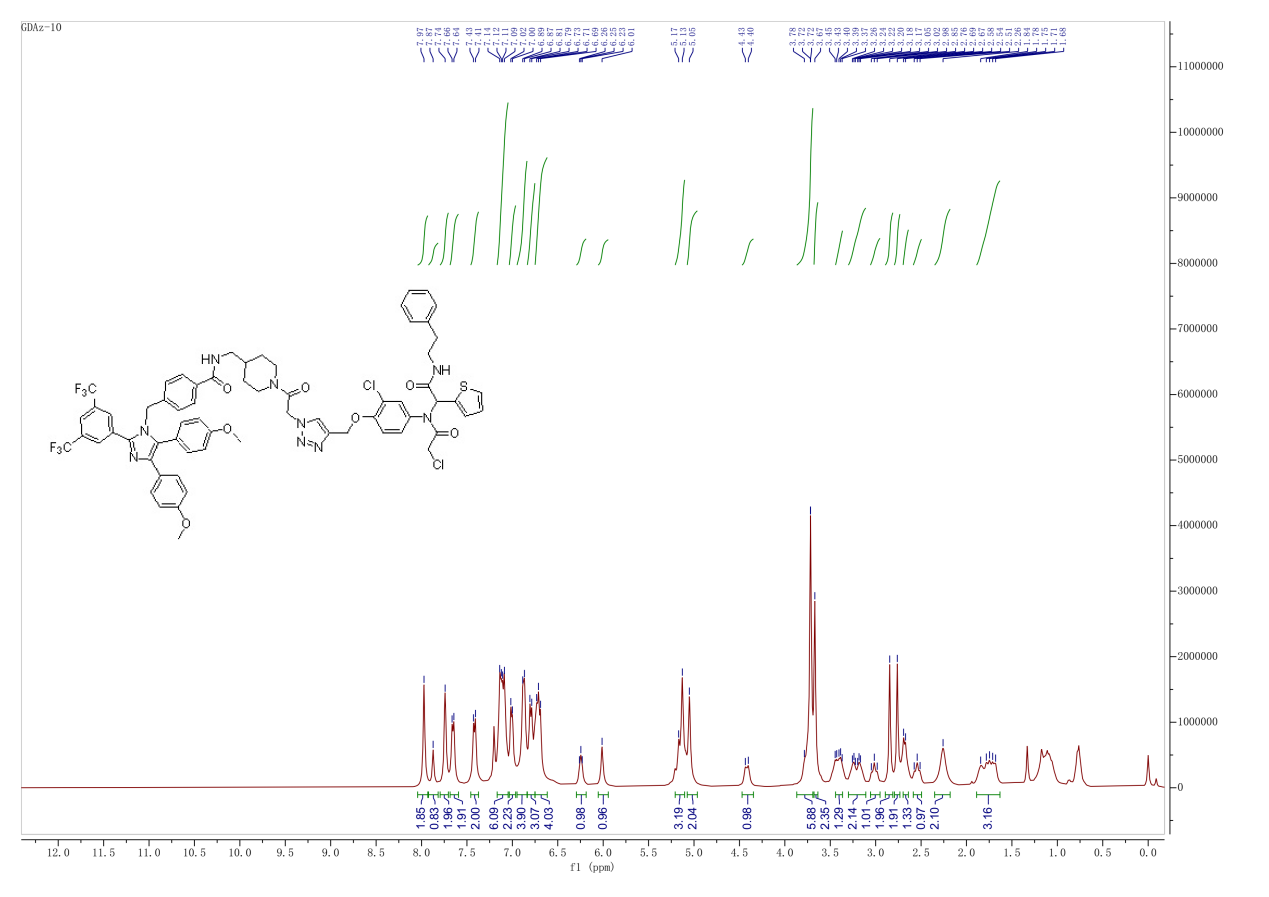


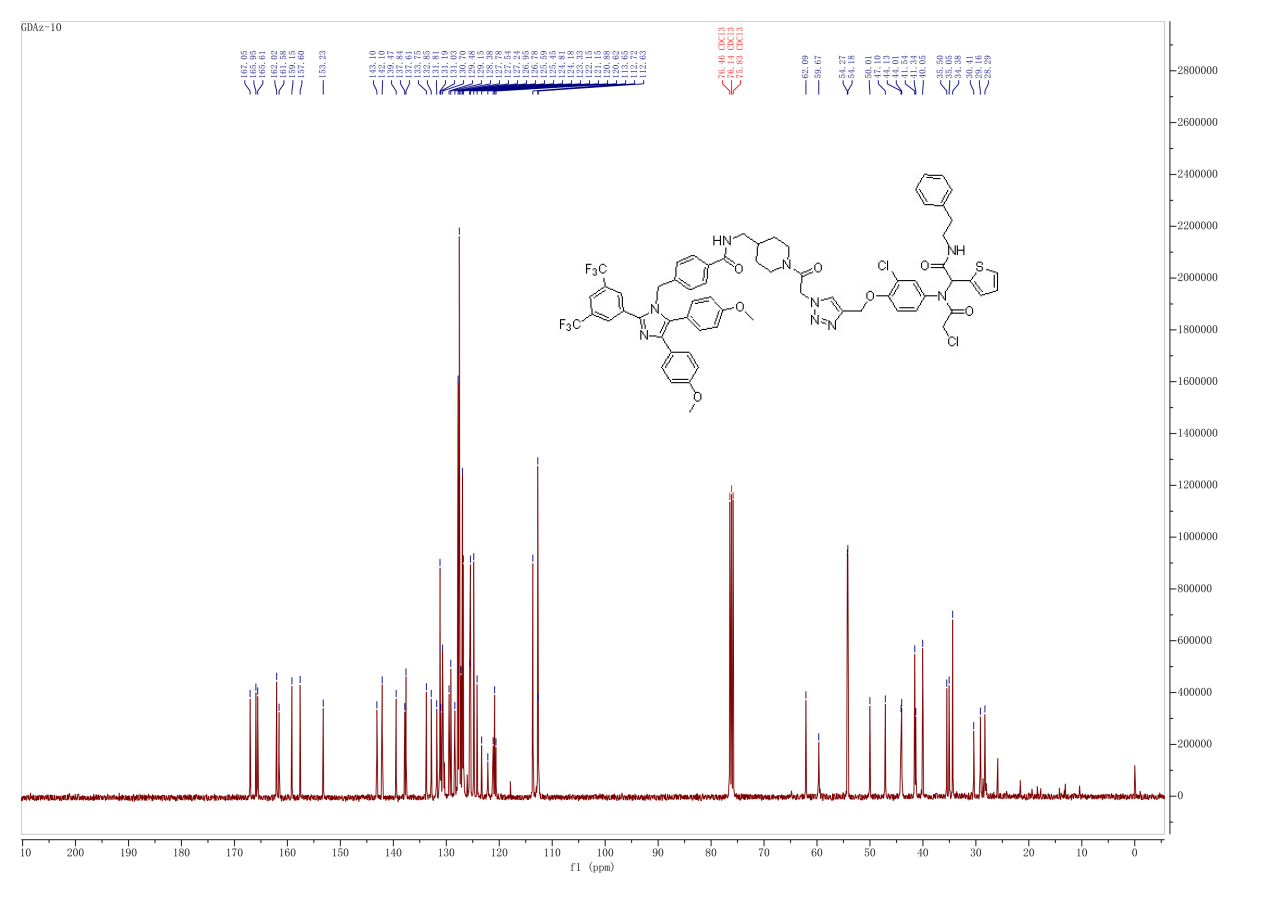


GDAz-13


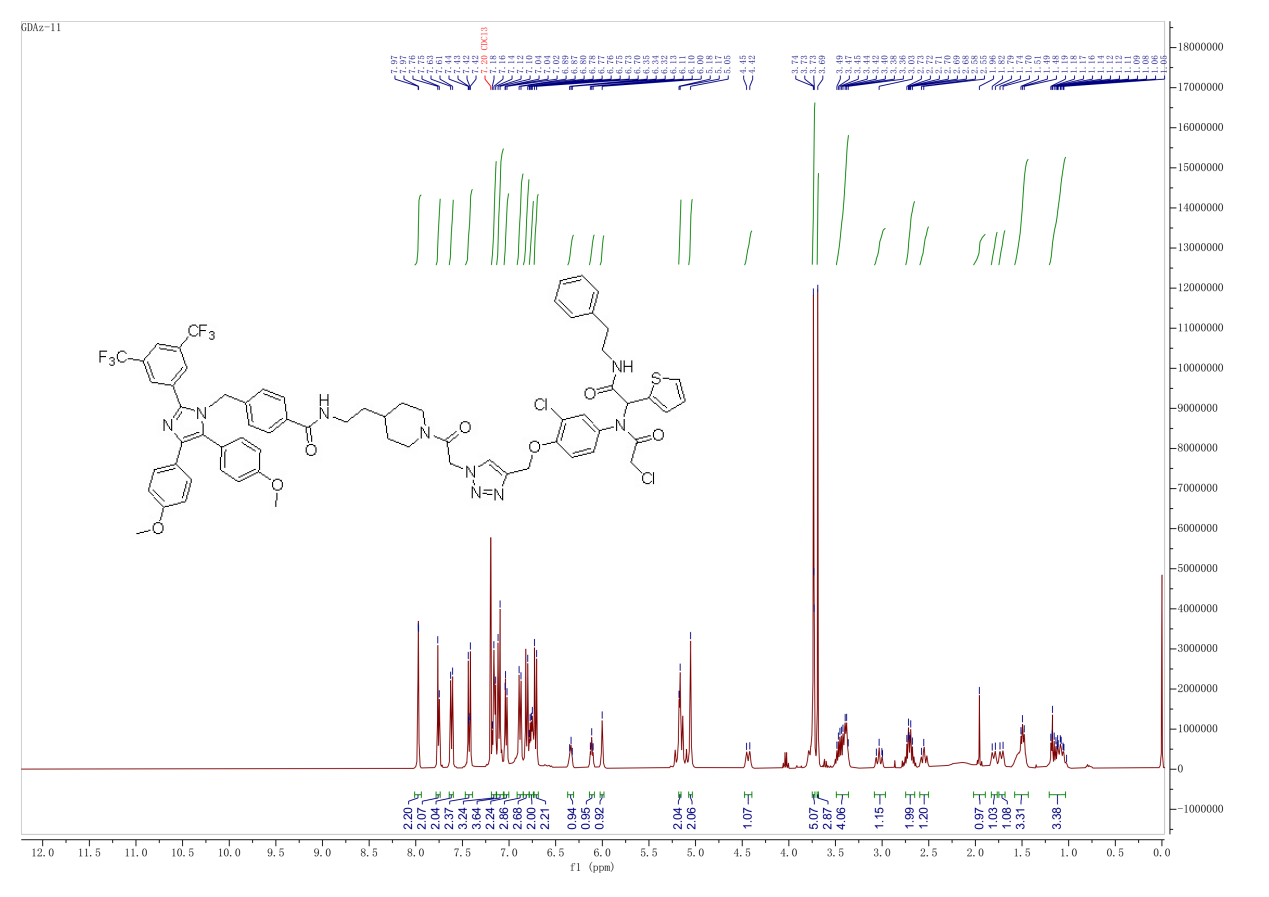


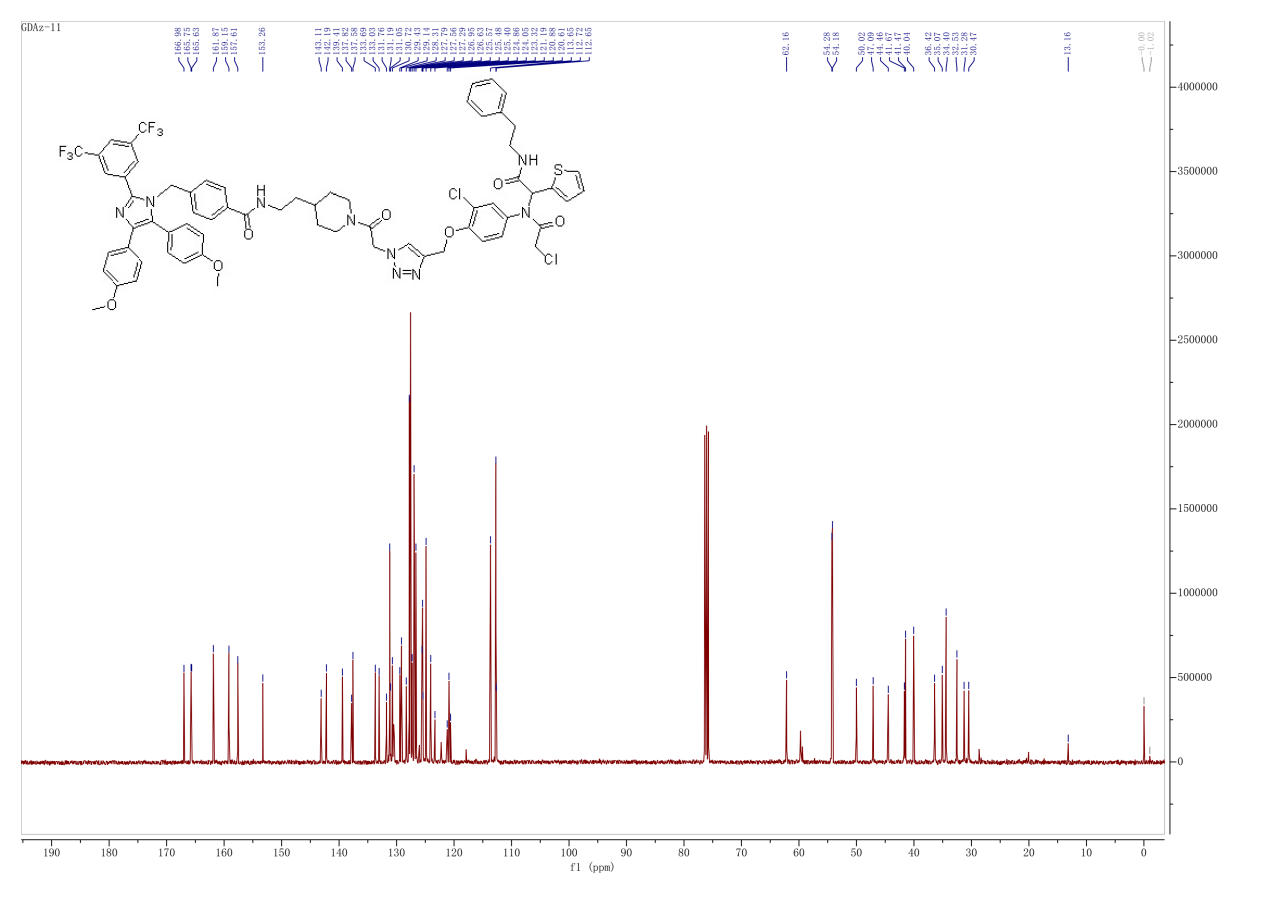


GDAz-14


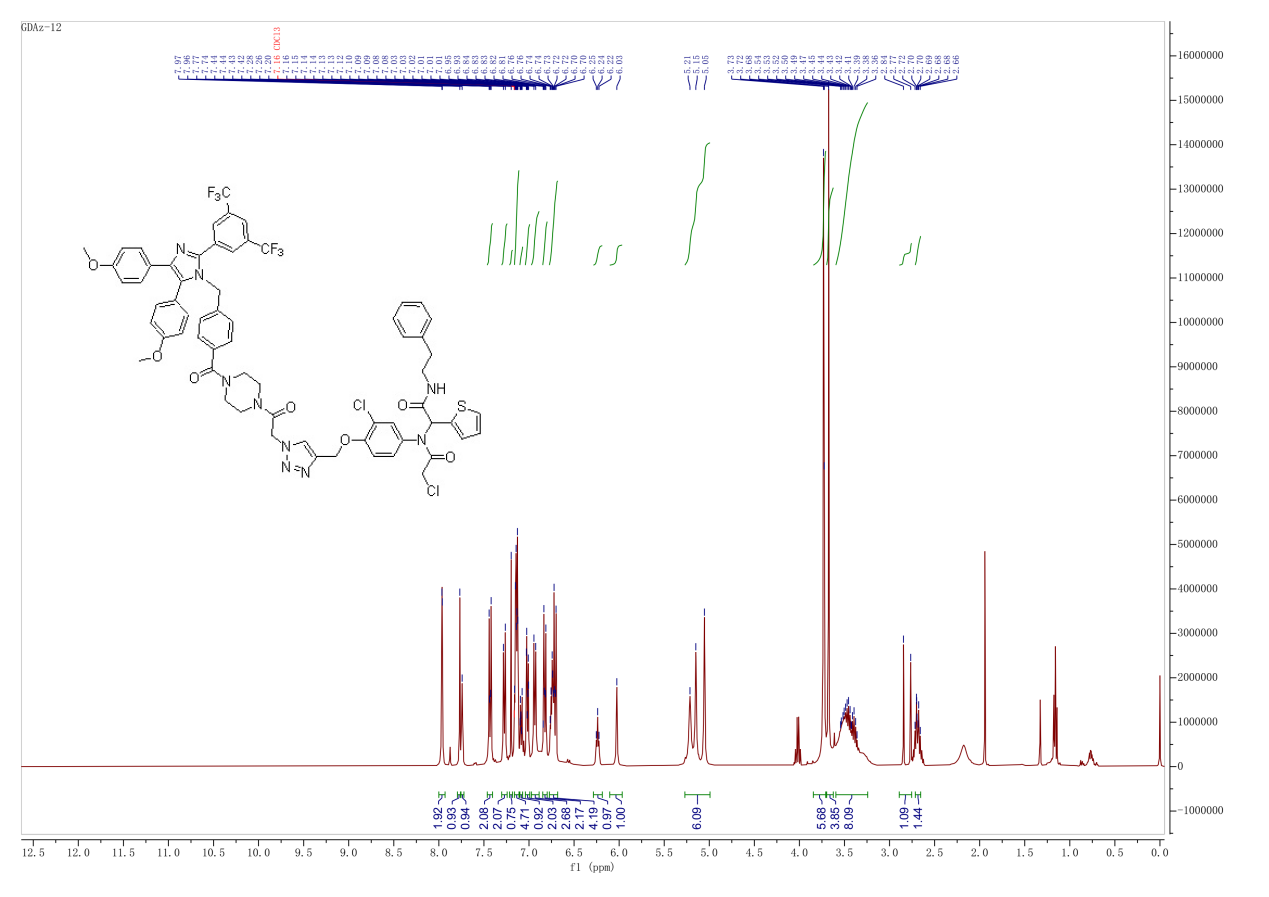


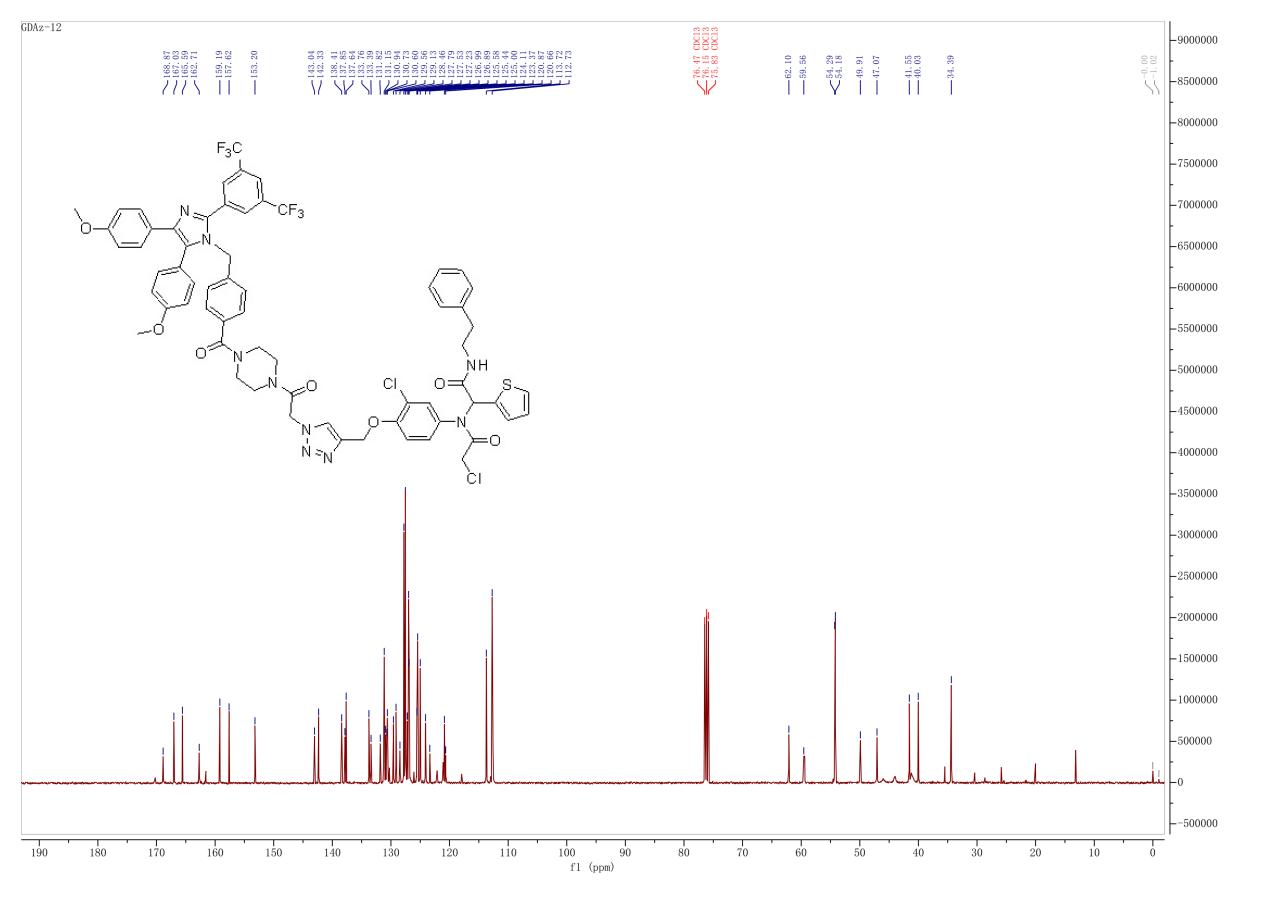


GDAz-Neg1


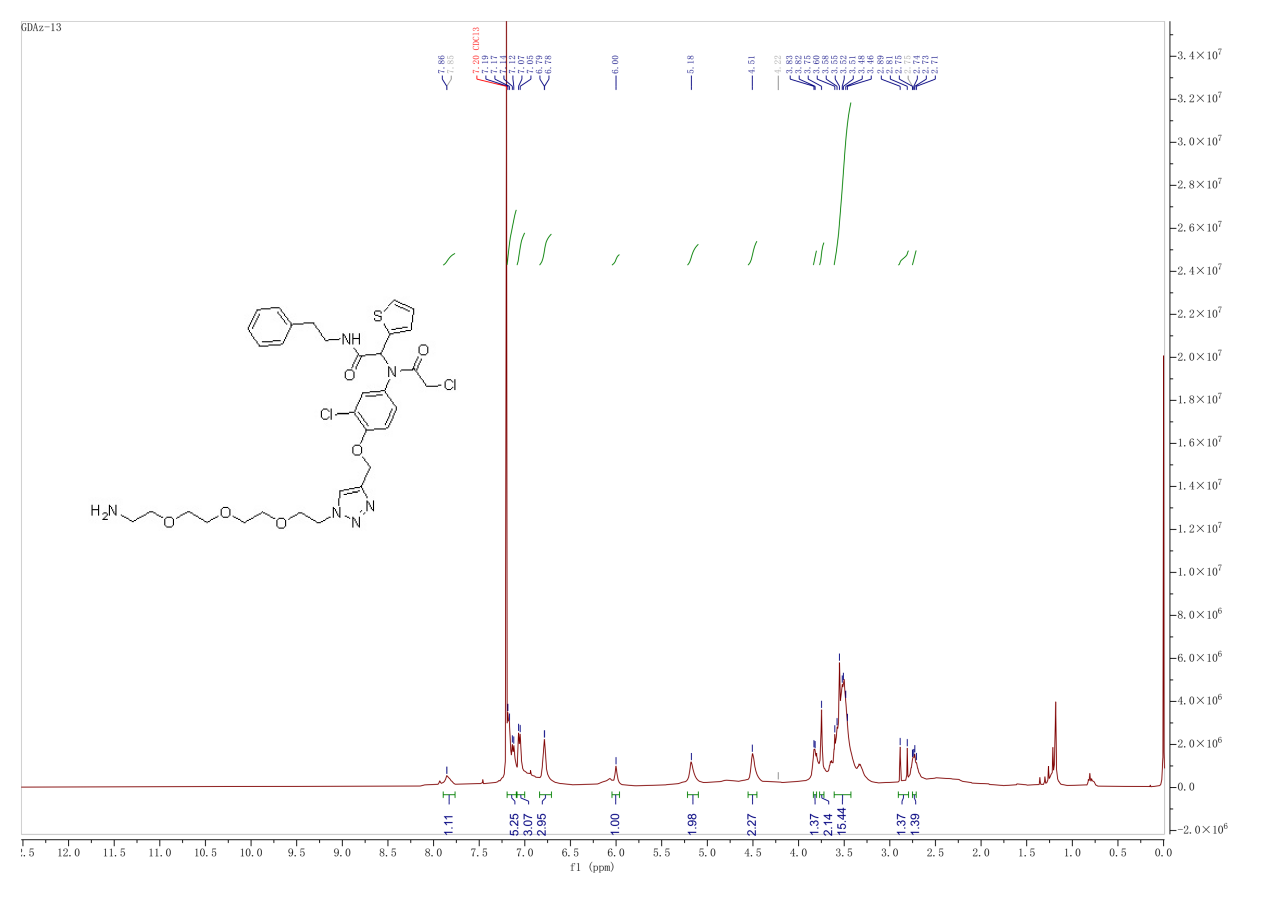


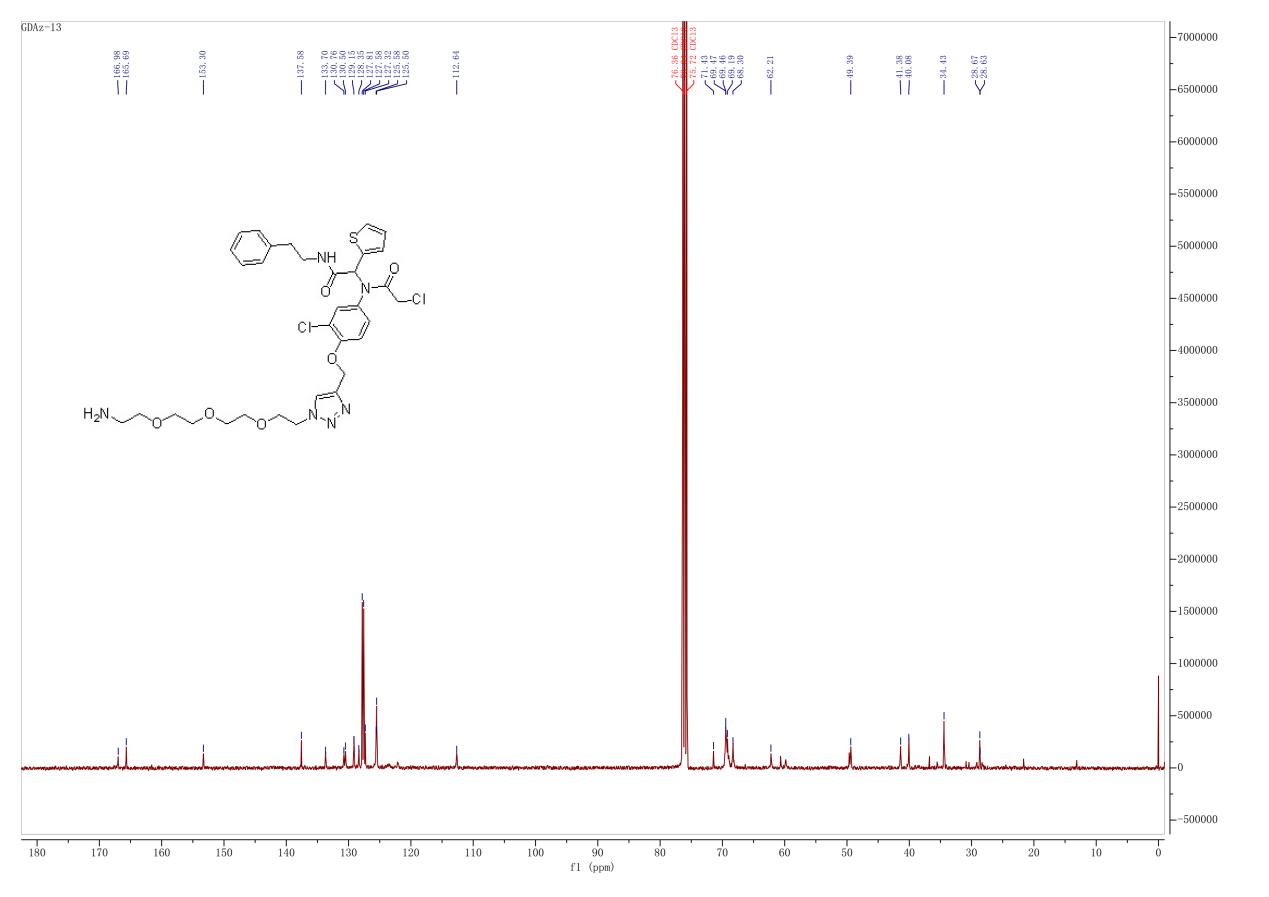


GDAz- Neg2


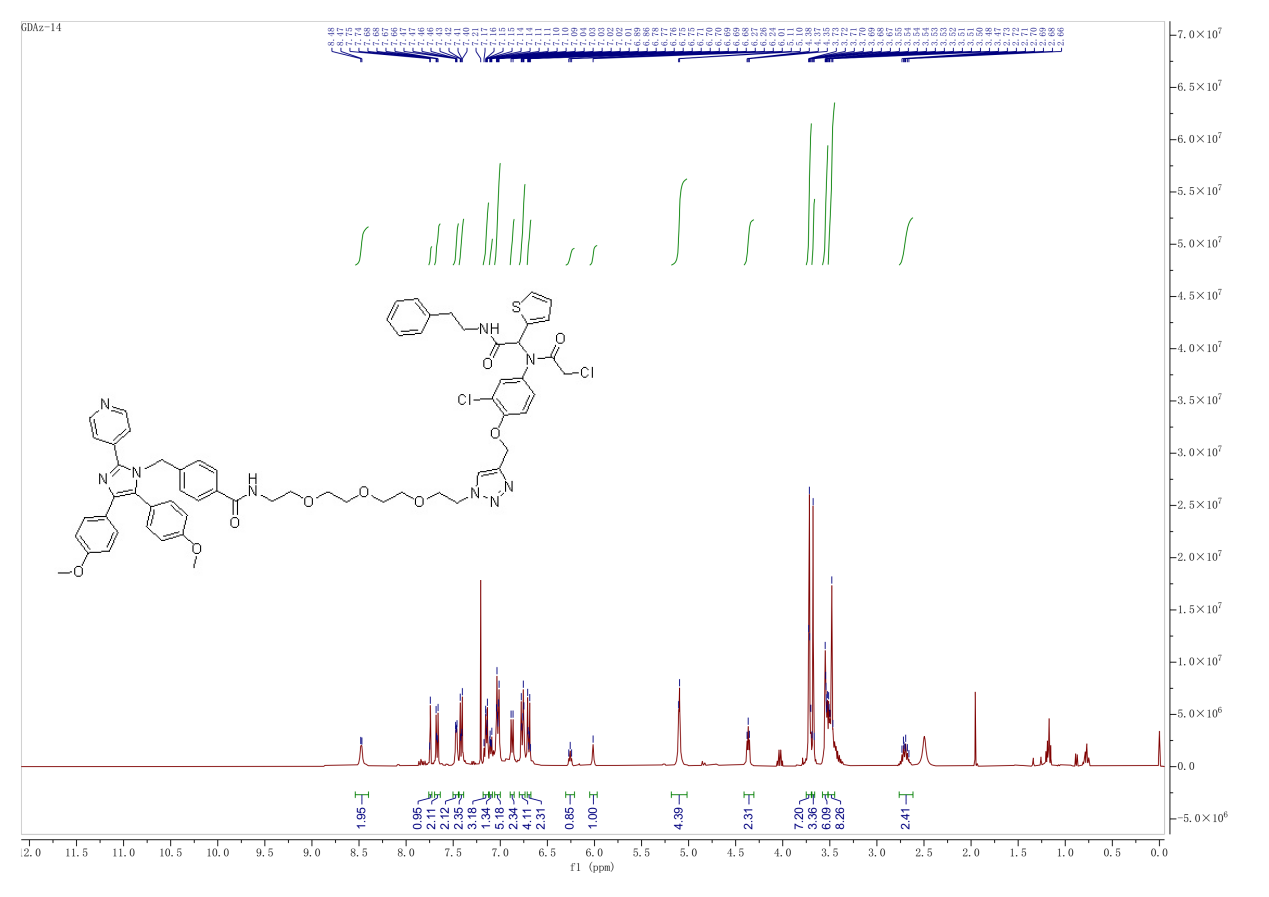


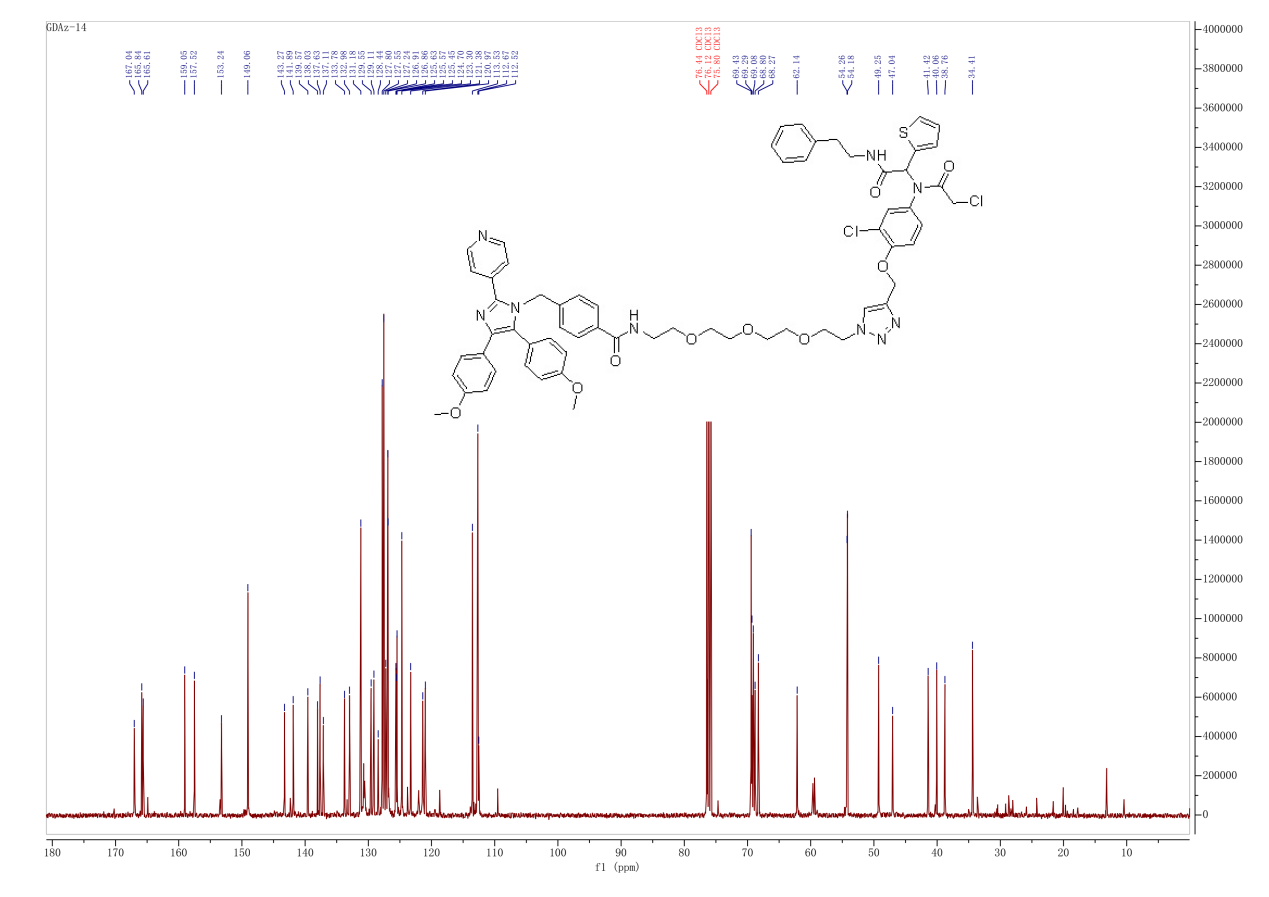


GDAz-15


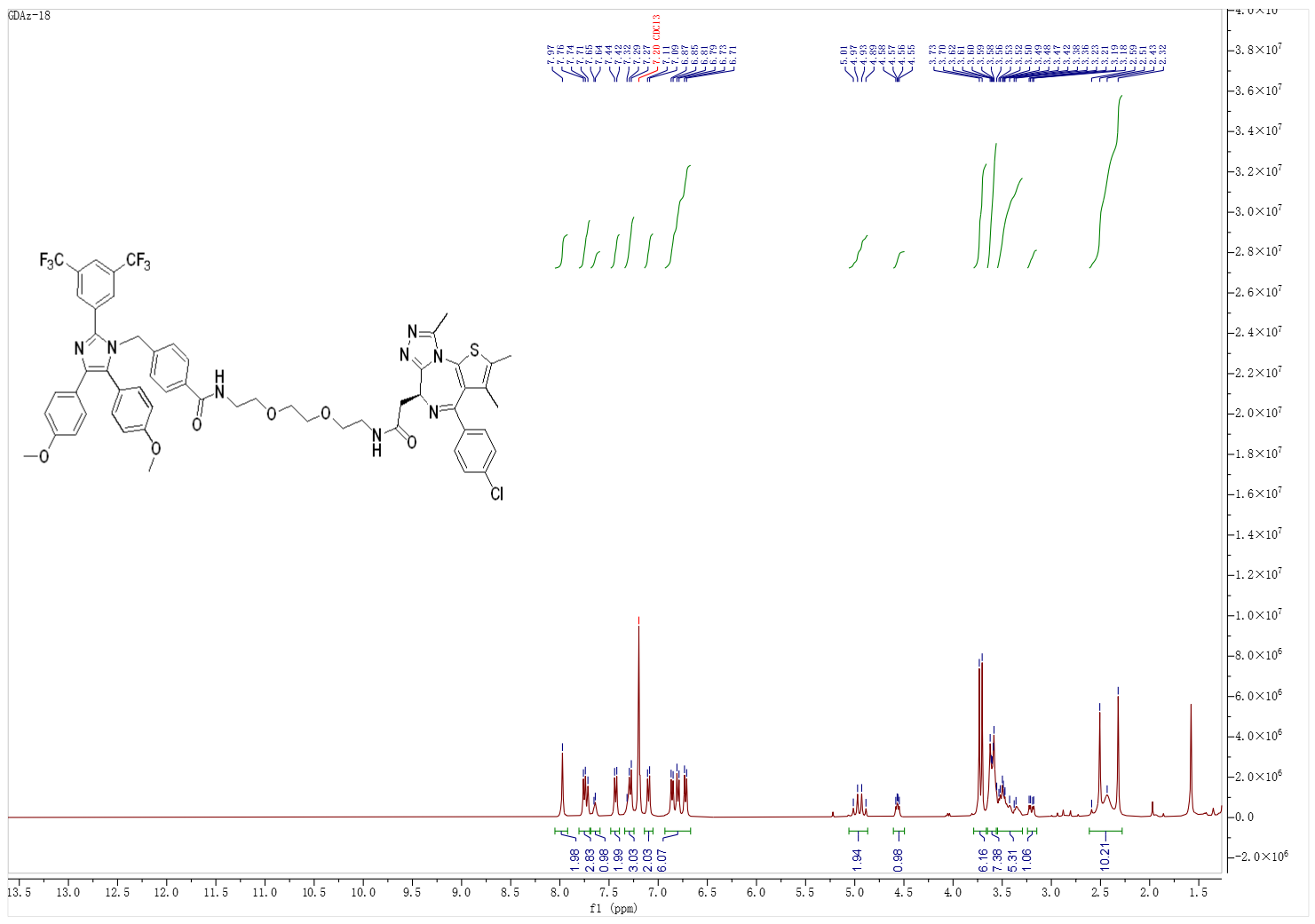


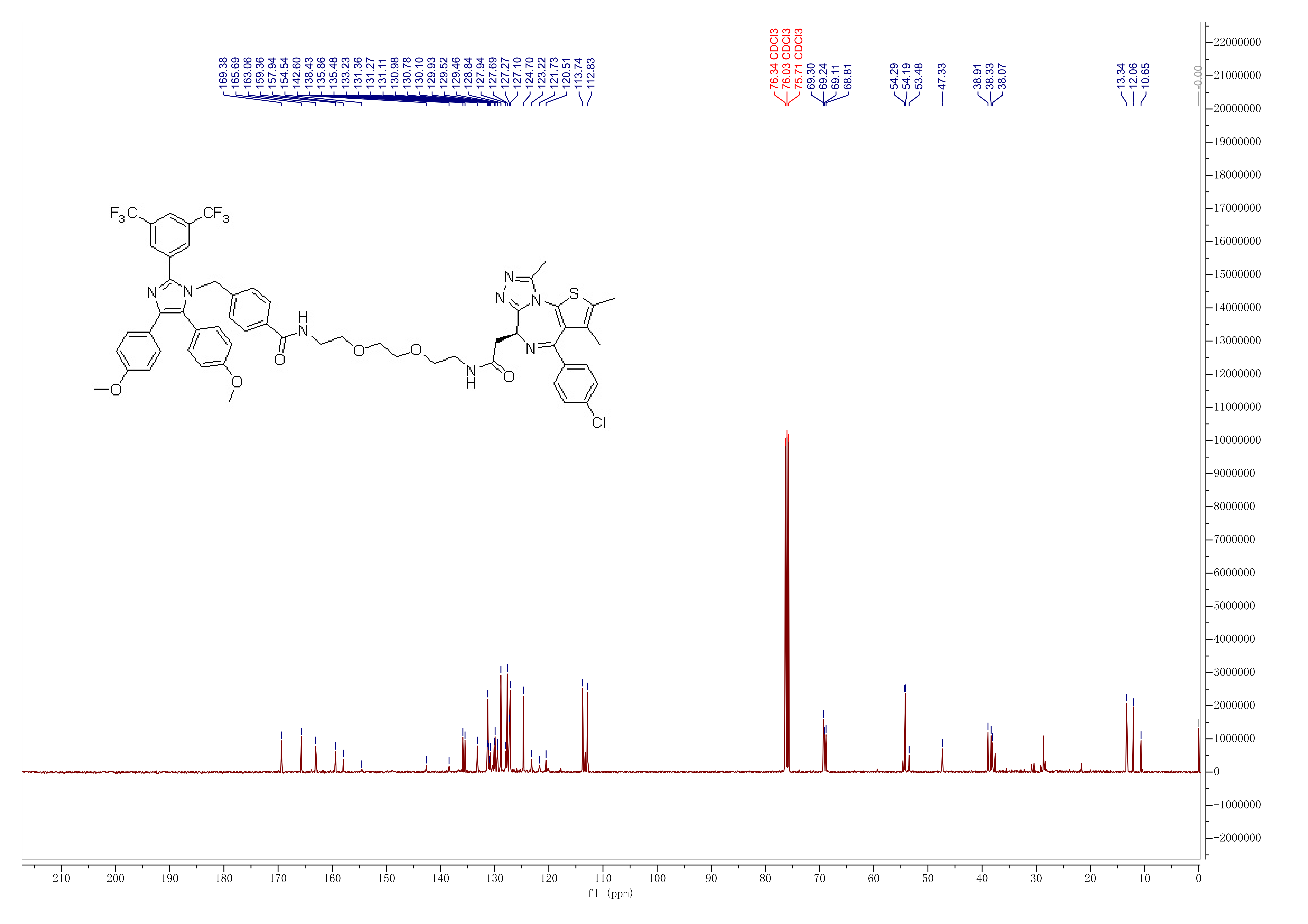


GDAz-16


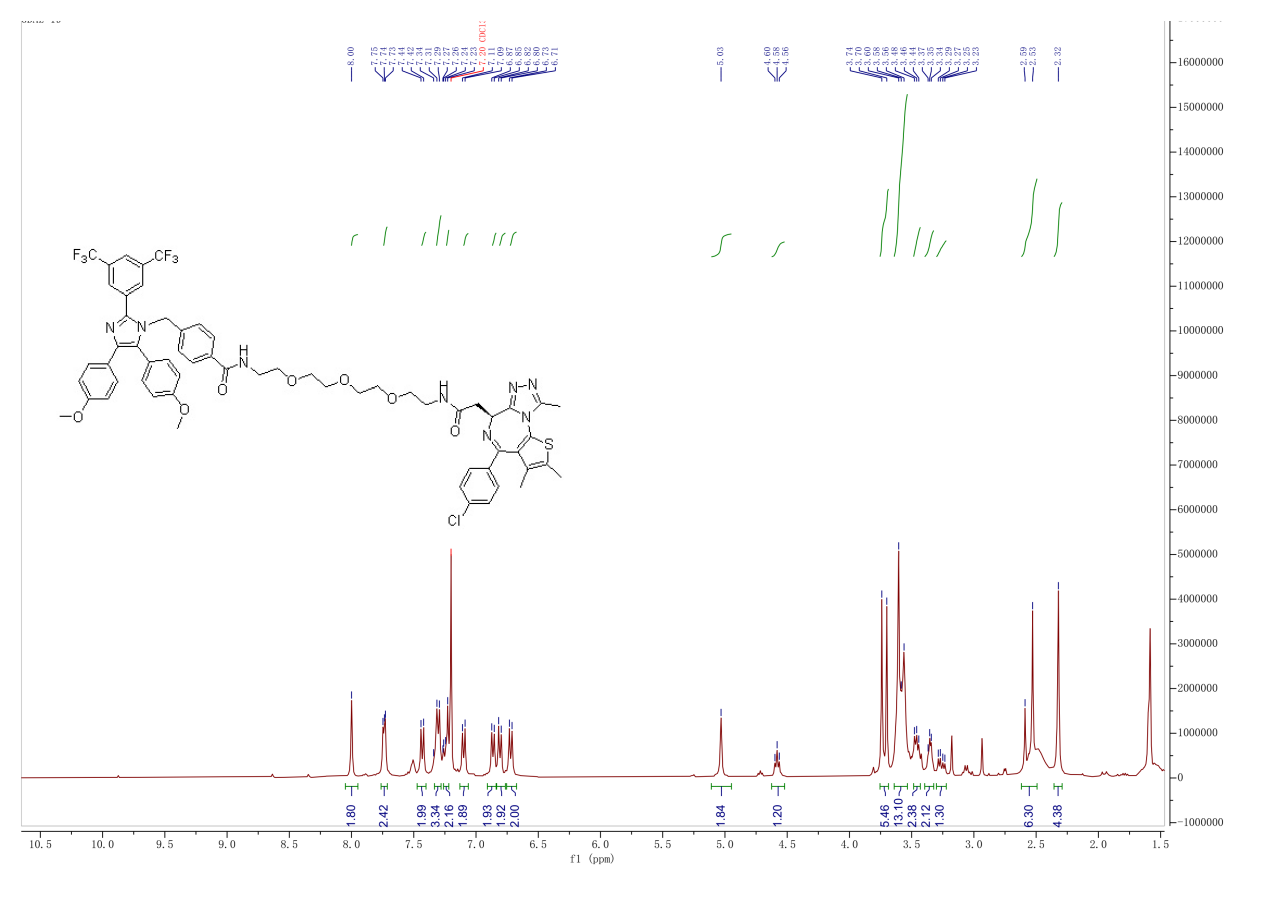


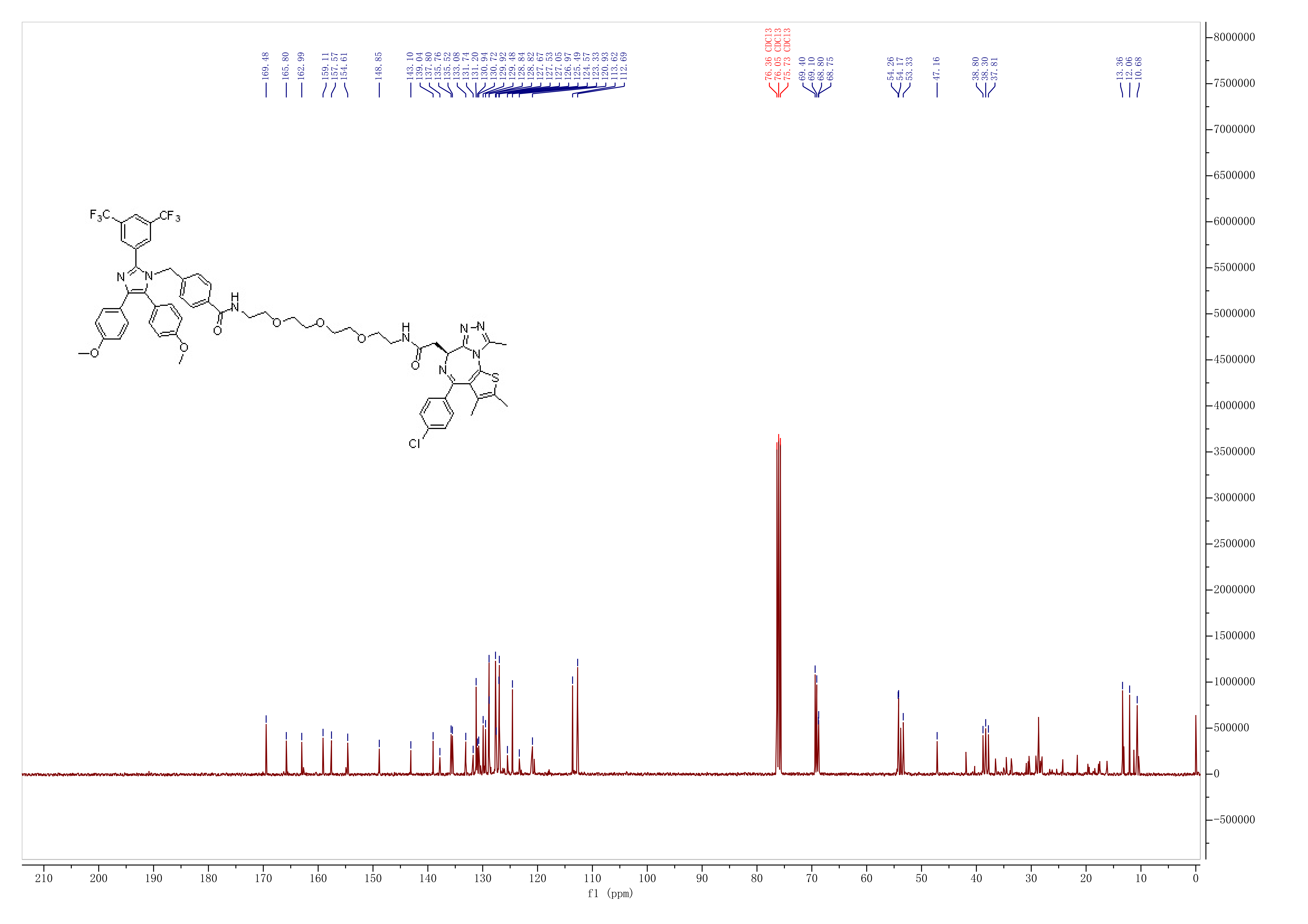


GDAz-17


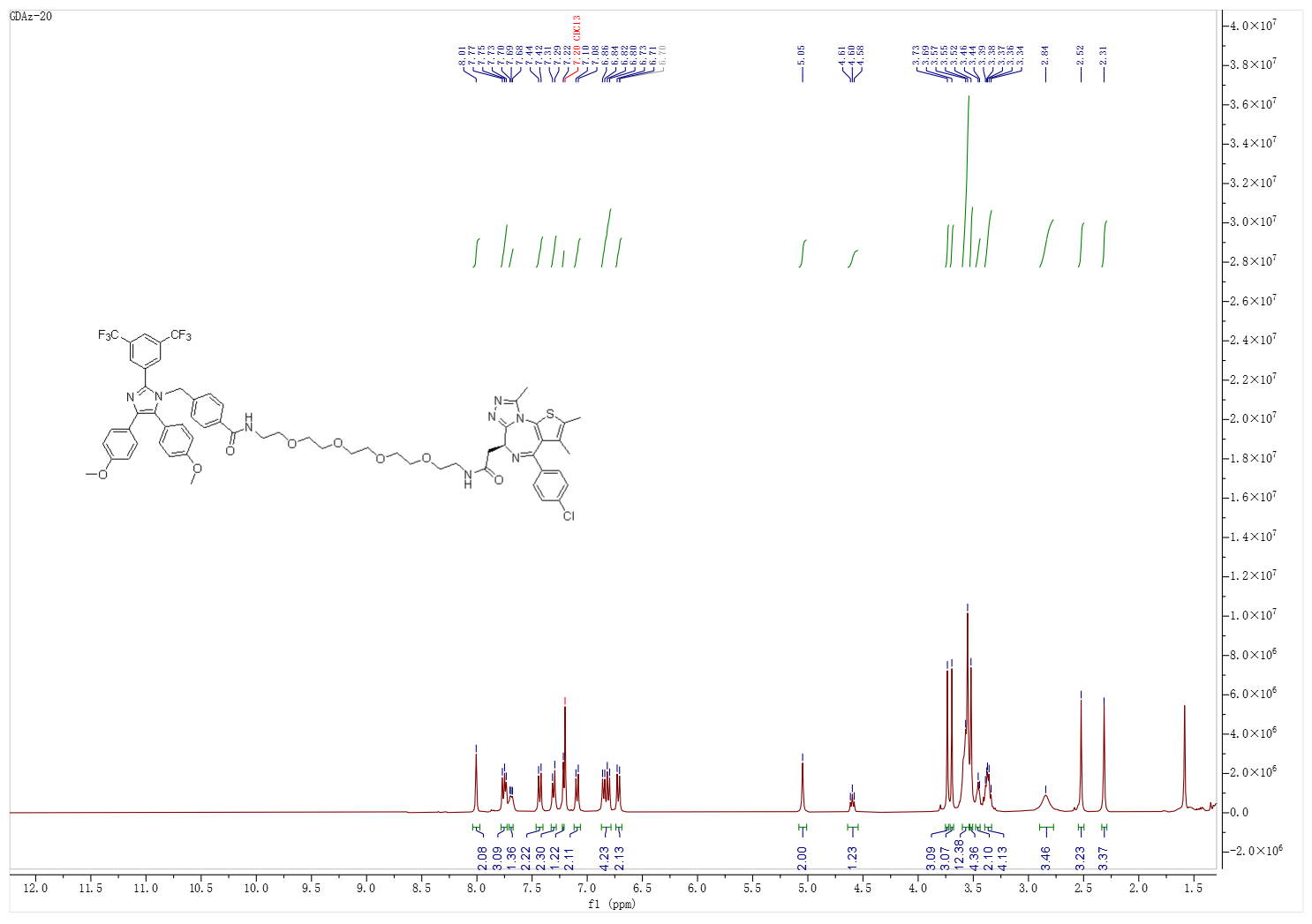


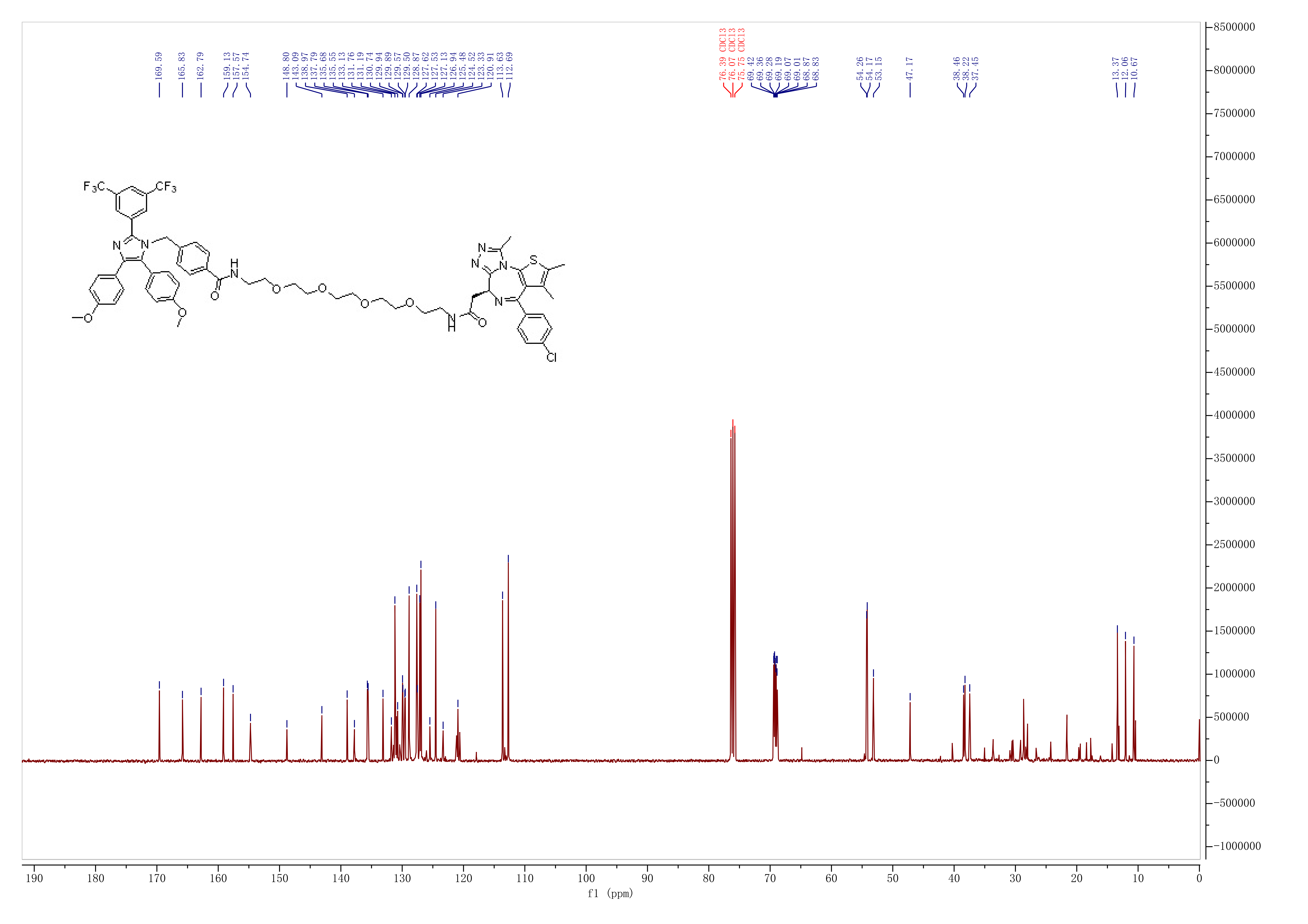


**HRMS for target compounds**

GDAz-1


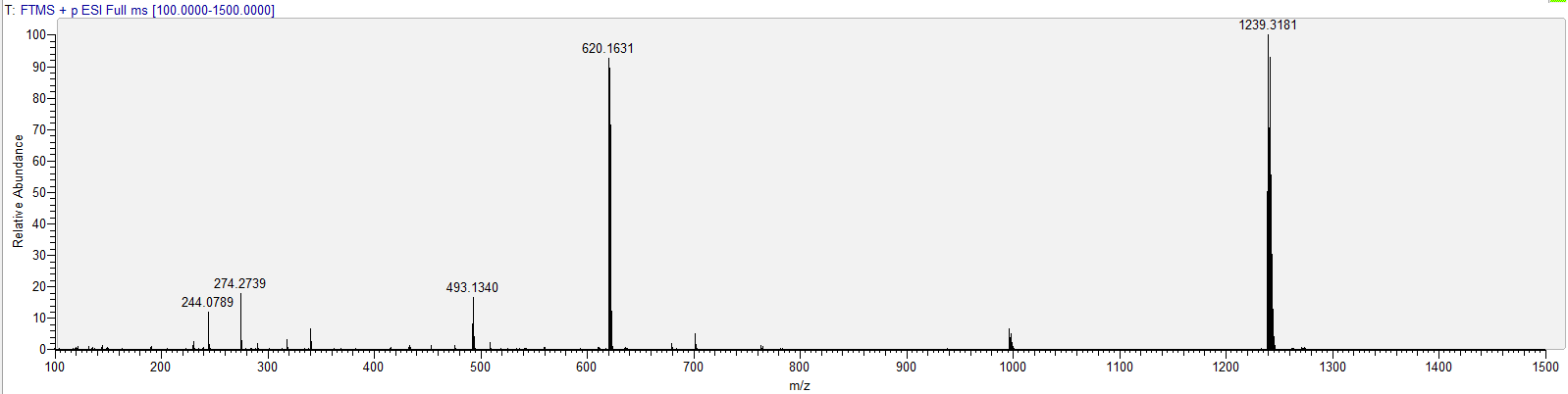


GDAz-2


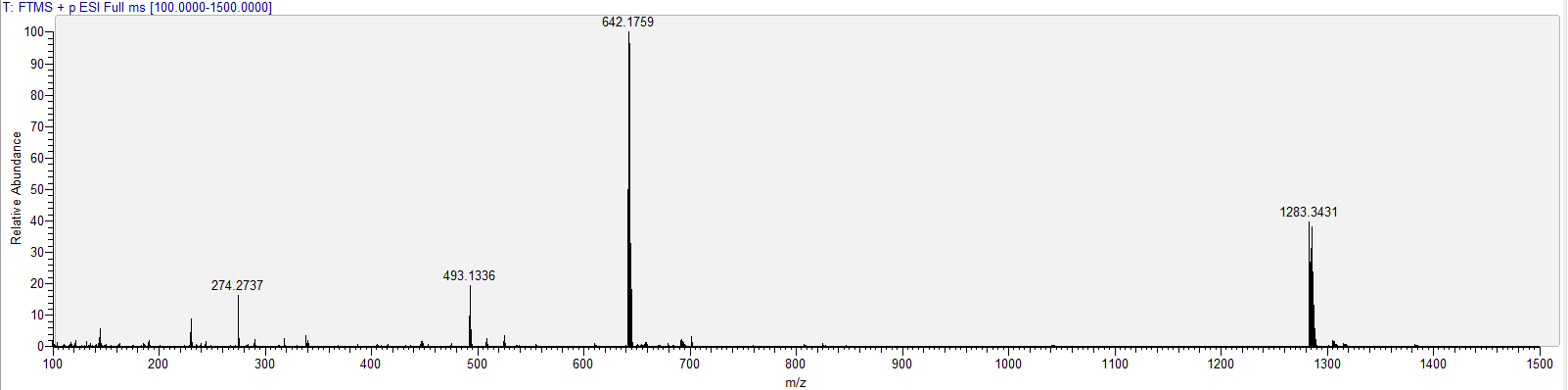


GDAz-3


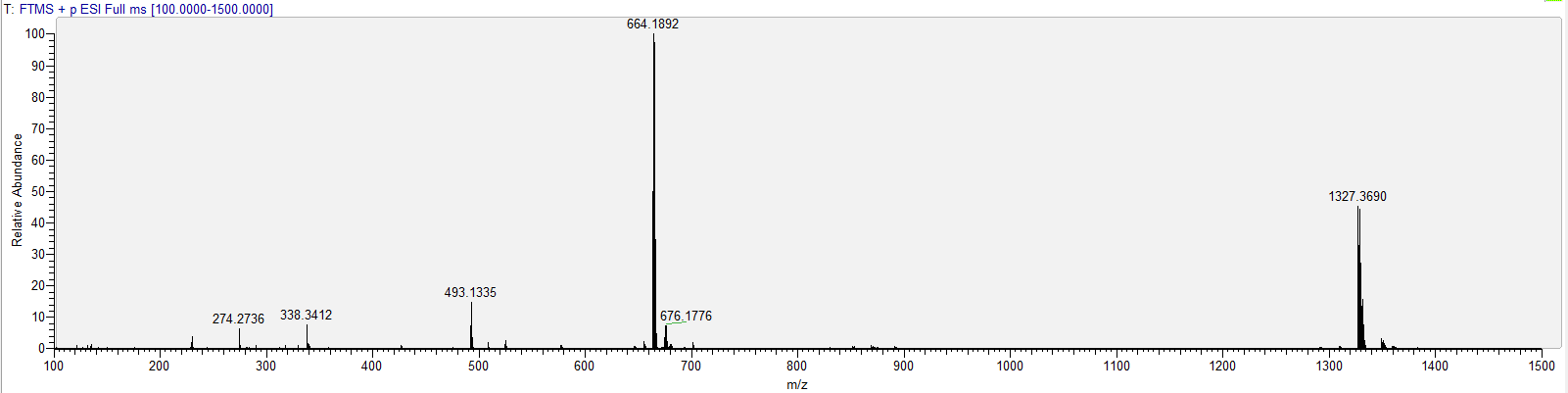


GDAz-4


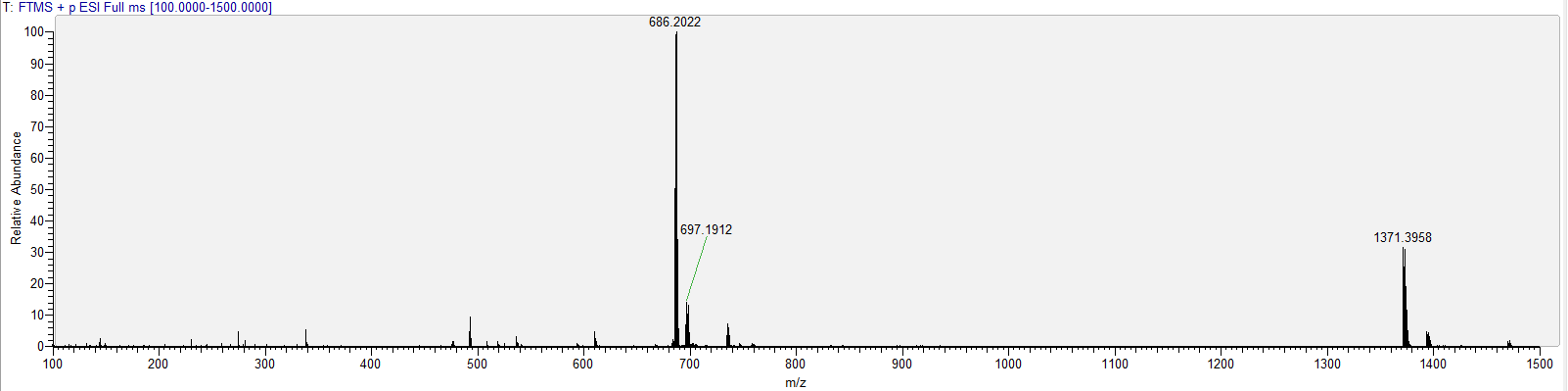


GDAz-5


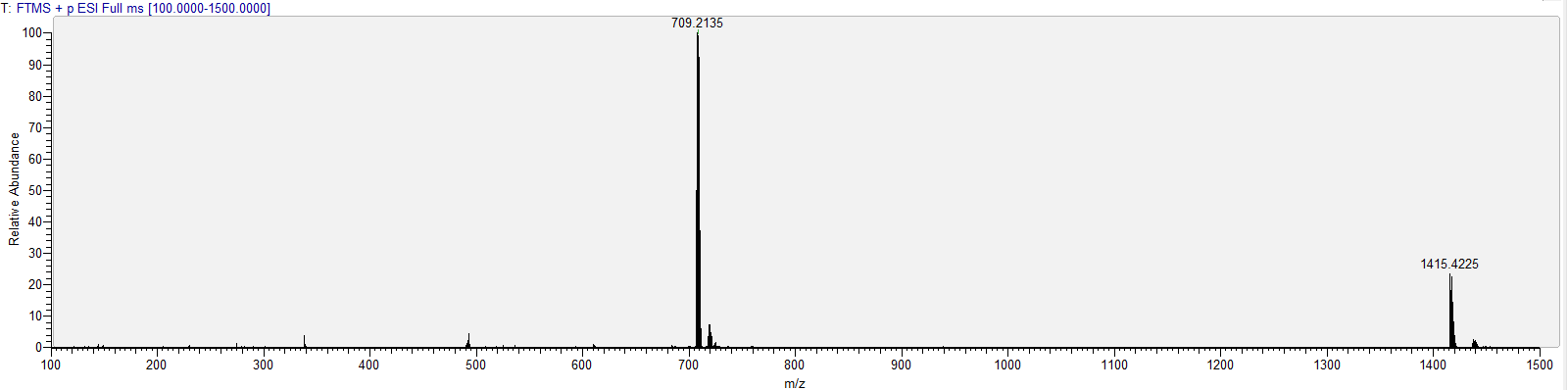


GDAz-6


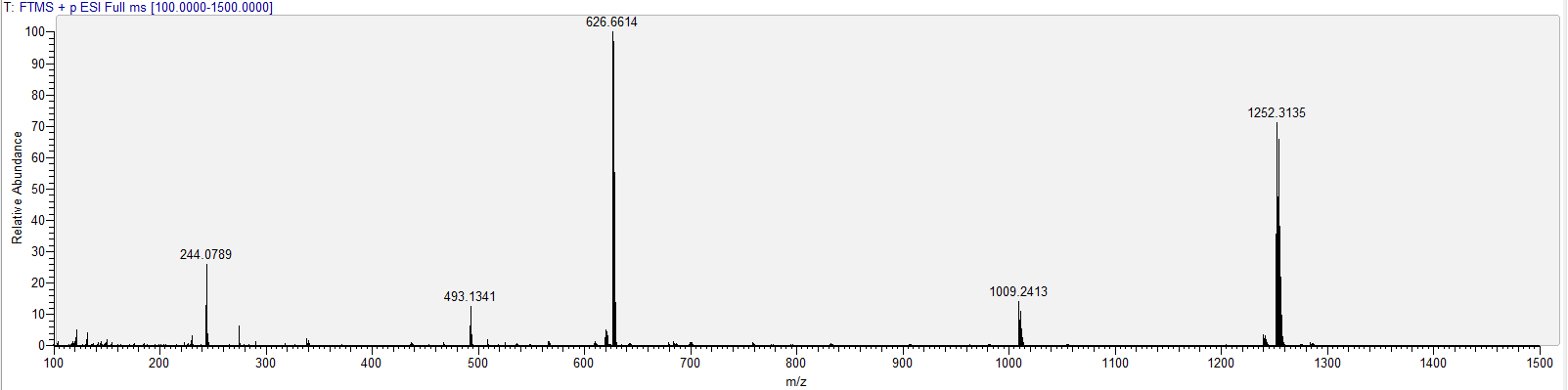


GDAz-7


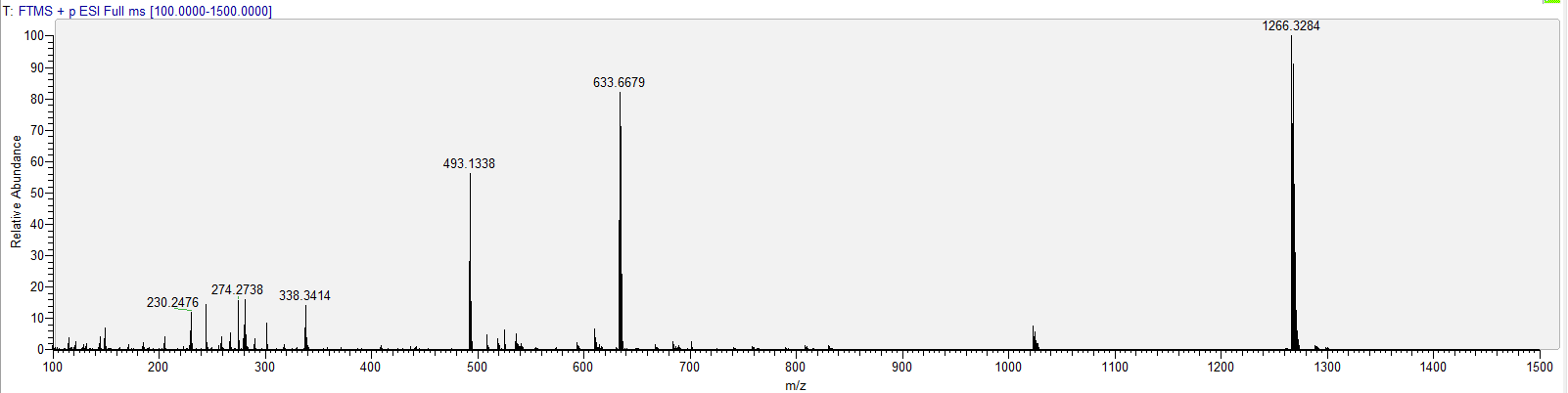


GDAz-8


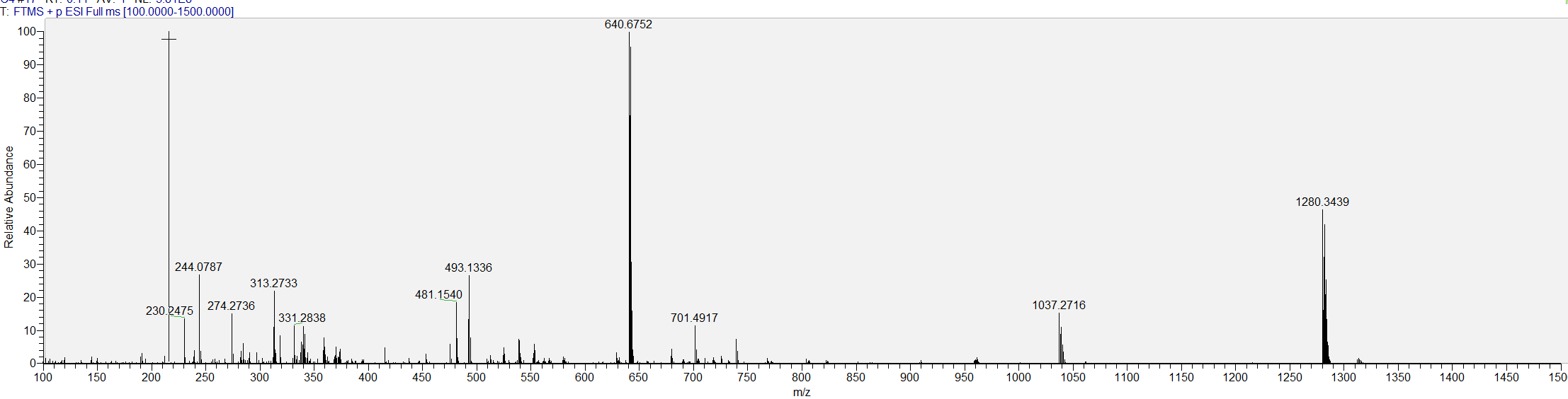


GDAz-9


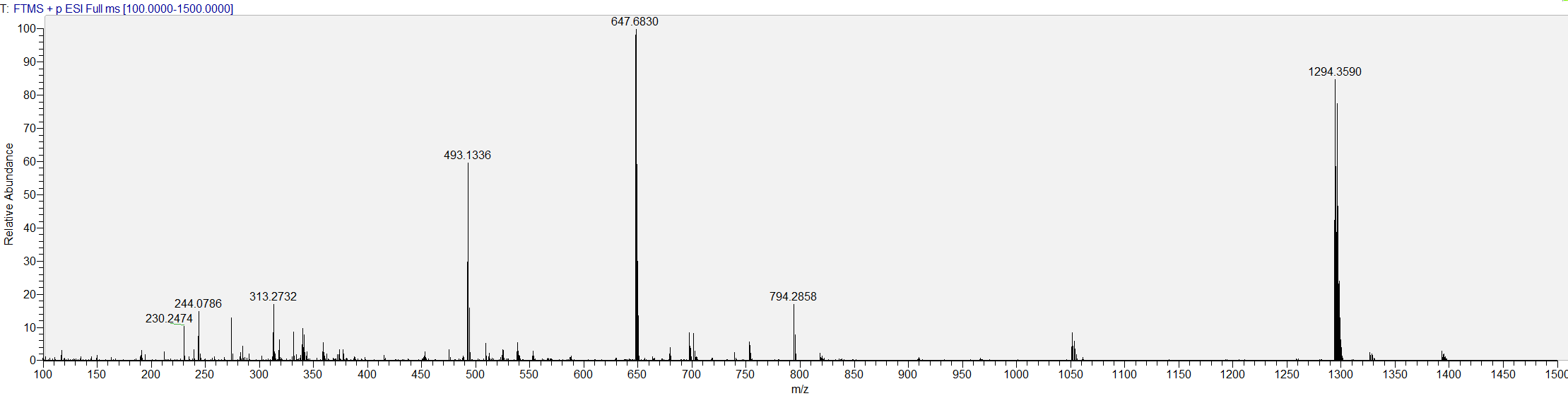


GDAz-10


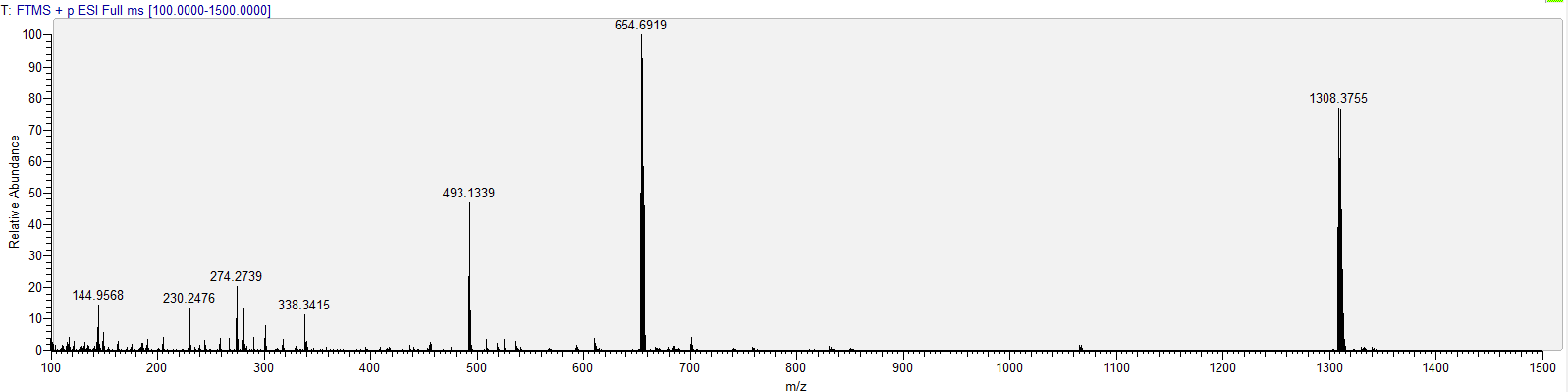


GDAz-11


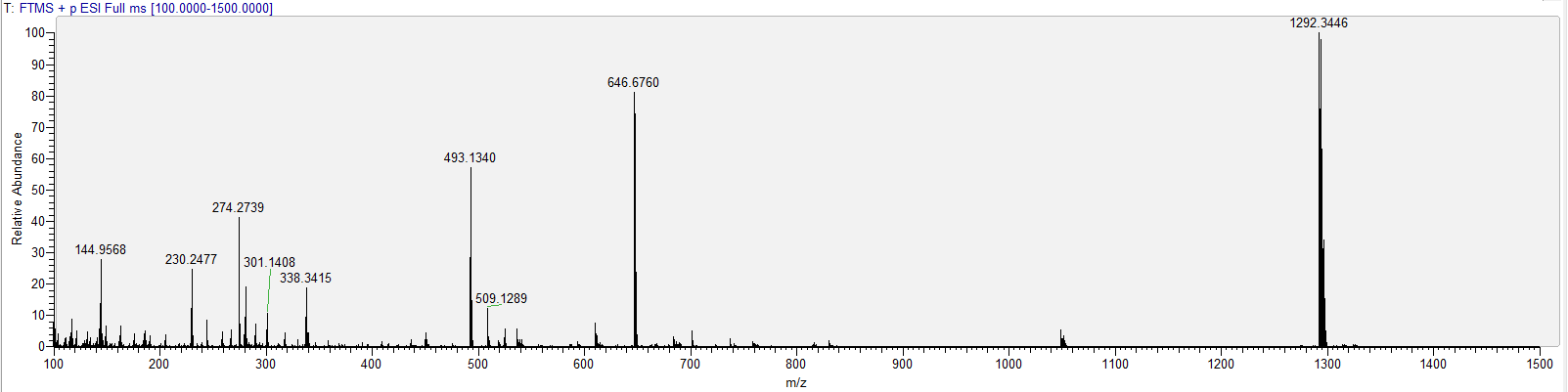


GDAz-12


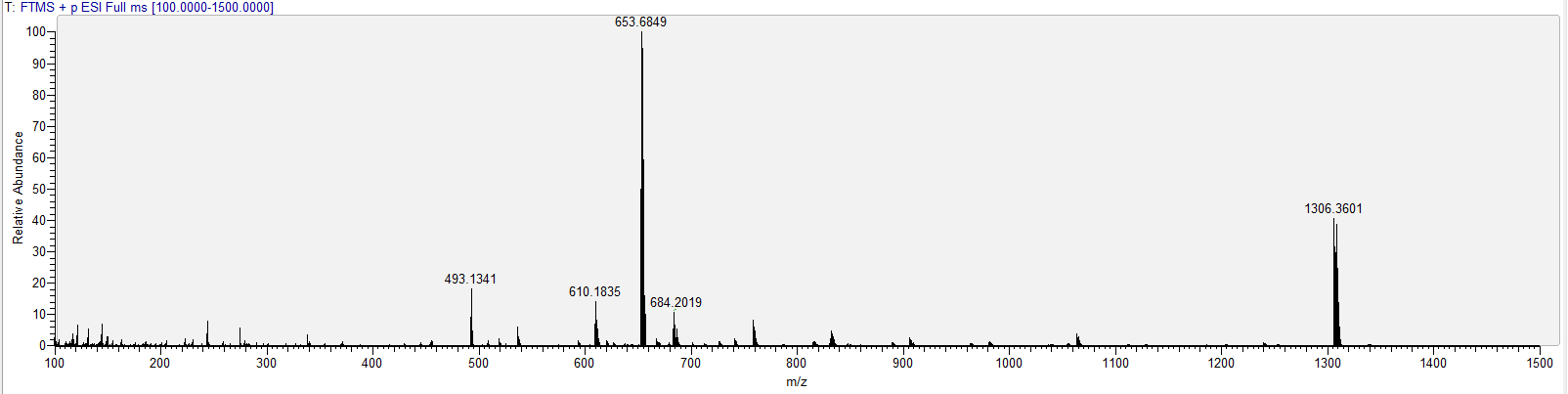


GDAz-13


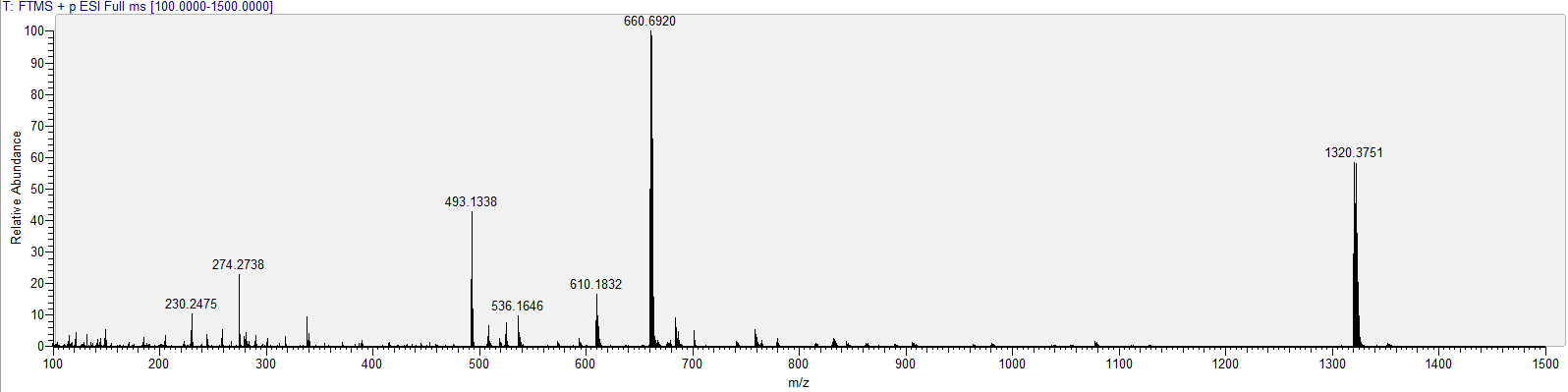


GDAz-14


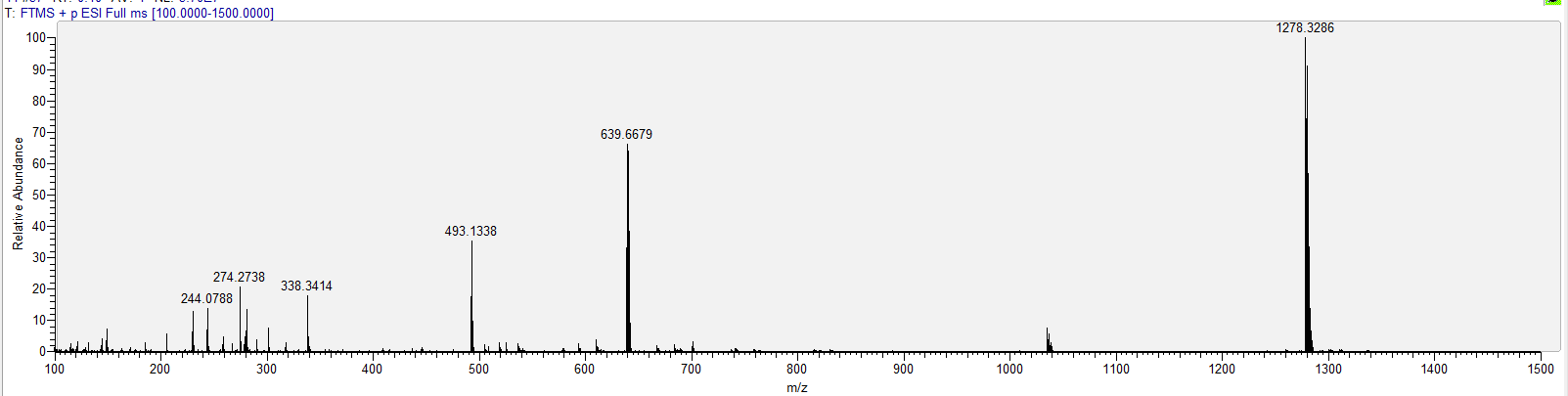


GDAz-Neg1


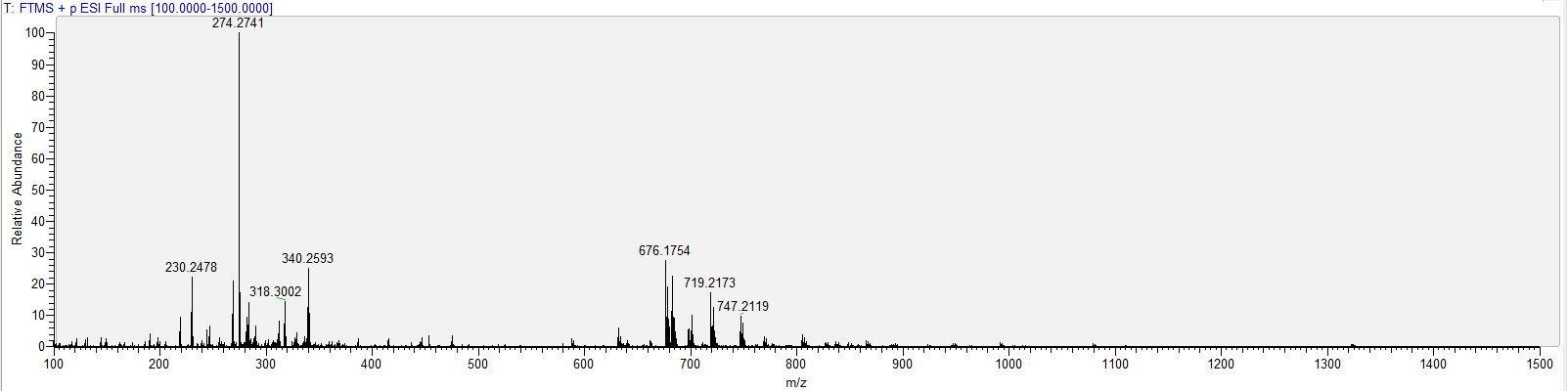


GDAz- Neg2


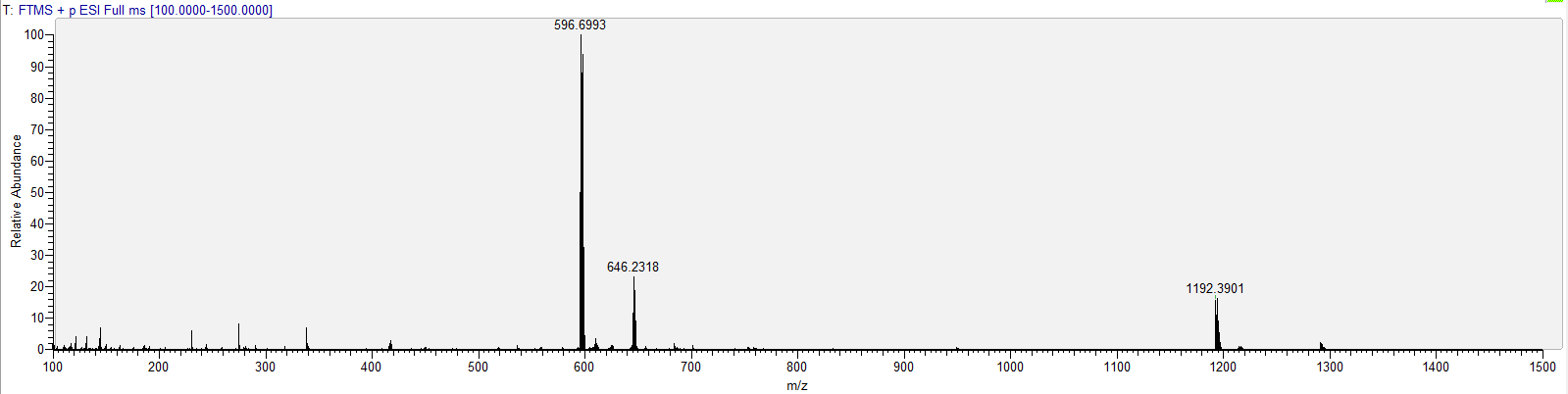


GDAz-15


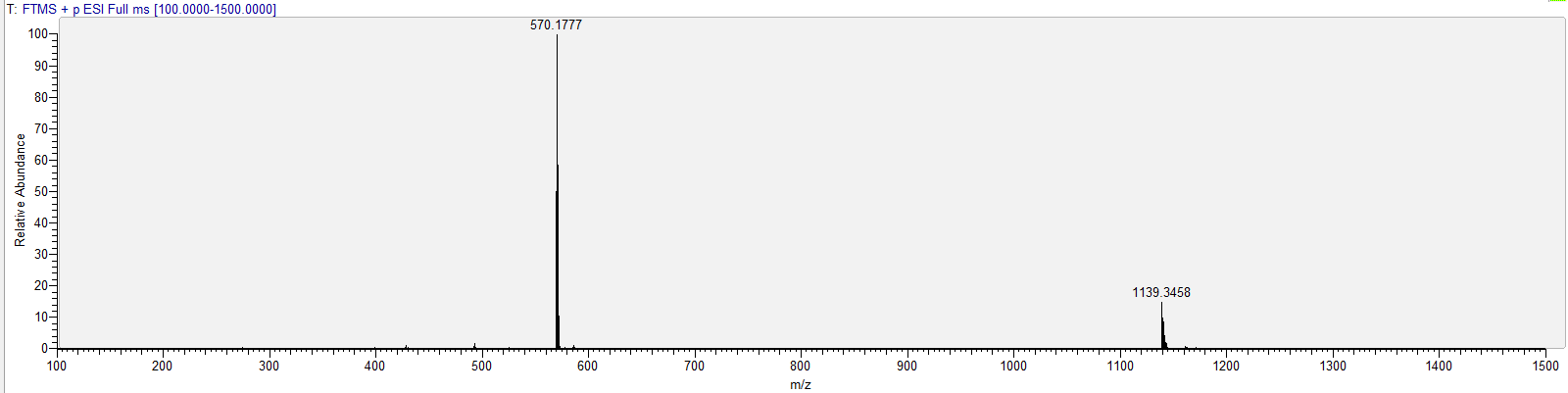


GDAz-16


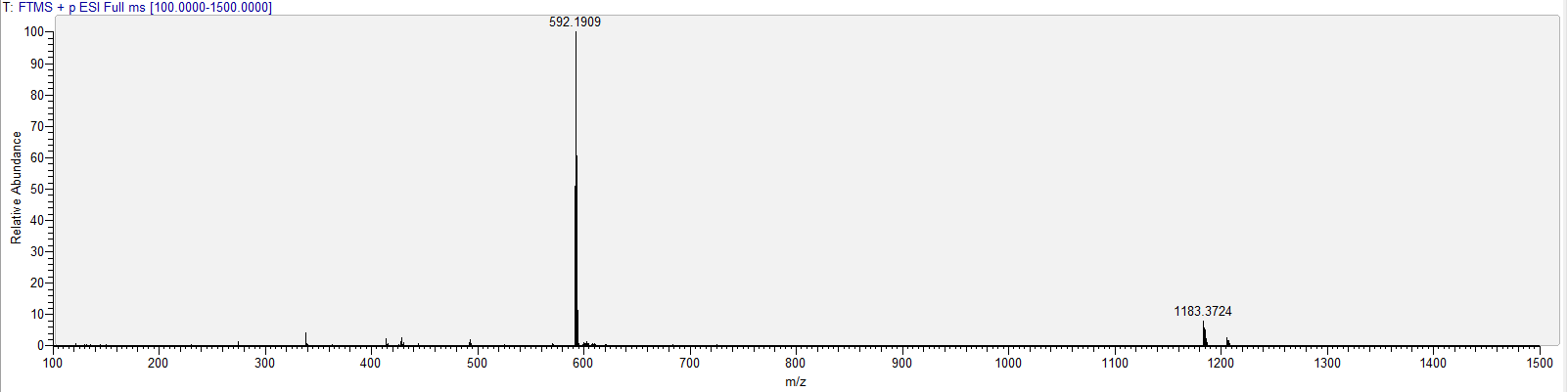


GDAz-17


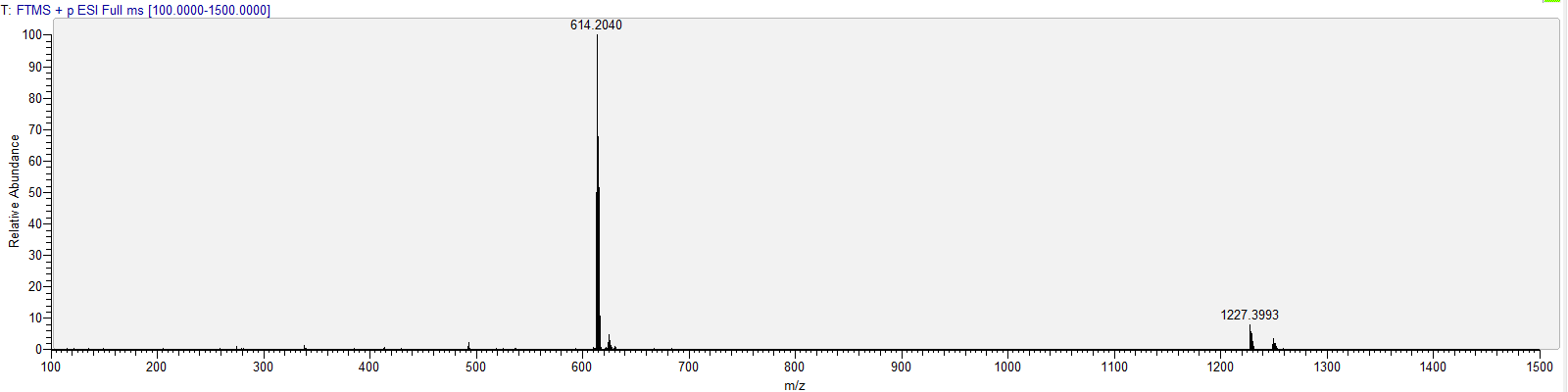


GDAz-biotin


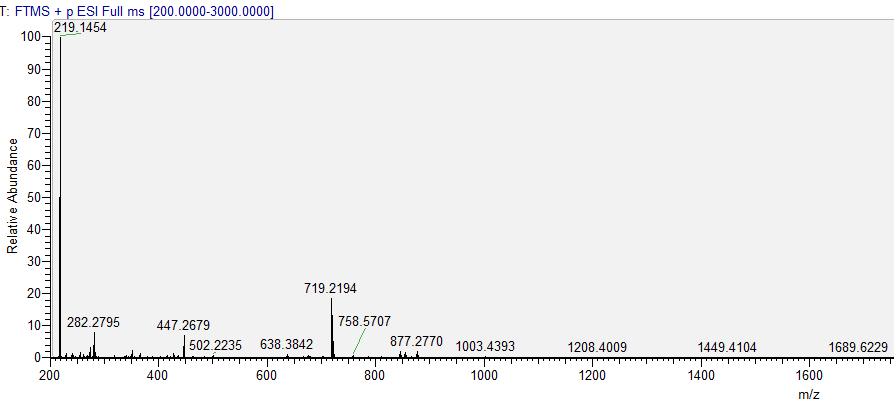


GDAz-OH


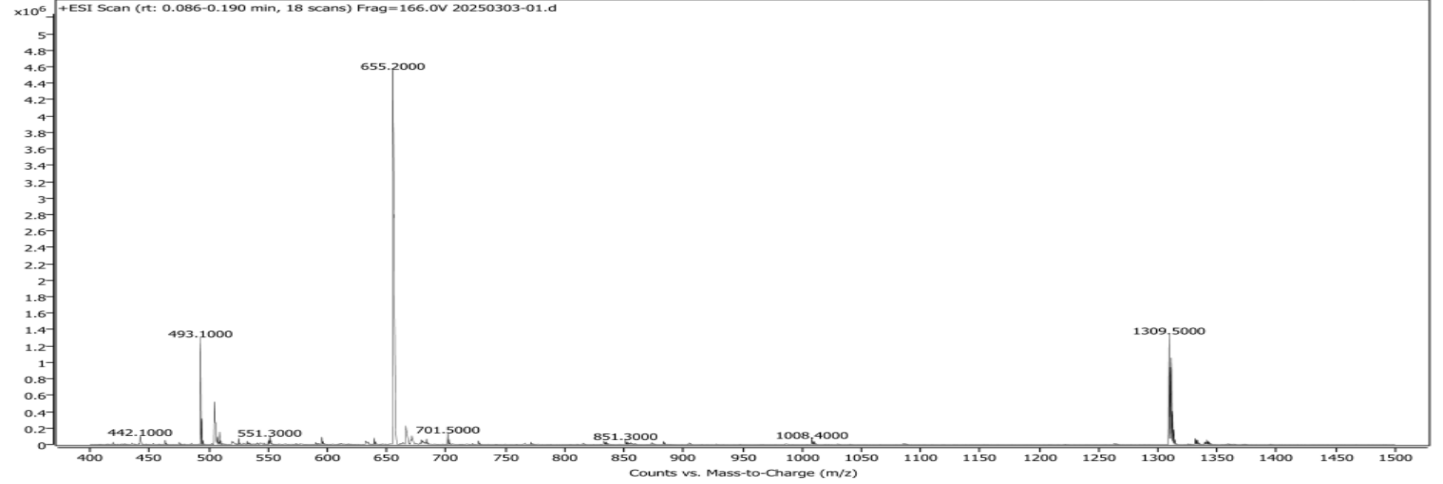


HPLC for target compounds

The purity of the compounds (except compounds GDAz-8~9) was verified by the high-performance liquid chromatography (HPLC) analysis performed on a Hypersil GOLD aQ column (250 mm × 4.6 mm, 5 μm) using a mixture of solvent methanol (A)/water (0.1% H_3_PO_4_, B) with gradient elution (0 min: A/B = 90:10; 0~6 min: A/B = 90:10, v/v; 6~11 min: A/B = 1:99, v/v; 11~18 min: A/B = 1:99, v/v; 18~24 min: A/B = 90:10, v/v; 24~30 min: A/B = 90:10, v/v) at a flow rate of 1.0 mL/min. For compounds GDAz-8~9, the purity analysis was performed on an Amethyst C18-H column (250 mm × 4.6 mm) using a mixture of solvent CH_3_CN (A)/water (1% TEA-CH_3_COOH, B, pH 7.3-7.4) =80/20 at a flow rate of 1.0 mL/min. All compounds were detected at a wavelength of 254 nm.

GDAz-1


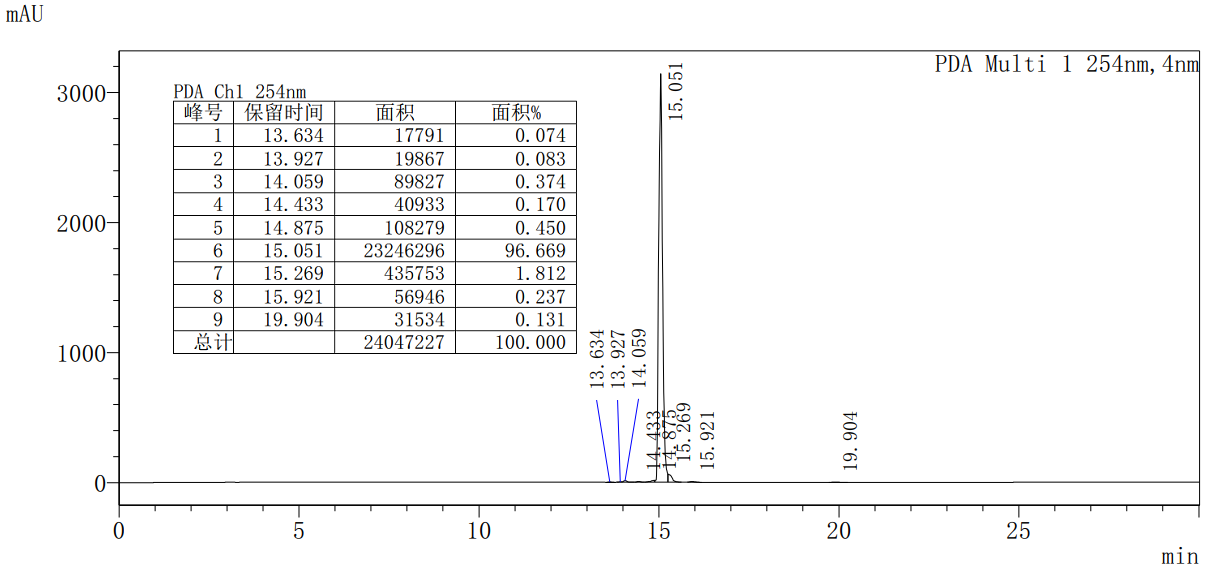


GDAz-2


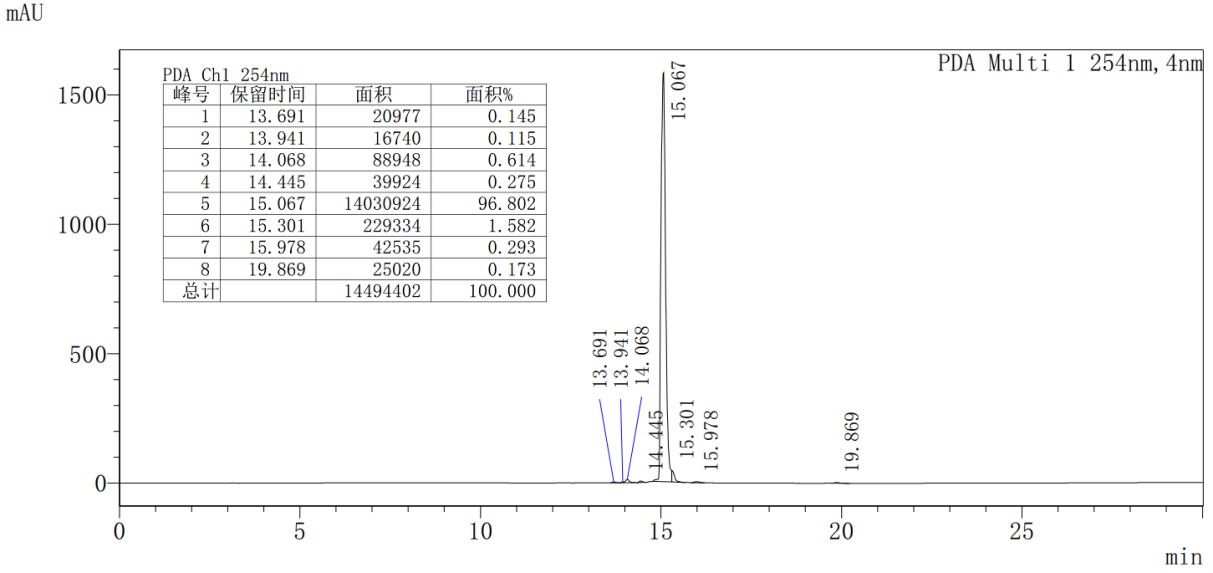
GDAz-3


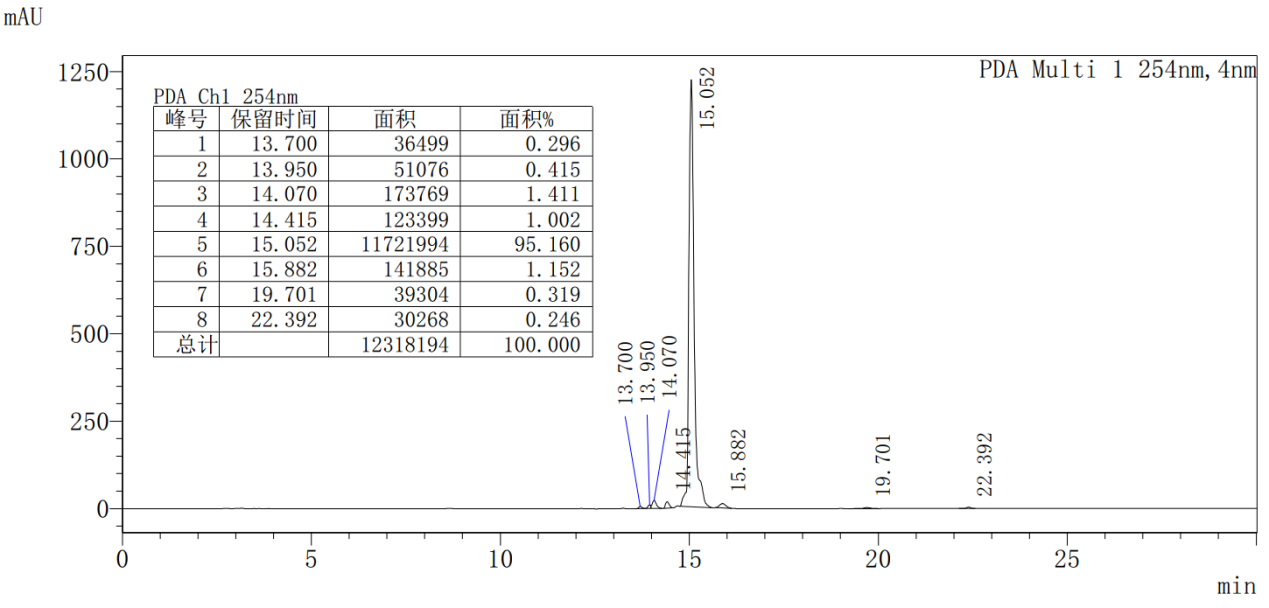


GDAz-4


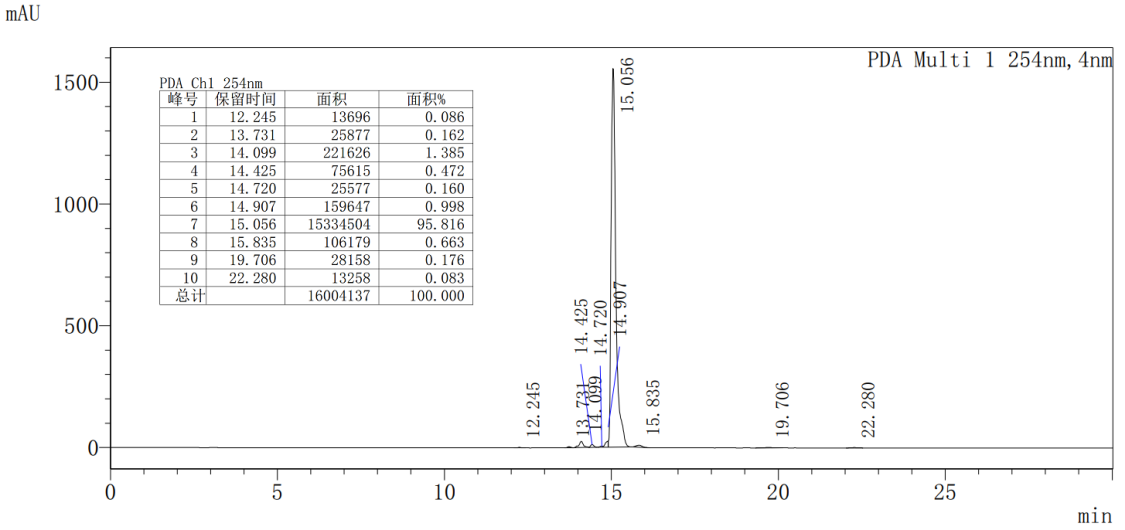


GDAz-5


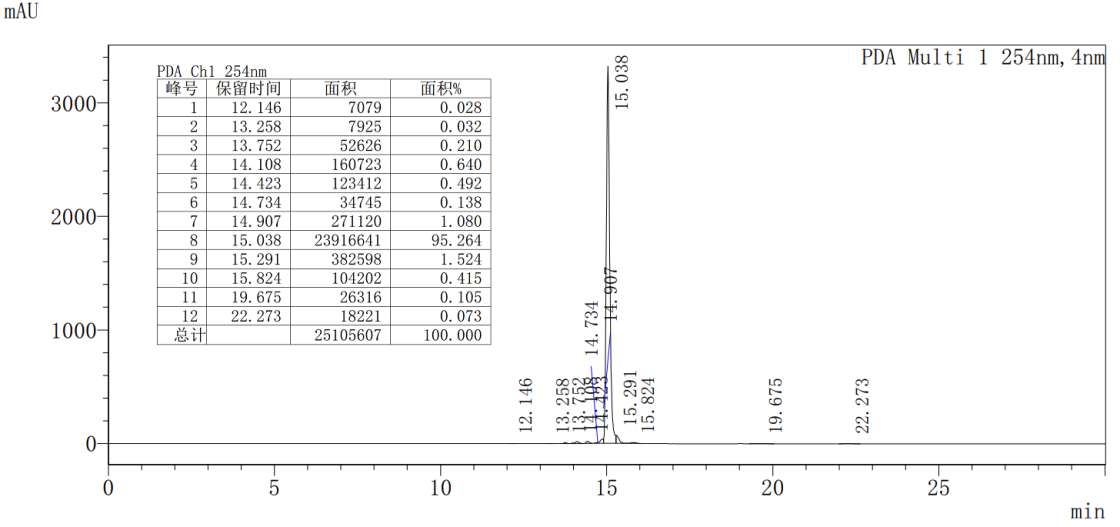


GDAz-6


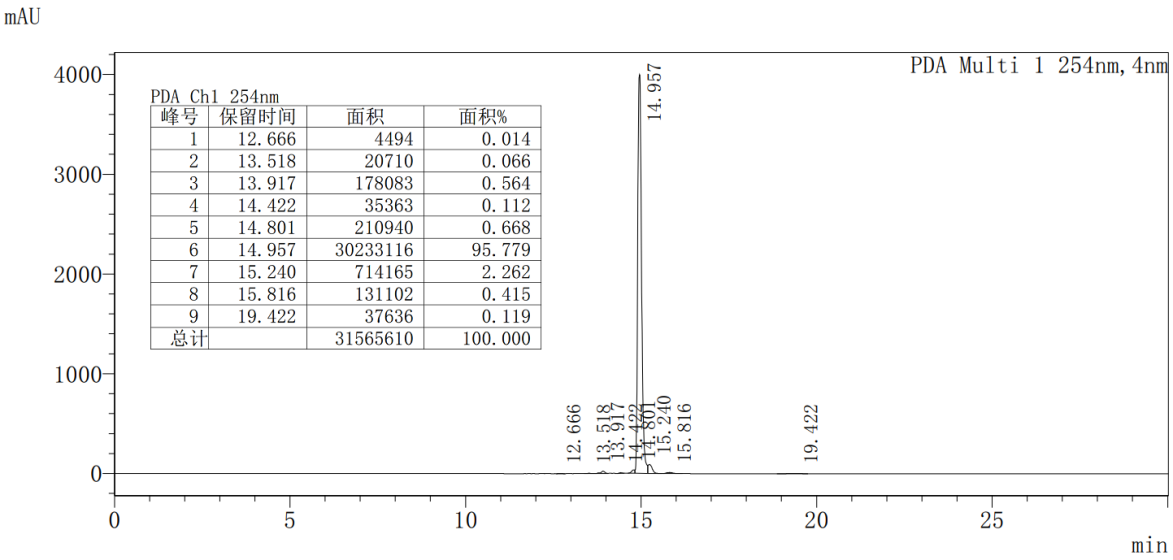


GDAz-7


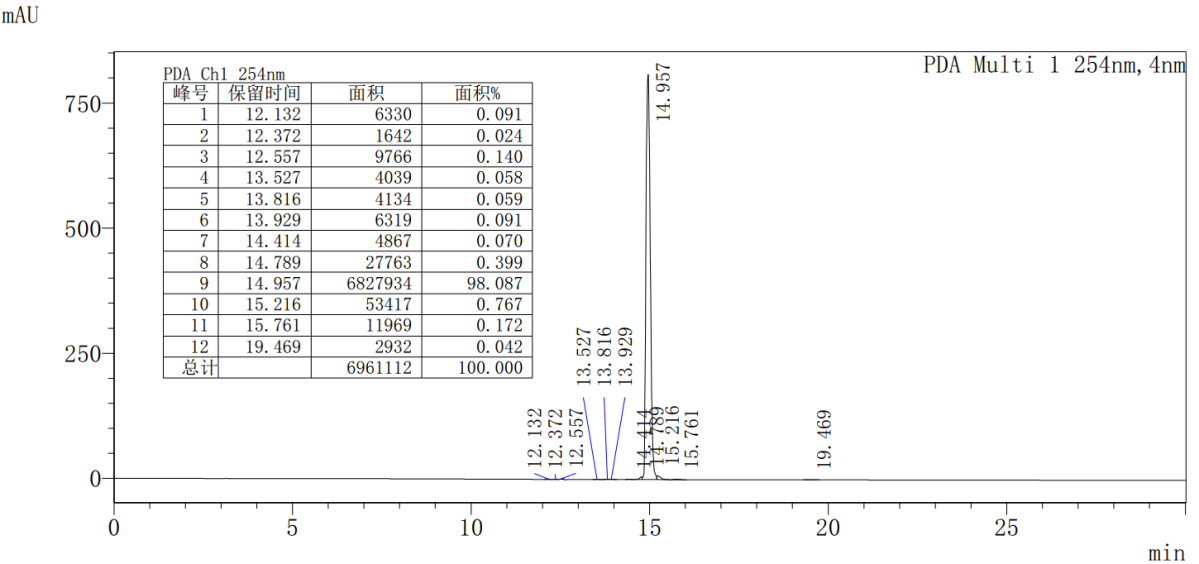


GDAz-8


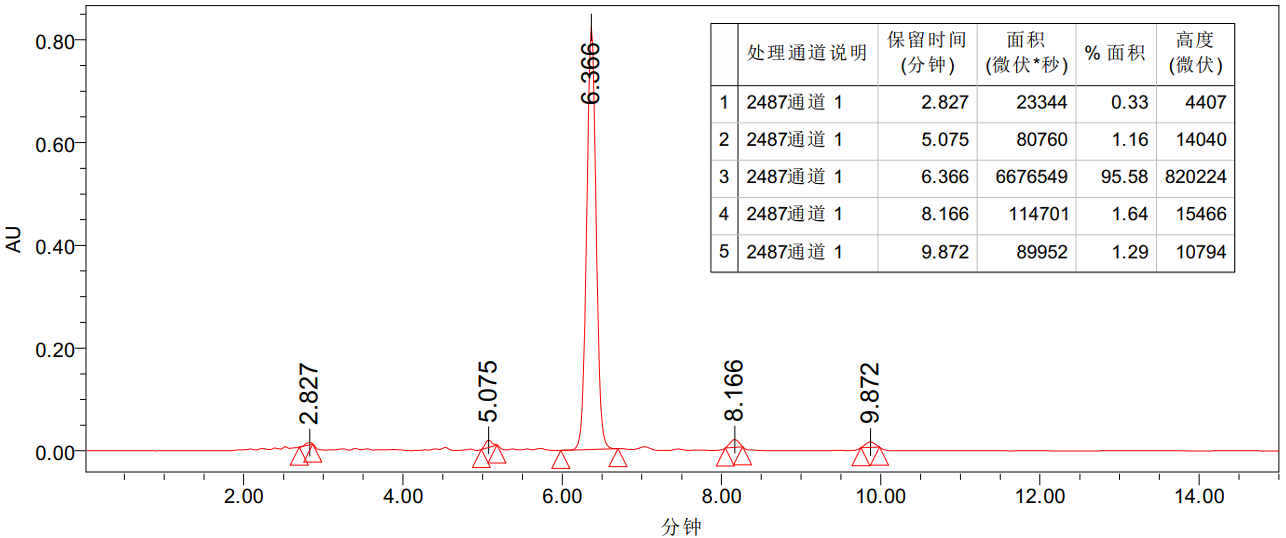


GDAz-9


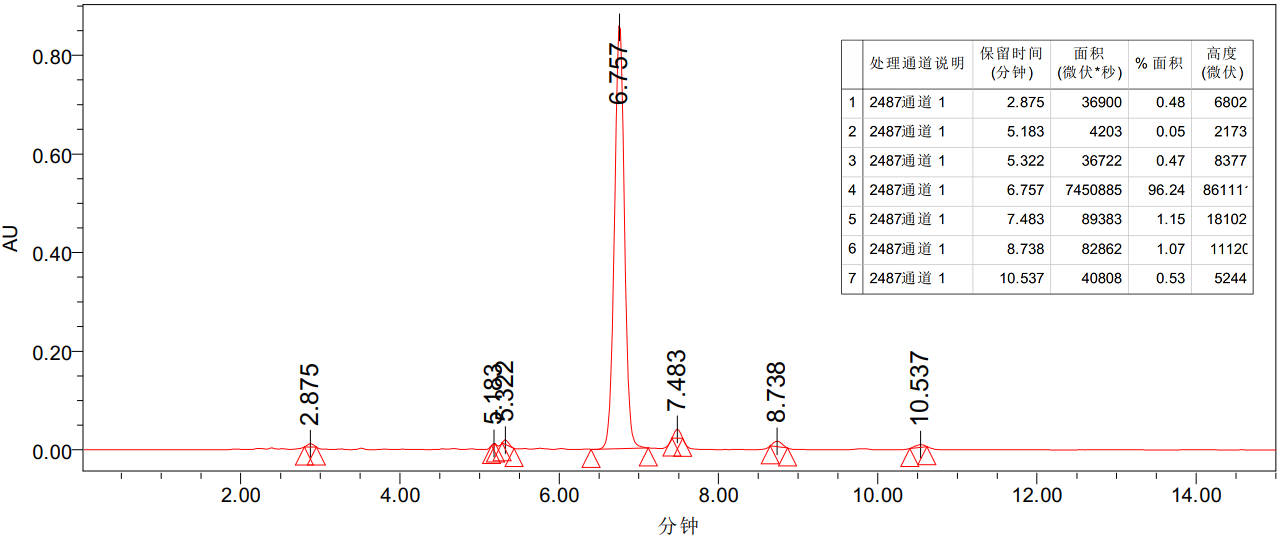


GDAz-10


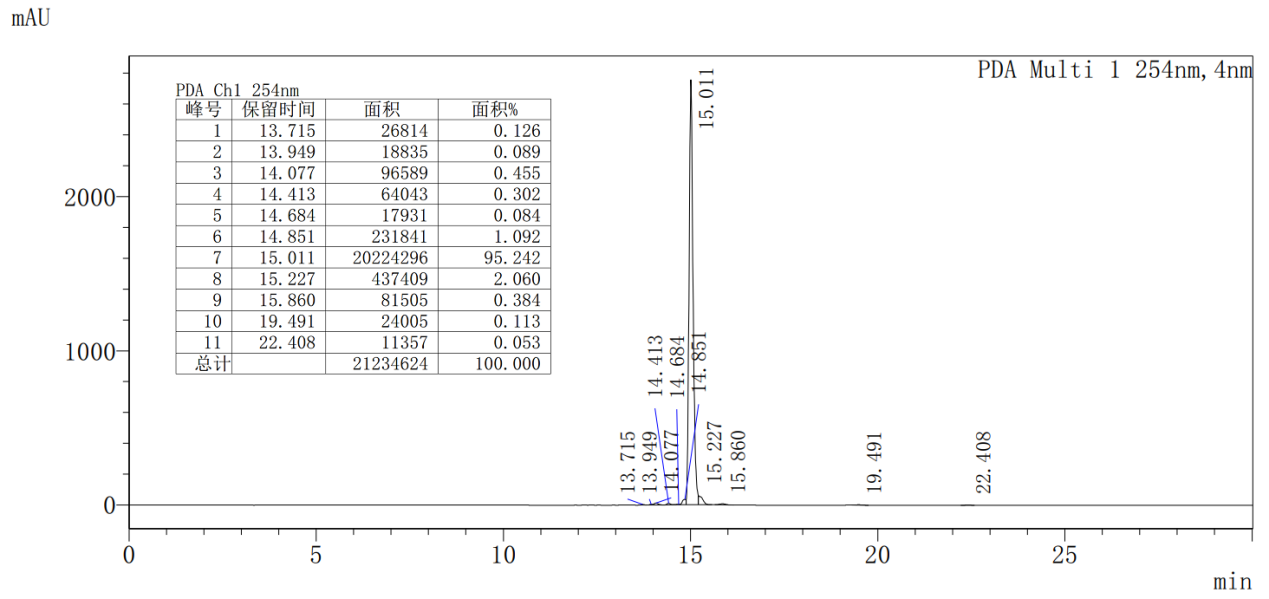


GDAz-11


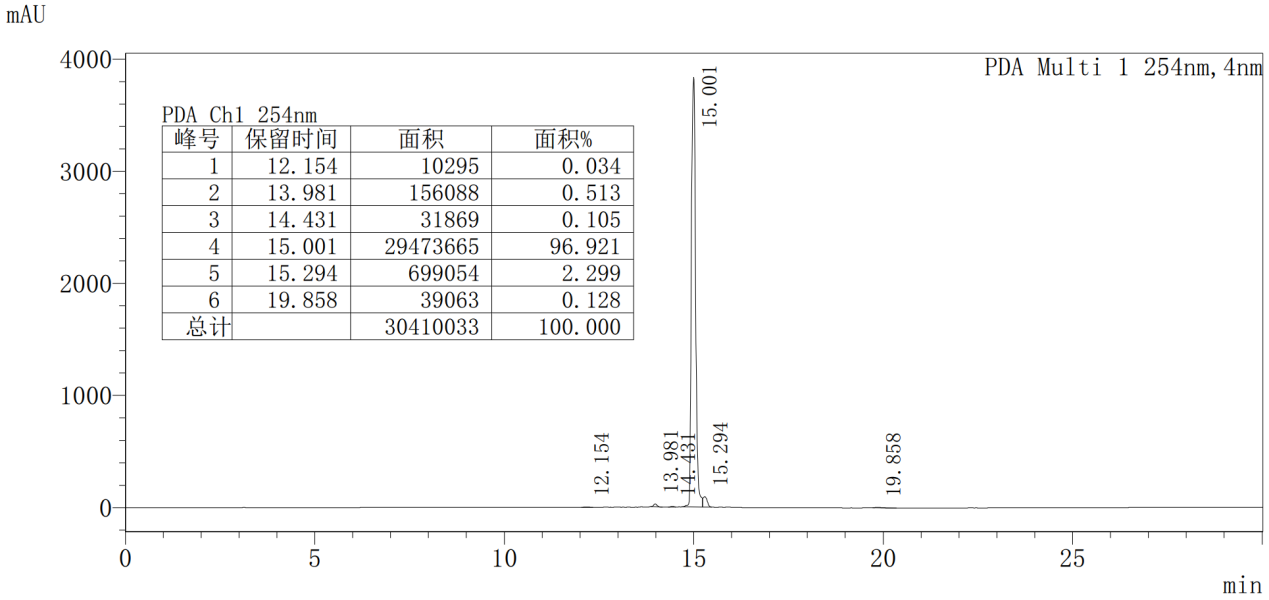


GDAz-12


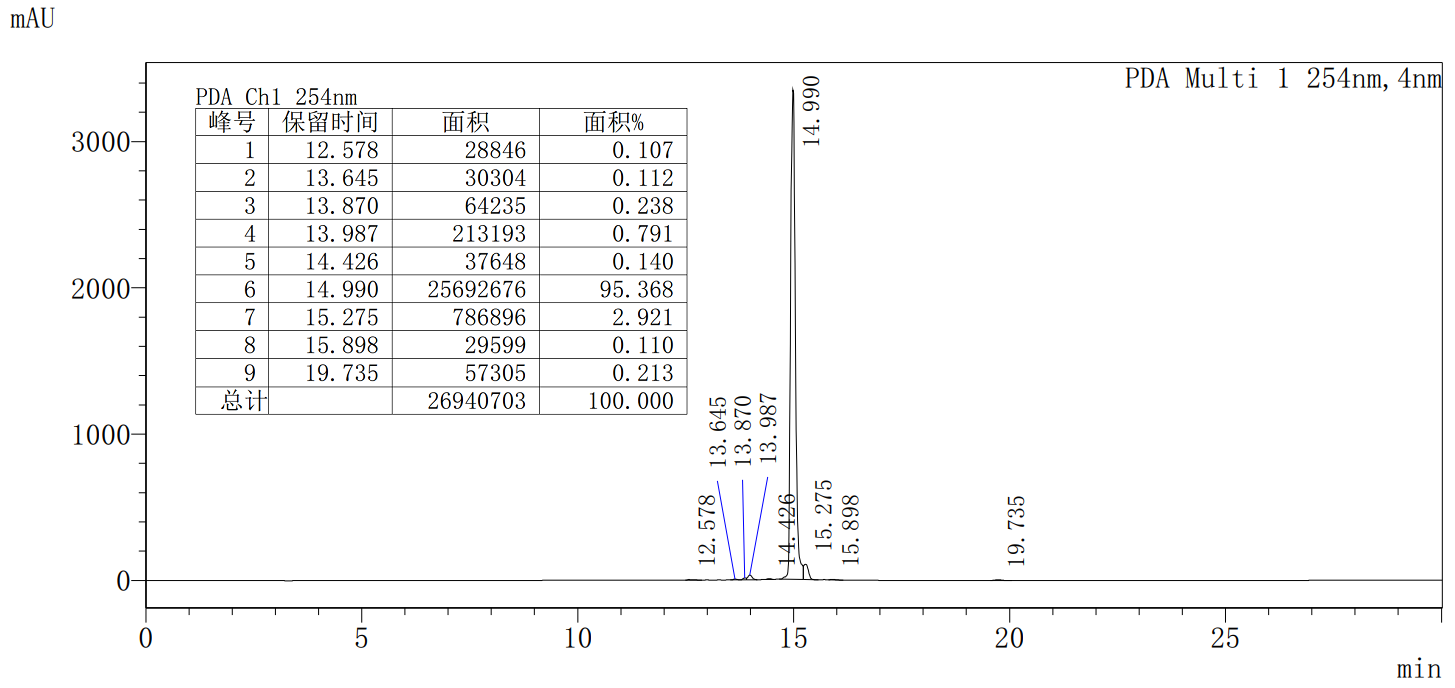


GDAz-13


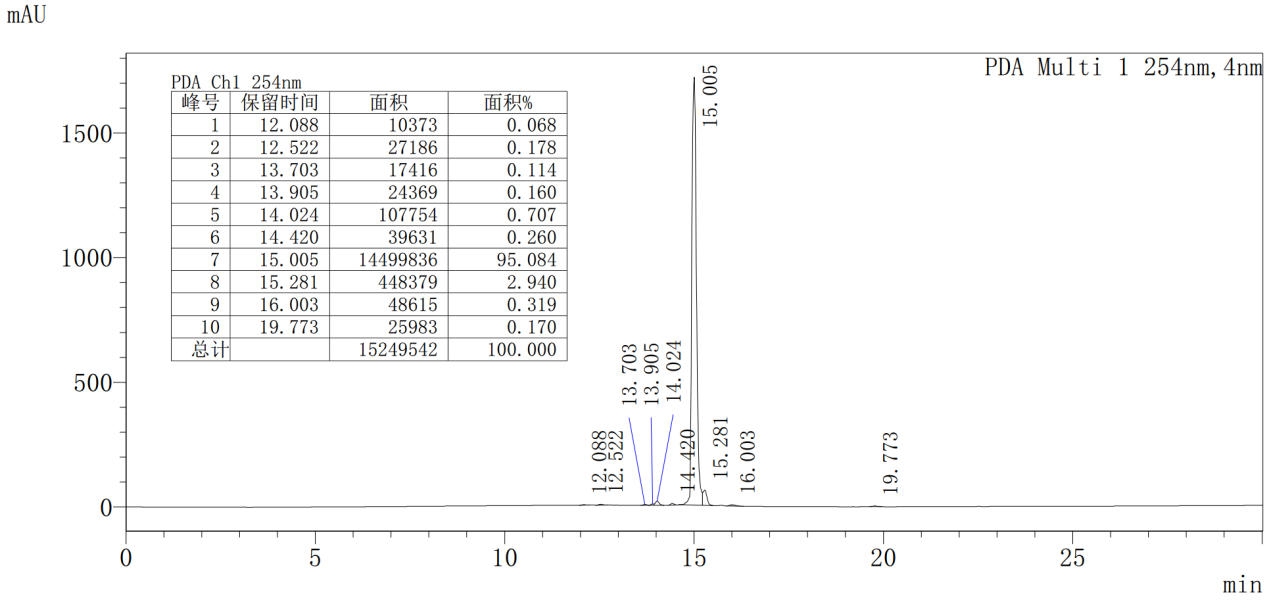


GDAz-14


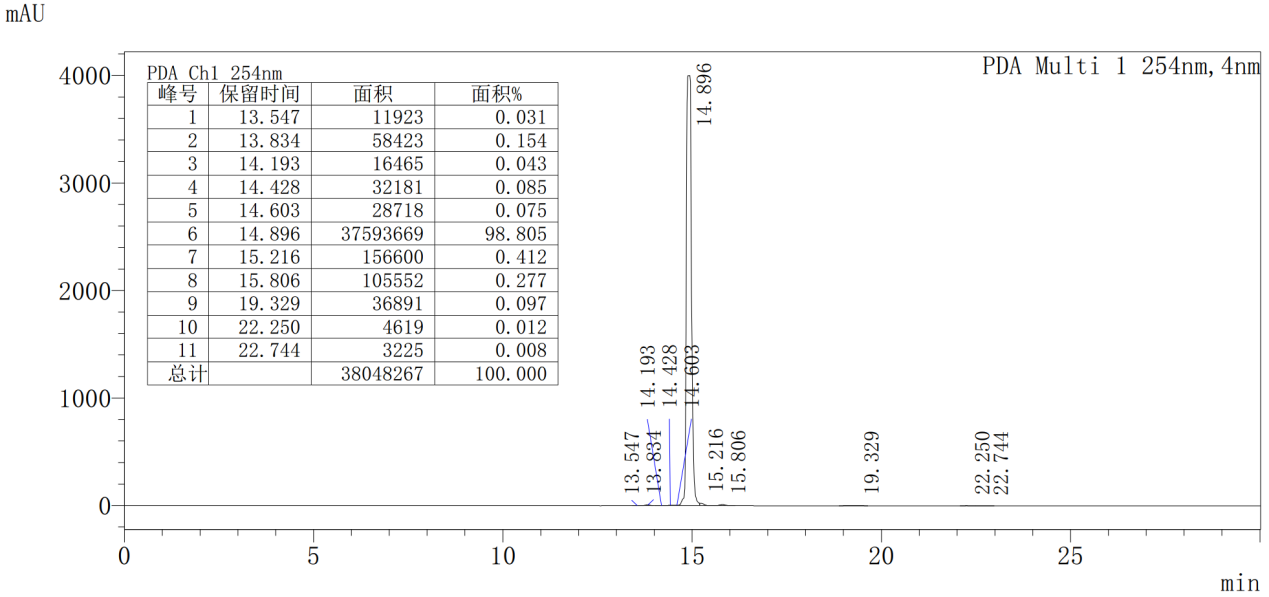


GDAz-Neg2


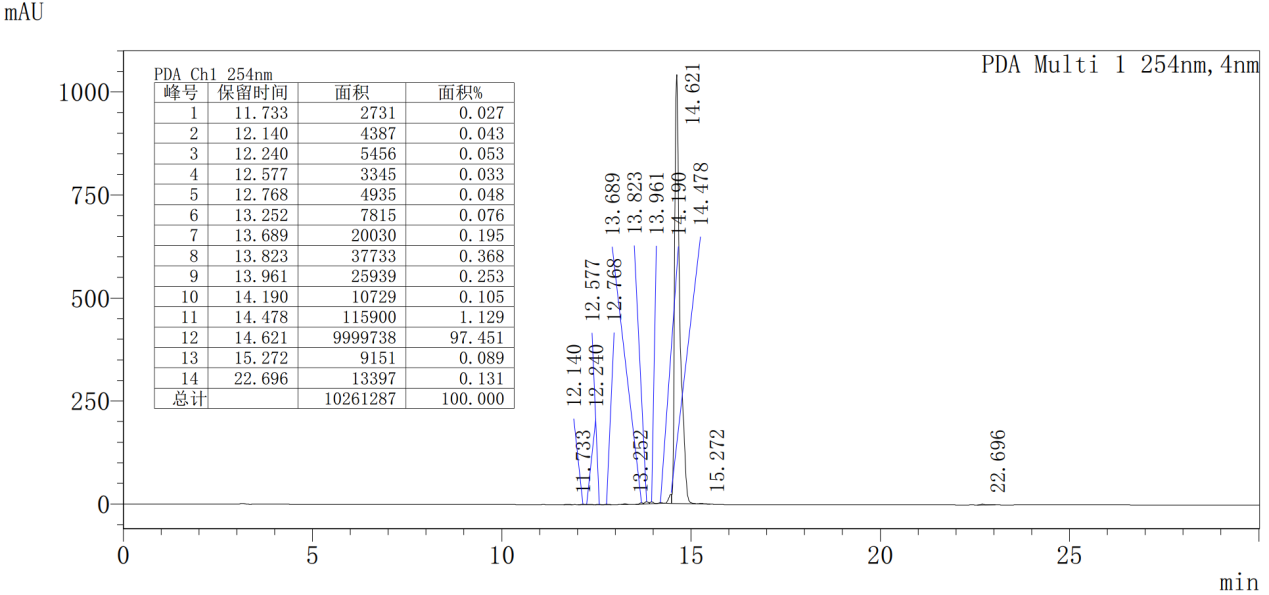


GDAz-15


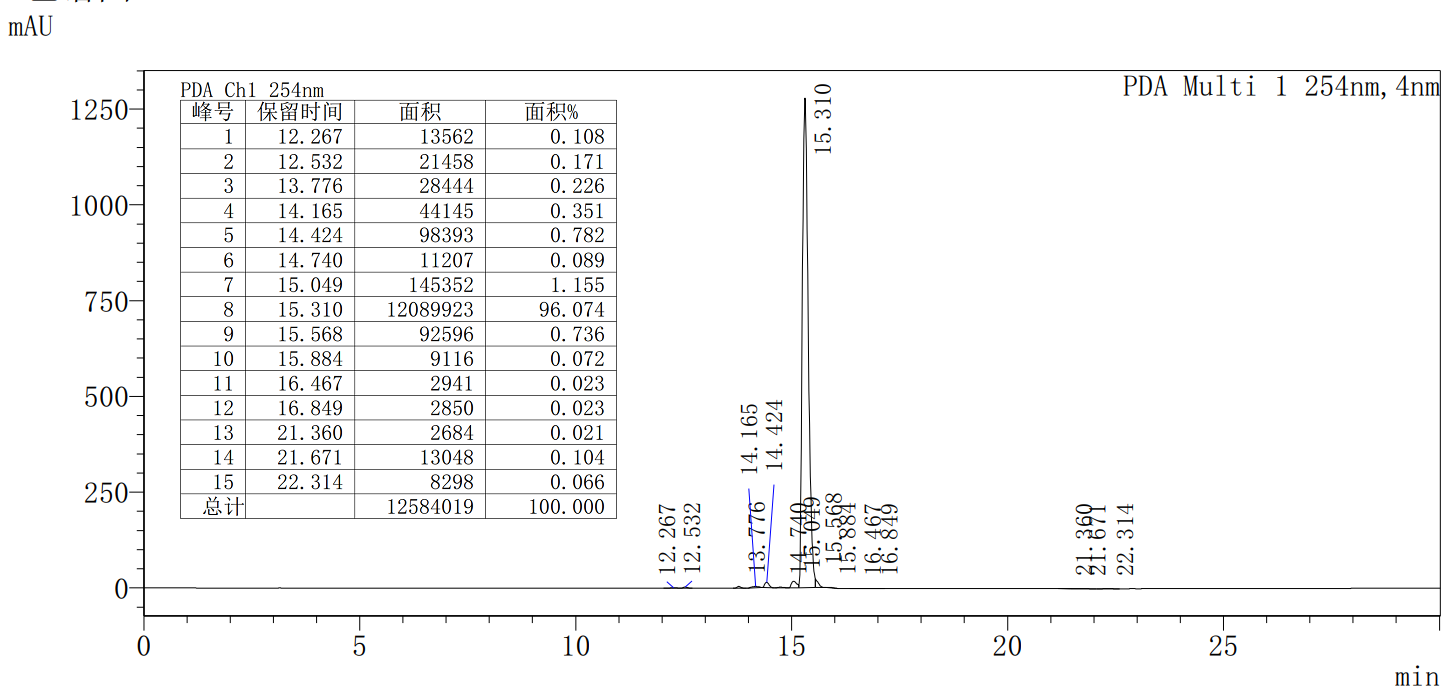


GDAz-16


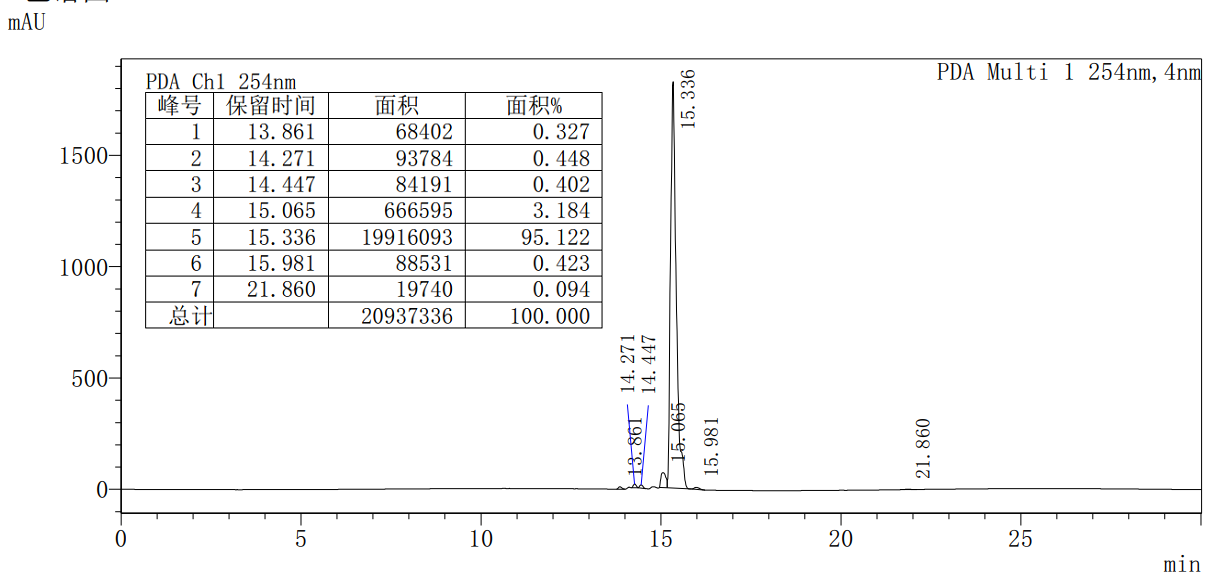


GDAz-17


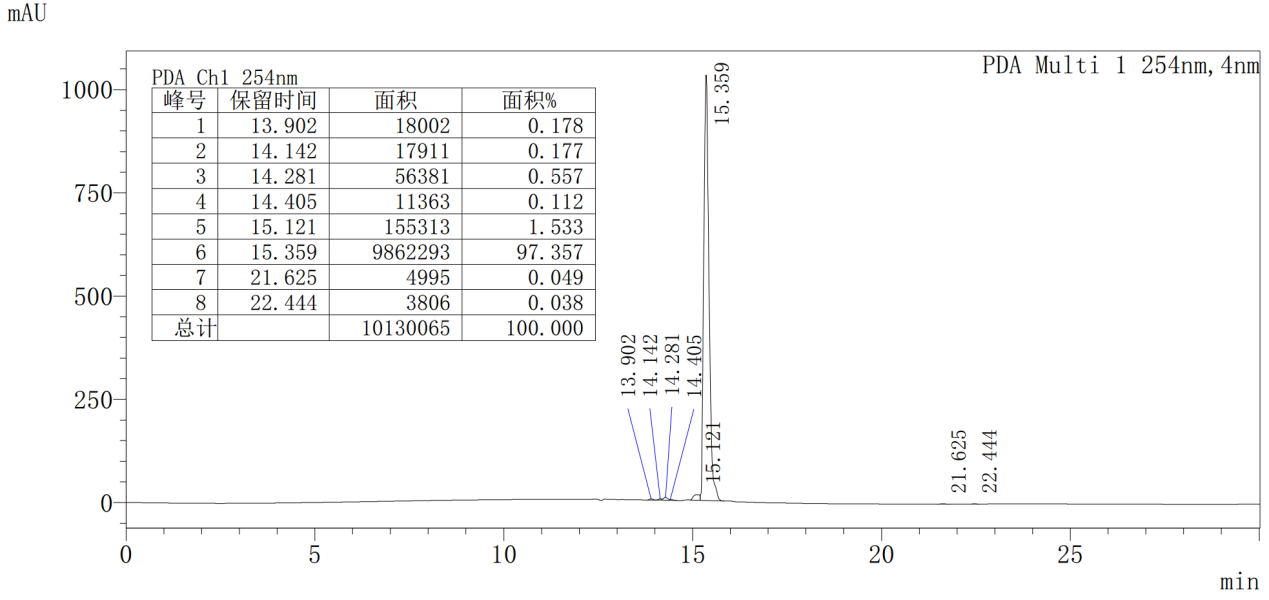

Supplement: Supplementary file 1 — Supporting Information [file ADVS-13-e13655-s001.docx]
